# Supplementary material for: Ragging as an expression of power in a deeply divided society; a qualitative study on students perceptions on the phenomenon of ragging at a Sri Lankan university
Source: PLoS One. 2022 Jul 11;17(7):e0271087. doi: 10.1371/journal.pone.0271087 (PMC9273066; doi:10.1371/journal.pone.0271087)
Supplement: S1 File — (PDF) [file pone.0271087.s001.pdf]

Guide to focus group discussions with the students

The interview guide was developed by the research group as a part of the study and is not copy righted. The interview guide was only developed in English as all the moderators were fluent in English and did not require the interview guide to be translated.

Interview Guide

Focus group interviews will be conducted to extend our knowledge on ragging violence among university students by getting perceptions of different people who work in the university environment or are related to the students and students from different faculties.

Introduction

1. Introduction of facilitators

2. We will explain why we are here: "We want your opinions and ideas on ragging violence among university students as you are students".

3. We will explain how all answers will be treated confidentially. "We are from the University of Jaffna and Uppsala University and conducting research ragging violence in universities. We are not from a government institution and don't have any political agenda. Participation in the discussion is completely voluntary and you do not have to answer any questions that you do not want to answer."

Questions to be discussed about

1. In different types of media we can read about serious types of ragging. You can read such articles frequently. In your group of students do you sometimes discuss these question and what comments do you have? Do you think the discussions in media are true/exaggerated? Are there "good ragging" also?

Probe: what is serious ragging? What is "good ragging"

Probe; what types of violence do your friends talk about?

2. When (what time of the day) and where can these types of violence occur?

Probe; Can you mention the most common places?

3. According to what we have discussed now who are the possible raggers?

Probe; Are they senior male or female students, others at the university, people you know/don't know?

4. Are there any student groups that are more vulnerable to violence?

Probe: any difference if Tamil, Sinhala, Muslim, coming from rural area/urban area, attending famous schools or other schools etc

5. If anyone notices any type of ragging violence among the students, what do you think people will do? Or what is generally done?

Probe; who do you think will act? Other students, university staff, health staff, wardens, or any other?

6. When people in general talk about the university, what do they say? Do they say the university is a safe or unsafe environment for you?

Probe; From where will the general public and parents get their information? If people say good / bad, give reasons?

7. Do you think problem of ragging violence on campus gotten worse, better, or stayed the same in the last couple of years?

Probe; why do you think so

8.There are student unions in the University. Their role in keeping up ragging?

Probe: who are selected as members? Gender distribution? Collaboration between student union and teachers?

9.Young people usually like to test new things. What about the use of alcohol and drugs among university students?

Questions about the Introductory program

8. Have you attended the introductory/orientation program in the university and what does it include? Do senior students take part in the program? If so what is their contribution?

Probe; what do you think about this program?

9. Do you think the orientation program helps in reducing ragging violence among the students?

10. What do you think should be added to the introductory program in order to reduce ragging violence in the University?

Probe; in which way would your recommendations help?

Is there anything you would like to have in your university that is not there yet?

Probe; such as sport facilities, meeting places, one mentor for few students, counsellors outside the university, youth health clinic etc

Finally we would like to know

11. What do you recommend should be done for the students to respect each other both at home and at the university?
