# Supplementary material for: Ragging as an expression of power in a deeply divided society; a qualitative study on students perceptions on the phenomenon of ragging at a Sri Lankan university
Source: PLoS One. 2022 Jul 11;17(7):e0271087. doi: 10.1371/journal.pone.0271087 (PMC9273066; doi:10.1371/journal.pone.0271087)

Types of ragging and Incidents of ragging

Types of Ragging

- Dress code – New students are forced to dress in certain ways when they come to the university, so they could be distinguished from the seniors. Women were forced to wear dresses made from a certain fabric generally worn only at home, called "Cheeta dresses" in Sri Lanka and braid their hair in two plaits in a juvenile manner. This was meant to humiliate and infantilize the women. The men had to shave their heads, wear white long-sleeved shirts, no belts, and no underwear. Both men and women had to wear a certain type of slippers generally referred to as "bathroom slippers" and were not allowed to wear shoes.

- Restrictions – New students are forbidden, by the seniors to go to certain places like the library, canteen, and not allowed to sit on certain benches on the university premises that are reserved for senior students

- Verbal abuse – Seniors use abusive or obscene language with the new students

- Sleep deprivation – Senior students wake the new students in the middle of the night and ask them to perform various task until dawn. They are not allowed to sleep during this time, even if they say they are very sleepy and have classes in the morning

- Physical violence – Seniors slap, beat and kick the new students. Senior students make the new students do vigorous physical exercise and perform tedious tasks

- Sexual violence – Senior students make the new students strip, force them to watch porn and there have been instances where rape occurs

- Forceful initiation into alcohol, smoking and drugs

Incidents of ragging – Videos & newspaper articles

https://www.youtube.com/watch?v=16dYaF6bCDw

https://www.youtube.com/watch?v=hHG_3CArN0M

https://www.dailynews.lk/2021/10/18/features/262175/time-control-evil-ragging

https://www.ft.lk/Columnists/Ragging-is-dragging-Sri-Lanka-down/4-701376

https://www.lankauniversity-news.com/p/sri-lanka-university-ragging.html

Major incidents of ragging in Sri Lanka

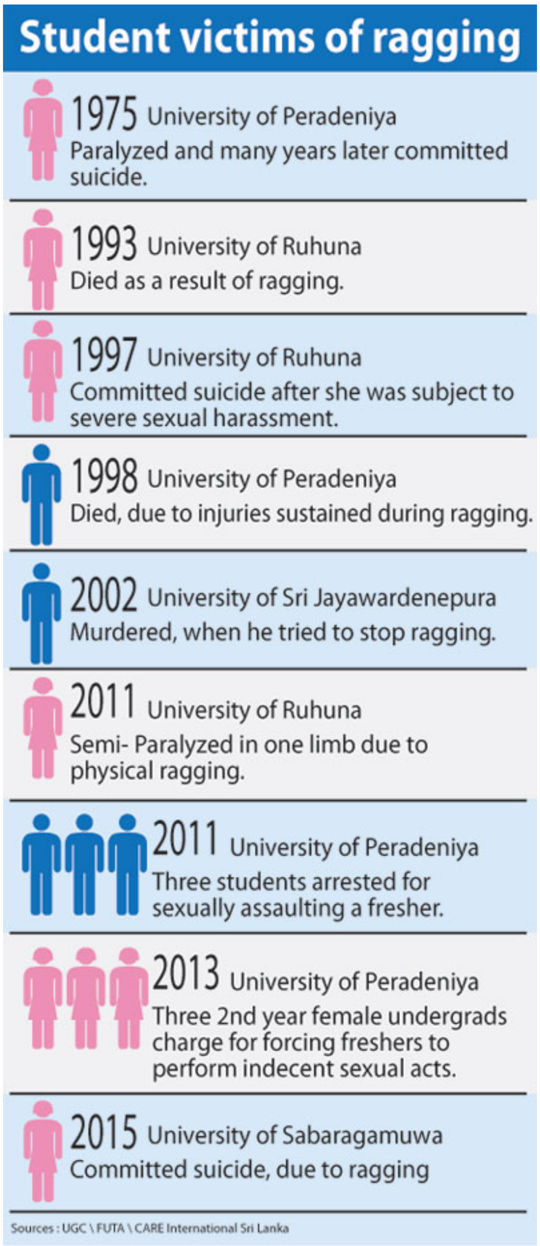

Supplement: S3 File — (PDF) [file pone.0271087.s003.pdf]
