# Supplementary material for: Ragging as an expression of power in a deeply divided society; a qualitative study on students perceptions on the phenomenon of ragging at a Sri Lankan university
Source: PLoS One. 2022 Jul 11;17(7):e0271087. doi: 10.1371/journal.pone.0271087 (PMC9273066; doi:10.1371/journal.pone.0271087)
Supplement: S4 File — (PDF) [file pone.0271087.s004.pdf]

Supporting Information – Transcripts of FGDs

FGD 1

Moderator: explanation of study.
Moderator: In different type of media we can read about serious type of ragging. You can read
such articles frequently. In your group do you sometimes discuss these question?
Everyone: yes
Moderator: what comments do you have?
Person 1: do you mean about ragging?
Moderator: yes.
Person 1: sometimes ragging is more. I red someone got admitted in hospital from some other faculty. There are physical ragging also. So we have talked about it.
Person 2: we didn't have this problem. So we discuss we are escaped.
Person 3: we never experience it. But we heard.
Person 2: yes
Person 3: we never see ragging. But we know.
Moderator: Do you think the discussion in media are true/ exaggerated?
Person 4: they exaggerate. Sometimes they hide truth.
Person 1: sometimes they highlight news. They exaggerate.
Person 3: for a normal news they increase it.
Person 2: some problems are not coming out.
Person 6: they talk over.
Moderator: Are there are good ragging also?
Person 6: yes. sometimes they teach.
Person 3: help to study related things. Some people don't understand that. They think its ragging.
Person1: giving introduction. Initially we don't know anything. First we have to know everyone's name. like that. They identify skills. Who can do sports and dance. Like that
Person 5: that is good ragging.
Moderator: What is serious ragging?
Person 1: yes.
Moderator: what is happening?
Person 2: we didn't see. But we heard
Person 3: initially students who got ragged share it with us. Later they adjust that then now they
cooperate.
Moderator: who? Sinhala students?
Person 3: yes. Now they take it then they will give it to their juniors.
Moderator: what they are doing?
Person 1: they tell physical ragging.
Person 3: they are hurting mentally. If they did a mistake they have to write Sinhala bad words
500 to 100 in a sheet.
Person 4: for using short cuts they have to write bad words 50 times.
Person 5: they cant use English. When they see their seniors they will tell something very long.
Person 4: even when they are late for lectures they will do that.
Moderator: what they will do?

Person 6: they will tell a particular sentence to seniors.
Person 1: if they ask them to come to canteen at 6am. They will wake up at 5.30 then bath and go. If they ask they will tell they are going to study hall to study.
Person 2: they will go to canteen at 6am. But they will let them go from canteen at 7.45 only. So
after that 20minutes distance they will run. Daily they will come to lectures late.
Person 5: they cant use shortcuts.
Person 2: they expect our support.
Person 6: but we are neutral. Our count is very low.
Person 5: its very hard. We are not this side nor that side.
Person 1: with that same dress code they will come to hostel. Infront of seniors they have to follow that code.
Person 4: even if they bath they have to plait they wet hair.
Person 5: mx six months. They will looks very scared. Pitty.
Person 1: during our initial days I saw they force one girl to feed lunch for a boy. I don't know
who is that girl. I don't remember that students because its our initial days.
Moderator: When and where can these types of violence occur?
Person 1: canteen.
Person 2: ground
Person 3: hostel. Now its reduced. In our times senior junior everyone in same hostel. Daily daily
they will do meeting. For 4-5 people from same district one leader. Probe; Can you mention the
most common places? When they got a news first they will pass it to leaders whatsapp group. Then they will pass it to sub groups.
Person 4: whenever they call you have to go. No excuses for sick or exams.
Person 2: when someone don't obey them they will separate that person from batch. Don't involve that person in common events.
Person 1: they are scared of that. They wont share any news with them. They will not involve that person in any functions. They will put names for that person. They wont use our name. they
will use that name in whole university period.
Moderator: where these types of violence occur?
Person 1: canteen. Grounds and their temple. They will take them to temple to clean and they actually rag there.
Moderator: do they even take girls there to rag?
Person 1: yes. They take them to clean then they rag.
Moderator: According to what we have discussed now who are the possible raggers?
Person 2: our seniors are only guiding us. But when it comes to complain whole batch will be affected.
Moderator: do girls also engaged in ragging?
Person 1: they are main. They do more ragging than boys. I didn't saw but I saw sometimes they
talk rude. We don't know what is happening in their rooms.
Person 2: they will scold.
Moderator: you muslim girls are in one room sinhala girls are in one room?
Person 1: no no mixed.
Moderator: is there any differences in tamil, muslim and Sinhala ragging?
Person 1: we don't have ragging. We have only dress code. If we don't follow that they will

look us differently. Maybe they will separate us from batch. Even they did their welcome party
separately. Didn't happen to us. We didn't take ragging so we don't have welcome party. So they
did separately.
Person 2: there was a simple common welcome party. Also they did one special.
Moderator: If anyone notices any type of ragging violence among the students, what do you think people will do? Or what is generally done?
Person 2: we will ask.
Person 1: but they don't do anything in front of us.
Person 2: they know we will complain. If we were they it will look like a casual talk.
Person 4: they will go to canteen 6-7am in morning. Until we go there, there will be a meeting.
Someone will talk. They will just sit there. They don't even take their foods.
Person 1: sometimes they will take food and keep it without eating. It looks pity.
Person 4: we feel bad when we see them. They came early but still they didn't take their food. We will go to canteen around 7.30am for 8 am lecturers. After we left that place again they will
start.
Person 6: mostly they wont do anything in front of us. Secret.
Person 5: they wont expose because they have to do it to their juniors.
Person 4: initially they were against it. They ask our support. They told at this time this ragging
will be going on. So please inform someone. After some time their attachment with us was reduced.
Person 5: batch fit.
Person 4: then they stop sharing.
Person 3: in a meeting we told on that place they support their seniors. So we stop doing that.
Person 1: one of our tamil girl stand up and asked them. But they didn't talk anything. 2 girls got
affected mentally and 1girls dropped her study.
Moderator: do people you don't know are engaged in ragging?
Person 1: they wont allow that.
Person 2: it will become big issue like caste problems (laughter)
Moderator: When people talk about the university, what do they say? Do they say the university
is a safe or unsafe environment for you?
Person 1: yes
Person 2: yes
Person 5: we told we are safe.
Person 4: they know what is happening. They saw news papers.
Person 1: they know our childrens don't do that. Only Sinhala students are doing that.
Person 2 we used to tell them.
Person 1: yes
Moderator: Do you think problem of ragging violence on campus gotten worse, better, or stayed
the same in the last couple of years?
Person 3: increasing
Person 1: some universities are telling its reduced but according to us its getting worst. Because

they will get mad wanted to do more. They are introducing new systems (laughter)
Person 5: whatever they do juniors wont complain they will obey them then later they will do more.
Person 3: we cant change them.
Person 5: even we cant support them.
Moderator: do you have students union?
Person 1: yes.
Moderator: do they help to stop ragging?
Person 1: majority of union members are Sinhala students. No news will come out.
Person 2: if president is tamil secretary is Sinhala. If president is sinhala secrtary will be tamil.
Person 1: but union will run by Sinhala students only. They will support. In march we had a problem in university because of union only. They ask everyone to come to ragging.
Moderator: so they wont help you to reduce ragging.
Person 1: no no.
Person 4: no
Person 3: because they are majority. They will tell if you want some help you have to come to us.
Person 6: they ask us not to go to lectures. But we went to lectures even though we got outer bounce. So they scold us.
Person 1: why did you went? What you have got? Former president scold us. Maybe president or
someone.
Person 2: yes former president.
Moderator: is there any chances for the newly coming students to become addicted to alcohol or
drugs?
Person 5: common between boys
Person 1: yes. We have heard many things. They will force you to drink if you don't they will troll you.
Person 2: like you are not a boy. You are a girl.
Person 4: also in welcome party there is a separate drink party.
Person 5: yes it is there.
Person 4: they will book a separate hotel outside university for that.
Person 6: it will be in night time.
Person 1: girls will also go.
Person 6: boys girls all will go and have fun.
Person 5: If they learn it they will continue.
Moderator: are they using marijuana?
Person 1: we have heard but we didn't see.
Person 2: someone bring it inside and got caught. They said they dismiss that student. It is a not
good for that boys study.
Person 4: if some thing happen to them they will immediately do strikes and do some thing.
Person 5: even if it is their mistake they will do strike.
Moderator: Have you attended the introductory programme in the university and what does it include?
Person 3: they talk about how to study and improve our skills.
Person 2: they introduce university environment, what are the courses, who are lecturers..
Moderator: what is your seniors contribution?

Person 2: welcoming and announcing. Others are done by lecturers.
Moderator: Do you think it helps in reducing ragging violence among the students?
Person 1: no
Person 3: they said you complain. But we couldn't do that.
Person 1: they gave website phone numbers. They give student counsellors number. They took
our signatures a form which tells that we are against ragging. But still they are doing ragging.
Moderator: What do you think should be added to the introductory program in order to reduce ragging violence at your university?
Person 1: after orientation period we will go to lectures. There is no chance to rag during lectures.
Person 2: you can tell the seniors not to rag before we come. Also you have to tell the juniors don't get ragging. To stop giving ragging first they should not accept ragging.
Person 3: if someone rag us obviously we will think we should give back. I think everything should be controlled earlier. Our seniors are the first batch of our faculty. If you stop them from
being ragged they would have not give it.
Person 1: they told seniors from other faculties ragged them. They got affected more
Person 3: 2 faculties ragged them.
Moderator: Is there anything you would like to have in your university that is not there yet?
Everyone : transportation (laughter)
Person 3: faculty is very far from hostel.
Person 1: we have to go here and there within 30 minutes.
Person 3: its 25 minutes distance.
Person 1: within 1 hour we have to go hostel, pray and eat then go back. If we pray we cant eat.
Person 5: interval is only 1 hour. 25 minutes we have to walk then refresh pray, eat then again go
back to practical or lectures for 25 minutes.
Person 3: its very hard for us.
Person 1: we need transportation.
Person 3: we cant study. We will be very much tired. We will feel sleepy. Even in nights we cant
study.
Person 1: other than that little ok.
Person 4: sports facilities.
Person 1: yes. But we don't know about sports. To practice they have to go to main campus. Its
impossible. Here sports facilities are very low.
Moderator: do you want anything else? Health clinic?
Person 1: yes we have in Tuesday and Thursday.
Moderator: What do you recommend should be done for the students to respect each other both
at home and at the university?
Person 2: are you asking about communication.
Moderator: everything.
Person 1: first thing seniors should not accept ragging.
Person 5: seniors are expecting respect from juniors. But they thinks they can get it only by ragging. That is the problem. So they rag and take their respect.
Person 3: they think if they control them in first year they will be controlled for future.

Person 1: they can see the respect only in face. Inside how much they scold. We only know. Other than that they cant get anything.
Moderator: what kind of ragging Sinhala students are doing?
Person 6: physically and mentally.
Moderator: what they are doing physically?
Person 1: I have heard that a girl should propose a boy. Then…
Person 2: I saw they were eating something one by one passing.
Person 1: some kind of touching also going on. We never see. But they told us.
Person 2: dog (laughter)
Moderator: what dog?
Person 1: you tell
Person 2: they imitate how 2 dog do sex.
Person 1: after that they will come and cry. They will be mentally affected more.
Person 2: some ask girls to draw boys body parts and boys to draw girls body parts.
Person 1: they don't take all girls. They will select some girls. 4 boys took 10 girls. We will be
scared when heard them.
Person 2: it is like face to face. If they cry they will scold them by bad words.
Person 1: but after welcome party they will become very close.
Person 2: yes. They will tell it is fun. They now feel bored.
Person 1: but now they become very much fit.
Person 4: if a problem for them all seniors will come there and stand for them.
Person 2: but now when our batch mates rag our seniors, our direct seniors are helping them and
guiding them how to do ragging. How to escape when they got caught. We don't know but they
have a support outside. That's why they are doing everything with courage.
Person 2: cant do anything for them. They will become more fit.
Person 1: we will become fools. 4-5 sinhala students share everything with us. So a tamil girl fight for them. But they didn't tell anything in public. She felt very bad. They will talk behind but in front they are close to their seniors.
Person 2: a senior brother ask if you have any problem tell us. But they are scared. They will fight then they will become more closer than us. If one have a problem everyone will be there. Immediately they will do strikes. If any strike 1 or 2 will go to lectures but all of them will do strike at a time.
Person 1: even after this problems they will become more fit.

FDG 2

Moderator: Introduction. What is your opinion when you hear the word ragging?

Person 1: It is an unwanted thing…. because some people think it is a fun….. just like this their doing to their juniors…. Hmmm… spend their time…. But if we take the juniors… their side…. Definitely they will be affected by ragging….. mentally and physically…. If you take our university…. They affect physically….. the physical violence also…..

Moderator: What do you mean by physical violence?

Person 1: They slap them…… they brutally slap!

Moderator: What do you think?

Person 2: It's a bad thing…. but in a way…..to make people friendly…… in a way…. If it is in a superficial level it is ok but as she mentioned…. If they are going to be beaten or physically abused…. Then that time…. It's a real hard time for the juniors… and the other thing is…. They take revenge when they… they wait for their chance to take revenge others…..

Several Giggling…

Person 2: ….. that is the most horrible thing…I think…

Moderator: What do you'll think?

Person 3: exactly! I firmly agree her one….. they might think… the seniors might think… they think that they want to make the juniors friendly with them…. in that way they might beat them and some kind of things…. I have also heard of such things like that….. so, I also firmly agree that they should make other think….. they should make seniors think… that the seniors should be friendly with the juniors….. I think…. Ragging is also some kind of funny or….. but beating and slapping at them….. and in front of other girls….. if it is a boy in front of other girls it will be a greater problem for them…. their respect and all those things…. they will be quite… ahhhh… they will be feel…. Ahmmm… uncomfortable in front of them….. so like that…

Moderator: What do you?

Person 4: It is also a bad thing….. I think ragging is a good thing because juniors and seniors want to communicate with…… for a good relationship….. but in our university….. they have very different…. They slap…. Everything is very different….. I saw….. one of my lecture time…. I saw a seniors came to our room and….. ahhhh… and coming…. Ahhh… and asked the boys to come in front and….. slapped the… all boys in front of us….. while we are….

Moderator: Why? Do you know why they are slapped or they just came and slapped?

Person 4: …They just slapped….

Person 1: …..without doing anything…..

Person 3:…. That is there….

Person 1: …..without any valid reason or any reasons….

Person 4: …they didn't tell any reason….. but they slapped…. We are…. Afraid of them…. after the… after they…. Went we informed our lecturers…… she has punished them….

Moderator: Was it one boy or a group of boys?

Person 1: group of boys….

Person 2: …..in the name of friendship…..they do these type of things…

Person 3: ….they think that these type of things will help…..

Person 2: …and the juniors waiting for their chance….. their opportunity…

Moderator: But those coming and slapping them, where they also studying English or were they from other faculties?

Person 1: from other faculties….

Person 2: …. other faculties….

Person 1: … I haven't had any ragging from our department….. (smiling)…

Person 2: ….not form our department…..

Person 3: …..we are the seniors….

Person 4: …..not form our department…

Moderator: Exactly! You have a very small department.

Several together…… yeah…..

Moderator: But usually this ragging…..

Person 1: Not only the university mam'…..

Person 2: …they do….. stepping out of the university also….

Several people talking together… Hostels also…… (Unclear)….

Moderator: What do they do?

Several talking together….

Person 1: …Slaves…

Person 2: …..actually in our hostel….. what exactly happens there…. They ahhh… they take us to the seniors….. they just want to show their seniority to us….. that means…. They should make a distinction between us…. They are seniors…. They are 3rd year and 4th year…. We are first year….. so, we have to obey them…. and hardly (strictly) we have to obey them….. they expect it from us…. So in the night even…. Last semester.. we were called…. We were requested to come around 9.00 O'clock in the night… and they left us around 12 O'clock…. Even we said that we are really feeling sleepy…. They just don't listen to those stuff and they are just going on themselves…. Whatever your sleepy….. you have to…. Even if your sleepy you have to sleep on the floor…. That's all you want to do…. So we have to wait until they say…..

Moderator: What do they ask you to do? From 9-12? Do they tell you to….

Person 1: Just sitting….

Person 2: ….. they just talk and talk….. that's all….

Moderator: They just talk (laughing)? Do they ask you to make Tea and wash their clothes?

Person 1: No… not like that…..

Person 2: …Yeah….. in our hostel…. We haven't heard even….. the warden is there… then she will prevent us….. the… not such kind of…. Problems…… they are just calling us…. For the meeting at 9 O'clock….. that means that sleepy hours….

Person 3: In our case….. we didn't face any ragging….. but these things we….

Person 4: …..because we are teachers….. and we get…. External… after… one year external degree….. then only we enter the university….. so we are little bit strangers to them…..

Everybody laughing…..

Person 4: …… isolated…. Isolated…

Everybody laughing….

Moderator: Are they frightened?

Person 1: No, no they are not frightened….. they respect….

Moderator: They know at one that this is a teacher.

Person 2: ….they respect us….

Moderator: We have heard that Tamils rag only Tamils and Muslims only rag Muslims…….

Person 1: Yeah….yeah….

Person 2: …..yeah…… yes..

Person 3: ..actually there is….

Person 1: ….there are some processes no….. Muslims have to wear….. they have some discrimination between batches….. Muslims have to wear these black 'Parada'….in the first year….

Several talking together….. (unclear)….

Person 3: …..even in the first year we have to wear this… actually…. You know that 'Kalutara' (area close to Colombo) people…. How Muslims wear there….. we are not allowed to wear colorful stuff….

Person 1:… black….. not colorful….

Person 3: …… colorful 'Abayas' even… so we just have to wear….

Person 4: …..so we have to just wear this…. even our seniors ask us to cover up ourselves….. but… whatever they want us to wear…. This one (showing Black Abaya)…

Moderator: So like in Kalutara, what if you don't want to cover, can you do that? Or is it not an option?

Person 1: …Actually our….. there is a group, association in our university called the 'Muslim Masjid'….. foe each and every university they have….. so, they have asked us to do this one…. But sometimes….. it is some kind of prevention…. As well as our seniors wants us to make ourselves covered like this….. and not to show our beauty to others…. That these things we are…. Wearing and all those things….. so that's what's happening there….

Moderator: Alright, so, it's sort of controlled by the seniors.

Person 1: seniors…..
Person 2: Exactly…… controlled by girl seniors……

Laughing….

**New comers two students walk in…..2nd year students who live in Jaffna

Moderator: So, reintroduction to new comers…. What is your understanding of ragging? What comes to your mind?

Silence…….

Moderator: So, we will just talk and you will feel comfortable.

Everyone laughing…..

Moderator: Gives a quick recap of what was said. Have you seen other people, like Sinhalese girls, what their seniors do?

Person 1: …. There also they have some discrimination…… 1st year girls, they have to wear these cotton clothes and this….. with puffed sleeves and all….

Several laughing…..

Others echoing the same thing

Person 1: …..like small children…. They treat them……

Person 2: ….. And they have to carry their books….. only the books…… not in a cover or backpack…..just to bring their books only….. but they can use files…. That paper ones….

Person 3: ….2nd year only they can use files….

Everyone laughing….

Person 4: ….. 2nd year only….

Person 3: ..and they have to go in lines….

Everyone laughing….

Moderator: Yes, we have seen them going in lines….

Person 1: They are first years…. Freshers….

Moderator: In the hostel, are you staying in the hostel? Alright you are staying at home but you are in the hostels. So, there are Sinhalese and Tamil girls and everybody. How are their seniors? Do they ask them to make tea, wash clothes?
Person 1: No….. we have not seen those things…..

Person 2: …even Sinhalese or like that ……we have not seen….

Moderator: Maybe they ask them to come and talk and talk?

Person 1: Yes…. They talk and talk…..and even if they do not plait their hair….. they have to plait their hair in front of them….. that's what they ask us to do…

Person 2: …even if we go to the bathroom we have to plait our hair….. even in the hostel there is another rule…. They have put that we can only wear cotton 'Salwar'…. The least cost 'Salwar'…. And the 'sorti'(?)….

Several laughing….

Person 3: …..and the 'Sorti'….. (laughing)…. Long gown….. maxi or something….

Laughing…..

Person 2: …they have asked us to …… we cannot do to …..wear costly tops or denim or leggings…… we cannot….

Person 4: ….. These days….. we do not have harsh punishments…. Like these…..small kind of…. Yeah…..

Person 3: ….. Slapping only…. Physical abuse…

Person 2: …even for girls they have not slapped…..

Several together…… only for the boys…..

Person 3: …. Recently…. 2-3 days before…. One boy wants to stop ….his studies….

Several echo this statement….

Person 3: …. In university…… because…. Of slapping…. First year student….

Moderator: Which faculty was he from ?

Several together…. Arts faculty….

Moderator: When you were juniors, were you ragged? They said they were not ragged because they were teachers, but were you all ragged? This information is strictly confidential.

Person 1: …Don't be scared…

Moderator: The information will be anonymous and we have actually spoken to a lot of people and we can't remember who said what.

Everyone laughing….

Moderator: Don't worry nothing is going out of this room.

Person 1: Yes, we know…

Moderator: When you all were juniors did you have any problem with ragging….. were you asked to wear something?

Person 1: We were asked to wear flared skirts…. We are not allowed to wear T-shirts….

Moderator: Ok, only blouses. Is it long skirts that you had to wear? Or Knee length?

Person 1: Knee length…. (unclear)

Person 2: … they also asked us to wear the black 'Bata' slippers…. the bathroom slippers….

Laughing….

Moderator: How long did you have to wear like that?

Several together…… 6 months….

Person 1: … for 2 weeks they have to wear saree….

Several talking together…. (Unclear)

Person 2: ….there was a cultural week..

Person 1: … cultural week…..

Moderator: But that is also another way to….

Person 1: ….yeah…. yeah….

Moderator: These days we have seen people in sarees and boys in 'vetthi'

Person 1: ……sarees and all…

Person 2: ….2 plaits… and 'pottu' and 'thirunu'….

Moderator: you might not know but do you think it's getting worse and worse?

Person 1: No….. it's becoming better….

Several agreeing….

Person 1: …. compared to the old days….. and when we compare with the other universities…… here lot of things from other universities…. for example, Peradeniya and those areas…. Physical abuse is much more than the university of Jaffna…..

Moderator: I don't know if you have seen or heard, but apart from the physical abuse have you heard of any sexual abuse?

Several together…… no, no… not here… we have never heard….

Moderator: Not in any other faculties…

Several together….. no, no….

Person 1: …they like to have some casual talk….. and they want to delay the people…

Laughing…. And several talking together….

Person 2:…They want to show their powers….

Laughing…

Moderator: We have heard, that they are not allowed to go to the bathroom, canteen….

Person 1: ….Yeah for the 1st years….

Person 2: …..no…..

Person1: ..for the first year we are not allowed to go to the canteen….

Person 2: …canteen… and library also no?

Person 3: Library we can go…

Person 4: …for the 1st 2-3 months….

Person 3: …. for the 1st month they did not allow us to go to the library…. They said it's a strange place….

Everyone laughing….

Person 3: …..not to the canteen also…. But we used to go…..

Moderator: cant use the library for the 1st month but can use the toilets.

**Loud hammering noise…. Conversations inaudible and unclear

Moderator: Bathroom they have never said not to use?

Several together….. no, no.. they have not said…. Not that abused….

Moderator: That's good. What do you think? What are the common places these things happen?

Person 1: They have a notion…. That the first years cannot sit in particular areas….. they cannot sit and enjoy….. they have separate places for 3rd year and final year…. Like that….so the benches…. So the first years cannot go and sit there….

Person 2: …..even we cannot go and sit there…… even the 2nd years can't sit there…

Several talking together…… (unclear)……

Person 3: …..only 3rd year and 4th year……

Person 4: …. There is a distinction between the 3rd year and the 4th year…… right?

Person 5: …there are separate places also….

Moderator: So, you have places like this is for 2nd year, this is for 3rd year like that. What about cell phones? We heard that sometimes boys will call the girls and ask them to talk for a long time, and to put reloads…

Person 1: We have heard this happening in other universities…. but not in Jaffna….

Person 2: ….but sometimes the boys….. they ask for money from the juniors….. some money…. Like Rs. 100 or Rs. 200….. they get money….

Person 3: Yeah….. from the boys….

Moderator: What do you think are the worst places that people get ragged? Is it the hostels, or playgrounds, or…..

Person 1: Hostels I think…..

Person 2: …Hostels are the worst….

Person 3: ….Even in the hostels we have asked…. From the seniors to call them and….. that means through our money…. Call them and talk for 1 hour…. They just talk ……they just even do their work even…..

Moderator: What do you have to do?

Person 3: …one of my friends…. She experienced…. She called the senior in front of us…. She called her and she was just doing her work….. she said… hold on a minute….. wait a minute…. I'll come back…… and she was waiting and waiting continuously….. and after half an hour only….. she asked, "ahhh… what's your name?" …. "So, say now….. what are the subjects you're going to take?"…..

Person 4: …..authoritive voice…

Person 3: ….. only for the classes or something….. only showing their seniority…. She asked her to… draw a money….. and put her name on to the face and her name is….. Fathima like that and then she has to…. Fathima is gorandu(?) Fathima is gornadu(?)……

Person 4: …..monkey….

Person 3: ….like that and she has to post it on her status…. Whatsapp status….. that is what she asked to do…..

Laughing……

Moderator: That is very embarrassing, I see. Is there a specific time that they do this? You said 9 O'clock, but do they do these things early morning also? Or only evenings after classes?

Person 1: After lectures….

Person 2: …. After the lectures… I think the main….. the core subjects…. They have in the auditorium…. The group of students…. So, after the class, the seniors will be waiting outside…. They will come and…..

Person 3: ….they can easily…. Catch the boys…..

Moderator: So, these seniors, they don't have classes that they are there all the time?

Laughing….

Person 1: ..always roaming for first years….

Person 2: ……they are ….(unclear)…..

Person 3: …..some…… they cut the classes….. and…. (laughing)….

Moderator: So, obviously ragging is more important than the classes.

Person 1: They are very curious… in making them ragging….

Moderator: Do they still tell you things because your 2nd years, in the Arts faculty, always one batch senior… not your batch but other seniors, do they come and say things?
Person 1: No the thing is….. the group of people… how do they connect to the juniors is they call that we are from the same place…. Our home town…. So, they create a group saying that we are from this community or this hometown…. If I'm a student from Trincomalee… I am forced to join our group….. called the university students of Trincomalee… but I didn't connect myself to that community…. So I didn't have any interaction with them…. so I didn't have any ragging from them…. so, I think the disconnection of the juniors and seniors…. First year could lead to…. Could lessen this kind of ragging….

Moderator: We have heard that as soon as you get chosen to the university they will give you a call and ask you to talk to them. Is that true?

Person 1: …..No…..

Person 2: they didn't…. I think it's next day ….. we only have to … introduce ourselves to them….. we don't have to… compulsorily call them… or do this….. these girls and these boys just…. Get the contact to the seniors…. It's not a compulsory thing… that we have to connect ourselves to them…. we didn't connect ourselves….right?.... we didn't connect so our seniors didn't do any ragging to us….

Several laughing….

Moderator: I thought when you get the cut off, they get your number and say that you have to talk…….

Several…. Not in our faculty…..

Person 1: Once we finish the registration only they will come and….. say us to take the numbers from seniors and call us….like that…

Moderator: But before that it's nothing like that? I think you will be excluded from….. But is it like that for boys?

Person 1: ……no ……we haven't heard like that….

Moderator: You haven't even heard for boys?

Person 1: One of my brother's got selected…. To the university of Peradeniya… he kept calling to his seniors and talking….. so the boys keep talking to them without knowing the name even… (laughing) ….. they won't tell their names even….

Moderator: But they call first?

Person 1: Yeah… they call first…. They got the name from somewhere and they called him….

Person 2: ….I think they have a group and from that group they will call…. They have a particular SIM for these…. He will call from one number but several people will talk from that number….

Everyone laughing…..

Person 1: That kind of ragging is there in Peradeniya…..

Moderator: Most of the time who rags? The seniors only rag or do you get ragged by demonstrators and other people?

Several together……. No…… not demonstrators…..

Person 1: They will protect us actually….

Moderator: So, the demonstrators will protect you'll, that's good.

Laughing…..

Moderator: But its always males to males and females to females?

Person 1: Yeah….

Moderator: Males won't go and rag females?

Person 1: …. no….. They just…. Males just want to talk… with the girls….

Person 2: …. That's all…..

Person 1: … "memorize this ones name"….. "this ones name"…. "tell"

Person 3: ……and "call me"…. "call me"….

Laughing…

Person 1: Like that….. then…. "Give me some money"…. They will take their purse and get something….. like that small things…..

Moderator: So, it's a good chance for them to get to know the girls. Otherwise they will not get this chance, something like that?

Everyone laughing…

Moderator: Do you think that there is any group of students that get ragged more than other people? Like, because of your ethnicity or caste or socioeconomic status or where you come from? Some people say that people who come from Colombo get ragged more. Some say that the Sinhalese get ragged more. Some people say, no the Tamil people get ragged more? What do you all think? Do you think there is a difference?

Person 1: Actually, in our faculty…. I haven't seen like that…. a group of people has been ragged like that…. but I have only seen that a group of Sinhalese have got ragging like this….. in the management….

Person 2: …..yes, in the management….

Person 1: … Especially in science….

Moderator: So, only in science and management. This may be more Sinhalese there? Is it like that?

Person 1: because they get social before us….. that's why…. There's another thing also….. for the first year student they will get social in the beginning of the 2nd year…..

Person 2: ….welcome party…..

Person 1: …welcome party….. exactly…. That is called social…. And for the management….

Moderator: This is for the management faculty?

Person 1: ……yes… for the management faculty…. We are Arts….. so for the management faculty… they will get in one semester I think…… so like that…

Moderator: But even after the welcome party, they still rag?

Person 1: No… after the welcome party they wont rag……

Person 2: ….no they won't……

Person 3: ….they are not supposed to rag….. (laughing)….

Laughing….

Person 4: …..the reason why the ragging has lessened….. is ….. these people are threatened…. If you rag and somebody inform the lecturers or somebody…. Then they will be…… punishes from the….. UGC it seems….. so that's why it's less…..

Person 2: ……now there is a hotline number also……

Several together… yeah….yeah….

Person 4: …..That's why these people….. are very silent…. Yeah they are scared…..

Moderator: Do you think people actually call and complain or go to their lecturers, who do they complain to?

Person 1: They have some Marshals…..

Person 2: ……student counselors,

Person 1: …..student counselors and these people….. yeah….. so if they complain….. then those people will be in trouble…..

Person 3: ….. if it is a serious issue only they will complain…..

Person 1: …yeah…..

Person 4: …they will not complain about slapping and those things….

Moderator: If you'll see somebody or say a lecturer sees that, now say if they come to class and slap and if a lecturer sees or something, will they try to stop?

Person 1: someone….. some lecturers…… will ask what happened here…. But someone don't bother….

Person 2: ……yeah…. They don't bother…..

Moderator: They will pretend they didn't see and go off like that? who stops it, if the marshals see it, will they stop it for sure?

Person 1: …yes…..

Person 2: If marshal comes these people will run away….. laughing….

Everyone laughing…..

Moderator: So, the Marshal is quite effective. What about, we hear the seniors say, that if they see any ragging or something bad, "we will stop it". So, do the students stop it sometimes? Say some 3rd year students are ragging the 2nd year students, will some other 3rd year students or 4th year students come and stop this?

Person 1: If they are relatives they will sometimes stop it…..

Person 2: …..sometimes…..

Everyone laughing…..

Moderator: Relatives or from the same area?

Person 1: …. Sometimes…

Person 2: …. Or who are known…..

Moderator: otherwise, they will also say, no you have your fun.
Person 1: …… they make fun of this……

Moderator: Sometimes they think it's funny or things like that. what do you think of Jaffna university? Do they say it's a safe place?

Person 1: It's totally safe….

Person 2:…. Exactly…

Moderator: Why is that?

Person 1: because when you compare with other universities…. I have heard a lot of things before coming to university…. They say that you have to do these things….. and you have to spend money a lot….. that means for this ragging and all things….. but I didn't feel anything in our university… only one thing….. I'm not really comfortable talking in English with our seniors…… they will not allow me to talk in English…. That is the only problem I'm having…..

Moderator: During the ragging period I think you cant talk English.

Person 1: …..we have to only talk in Tamil…. Even for the exact English word we have to say the exact Tamil word….. sometimes we might not know that word also….

Laughing……

Moderator: Yes, even I don't know the exact word in Sinhalese. Where your parents happy that you came here because your from very far away?

Person 1: My mama is very happy… laughing….

Moderator: Your mother is happy? Yes, that you came here. You all chose Jaffna university because the education is good?

Person 1: Exactly….

Moderator: Any other special reason you chose Jaffna?

Person 1: Our working station is here….. so it's close by….

Moderator: I have heard the Jaffna university education system is very good and the lecturers are good.

Person 1: Yes…. Exactly…..

Moderator: That's very good. Because you live far away, do you go home very often?

Person 1: No…. we just go twice a month… or once a month…..

Person 2: ….. if there is a huge leave…. We will go….

Moderator: So, when you are staying here during weekends, I think its not such a big problem for Tamil people living here, when people stay far, when people stay in the hostels during weekends is the ragging more?

Person 1: No…

Person 2: ….. no…

Person 3: … only at the beginning it was like that….. now…. we didn't feel anything like that…. we're just free….

Moderator: I don't know if you know much about this, but is there a lot of Alcohol and drug use among the seniors?

Person 1: Some people they…. They use… some boys….

Person 2: ..reduced….

Moderator: Why has it reduced?

Person 1: When we came here as a first year students…. We were scared…. Because our seniors in that period…. Were always drinking and were lying on the corridors and……. At that time….

Laughing….

Person 1: ….but now we couldn't see any of the seniors drinking and all the….. and doing all those thing….. they are behaving well….

Person 2: ….. their….. mentality has changed I think…. Because of this problem and their….

Moderator: We heard that there is drug use and everything? Maybe you don't know because you are girls.

Person 1: I have not seen anyone in the university….

Person 2: … we heard about it but we have not seen…. We did not have any experience…..

Moderator: Have you heard, I don't know if you have but is there a place called 'Welikada' in the university?

Person 1: The prison….

Moderator: They call it 'Welikada' like the prison where, if they say "come to Welikada, that means that they are going to beat you.
Person 1: We don't know….

Person 2: ….I don't know…..

Person 3: …. The girls… boys might have known something….. if the boy comes you could have get some more information…..

Person 4: …..boys… I have heard that they….. Science boys exactly…. They will be called to the science laboratory…… and they will beat you….

Person 5: ….I have heard them…. our neighboring girls have said that…. after 5 O'clock…. After the lecture they are called to that science laboratory and then they will be beaten……

Moderator: But you have not heard them using the word 'Welikada'? Like the prison?

Person 1: No…. they maybe they have hidden it…..

Moderator: They are called to be beaten and they are slapped, how do you feel about that? How will that effect the person?

Person 1: That person will be humiliated…. In front of the others no?..... So, it's not good to slap in front of the others…. Also physical violence is bad sometimes…. The particular person will get some injury or some pain…. So it's not good…. It's sad….

Moderator: But the humiliation, to be slapped in front of the others, perhaps it's worse than the pain? What do you think?

Person 1: this will create person al vengeances between them….. because when they are getting beaten from their seniors they think… they want to beat their juniors….. that's what happens….

Several together….. it's a cycle…..

Person 2: …it won't stop…..

Person 3: …then only the heal from the pain….

Laughing…

Person 4: … exactly…..

Moderator: They heal from the pain, but there are other scars remain.

Person 1: The one who gets beaten now… will give that… to again….

Person 2: … the seniors have some…. Ahhh… some identity by having this beard and all…..

Laughing….

Person 2: ….. and these things… and having a motorbike's and three persons going on one bike…. And these heroism will make them happy…..

Everyone laughing…..

Person 2: …. And just look down on the first years….. small things…..

Moderator: We also noticed the beards, more in the south, we don't have so much beards. Now irrespective of whether you are Sinhalese, Tamil or Muslim, everyone has grown a beard when they come to the 3rd year.

Several together…… yeah….yeah….

Person 1: ..yes…

Laughing….

Moderator: I have felt like asking the boys, if it a thing to do in the Jaffna University? We usually don't see so many beards.

Person 1: 3rd year they have to have a beard…..

Person 2: Even, our boy has a beard….. the 3rd year boy….. because he's senior…

Everyone laughing…

Person 2:…. That is compulsory for them…… to differentiate from the others….

Person 3: … because they are not allowed to have beards in the first years….. and second year… that's why…..

Person 4: So they are using the chance to the maximum…..

Person 5: ……during our first year…… our batchmates… boys beg us…. Please take us with you…. If we come with you…. We will escape from ragging….

Person 2: …. They won't rag….

Moderator: If they come with you, the seniors will not tough them?

Person 1: No….. no…..

Laughing……

Moderator: So, they have some respect for you and because your teachers.

Laughing….

Moderator: when you were 2st year's and were asked to wear all these dresses, how did you feel? Did it make you feel uncomfortable? "Why are we dressing like small children?", when we are adults or have to wear all of this (Abaya) when you are not used to wearing these things.

Person 1: Exactly….. giggling….

Several laughing…..

Person 1: ….. I already said this…. that we haven't worn this one (abaya)….. so in this university it is compulsory to use this… and so…. What to do…. But there is also a distinction also… we have use it only for the 1.5 years…. 2nd year, 2nd semester….. after that… we can use any colorful shawls or anything like that….

Moderator: Ah ok. But it must be also very hot to wear it in this heat.

Person 1: even we have to get used…. To wearing socks also…

Everyone laughing….

Person 1: If there is no socks on the leg…. They will ask…. “where is your socks?” like that…. seniors will notice these things…. they will notice the socks also…. They will ask us… even if we can’t wear the socks… we have to give medical for them… even not for the lectures but we have to give them…. a letter that doctor has asked us not to wear socks….. because it’s hot… like that…..

Laughing…

Person 1: ….we have to take…. Get rid of them exactly….

Moderator: Do you have to wear gloves?

Person 1: gloves.. no…

Person 1: …. No we don’t have to…..

Moderator: What if your parents say, no my child should not be in the hot sun with all of this (Muslim attire), that she should wear a light color, can you do it?

Person 1: Sometime maybe….. before… not in front of others….. behind us…. Our seniors might talk to our parents, that they should wear like this…. so then they…. Might accept this… because on the registration data, we are talking our parents with us…. So on that day, we have a meeting…. So maybe they are talking with them then…..

Moderator: So, they tell the parents and then the parents get happy?

Several together….. yeah… yeah….

Laughing…..

Person 1: We are preventing…. They will be taking care of you….so, like that…..

Moderator: They must be talking very nicely?

Several people together….. yeah…. Yes….

Moderator: Otherwise it’s not very nice to wear bathroom slippers and walk around. As university students you are the future.

Person 1: When they come in the bus… people will laugh at them…. with the ‘bata’ and 2 plaits….

Person 2: ….they ask us… if we are working in the bathroom …. Like bathroom cleaners…..

Laughing…..

Moderator: That's really bad. So, during orientation week you have the introductory course, do you all have to go?

Several together….Yes…. yeah….

Moderator: Is that program good? Does it help to reduce ragging?

Person 1: Yes…. They…

Person 2: …They informed us…. There marshal and other student counselors…. And they provided us…. The telephone… mobile numbers also… if you face any problem…. Or any issues…. Then we can contact them…..

Person 2: …..in the evening in the orientation program…. The last day they call….. I forgot that name…. the seniors will come inside and they will rag us…. They are waiting for that day….. exactly….

Moderator: They are doing this even at the orientation?

Person 1: Yes…. But if the lecturers come, they will just run away from there…..

Moderator: So, most of the time there are no lecturers at that function?

Person 1: Yeah….. they will be gone actually…. They will just give the time for the seniors…. Especially it's for the… 3rd year…. Not the 4th year…. If the 3rd year come and slap even for us… the 4th year will come and prevent this… not to do like this and…

Person 2: …yeah….

Moderator: So the 3rd years are the ones that come to….

Person 1:…. Rag us…. Actually, they are the people waiting for the ragging….

Person 2: …. To do the ragging for the 1st years….

Moderator: Because they suffered 2 years?

Everyone laughing….. yeah…..

Person 1: …..total revenge….

Moderator: As second years, don't you have a little chance even to rag?

Several together…. No….. no….

Everyone laughing…..

Person 1: …..they are also under control of the seniors….

Moderator: We heard that when the 2nd year's became 3rd years, they had a huge party. Is that true?

Everyone laughing….. yes….

Person 1: we will have a huge 'pooja' in a 'kovil'…… and eat 'pongal'….. it's a very big victory for us…..

Everyone laughing…..

Person 2: …. Finally……

Person 3: …. They have been given….. seniority it seems….. so they celebrate….

Person 4: ..they come to the power….

Everyone laughing…..

Person 1: we actually….. learnt if from your batch…. The 3rd year… that is what you people did in the last year…. Right?

Several people…. (unclear)….

Moderator: Do you know what the ice cream party is? I think it's in the Management faculty. They have printed rules. Does the Arts faculty have anything like that?

Several together….. No…. no….

Person 1: ….only small things we are having…..

Moderator: They have to wear special clothes and if they are going to the ground tjye have to wear a Mud kit.

Person 1: …..there is no mud kit…. There is a Mud day!

Several people agree….

Person 2: they have an ice cream party….

Person 3: …..they have the mud….. what is it called?

Several together…. 'ponding'….

Person 3: ….Ponding day….

Moderator: what happens on the ponding day?

Person 1: They will be….. in a pond and….. there is mud…mud…. Yeah…

Person 2: …..the previous day they will dig the pond and….. they will pour water there….. and get ready for that…

Person 3: ….pour water into it…. Like a paddy field….. it will be like a paddy field…. They will take the thing and…. They will throw on each and every student and they will make…. Fun…..

Person 4: ….like take bath….

Person 5:….. pour water….

Person 1: …. They will take a bucket and…. Spray it to others…..

Several together….. from upstairs also…..

Everyone laughing….

Person 3: …..all the girls and….. everybody…. So you'll be wet….

Moderator: This happens in Arts faculty or all the faculties?
Several…..Arts….

Person 1: …. Only Arts and Management…. Management also have…..

Moderator: So, they have these at different times? So on ponding day, if you go towards the Arts faculty, it doesn't matter if your management you will get…..

Person 1:…. No …..they will ask and do….

Person 2: They will ask which faculty you are…..

Moderator: Oh ok, they will ask and do it.

Several laughing…..yeah…

Person 1: …They know that….. if it's management they have… other type of costume….. for the management…

Moderator: How can you differentiate the costume?

Person 1: In the Arts…… I know that…. for the first year….. boys specially….. they do not wear… they cannot wear belt……

Person 2: …… the Arts faculty….. it's the same….. they cannot wear…..

Person 1: …..for the first year they cannot wear belt….. they just have to… they cannot iron the shirt….

Several…… yeah….

Person 1: ….. just official trouser and this one…….

Person 2: …..only 'bata'……

Person 1: ….for the 2nd year 1st semester, they will wear Black color belt….. (laughing)….. they can wear shoes…..

Person 3: …..and light colored shirts…..

Person 2: ……official wear…. Ok…..

Person 1: …..for the 2nd semester they will wear….. colored belts….

Everyone laughing….

Person 4: …..in the 3rd year….. they can wear any shirt…… 'Bata' and anything they want…..

Person 5: ….. Whatever what you like…..

Moderator: We see them walking into the university with 'bata' and t-shirts.

Person 1: they are free from anything….

Person 2: …..if you see official wear shirt with color belt…. With black color trouser…. With slippers…. That is 2nd year 2nd semester….

Everyone laughing……

Moderator: Then you don't have to wear shoes also? You can wear slippers?

Person 1: Sandals…..

Person 2: 2nd semester they do not have to wear shoes…..

Moderator: But they still need to wear formal trousers?

Several together….. yeah…. Formal trousers….

Moderator: What about the girls?

Person 1: Girls can wear anything….

Person 2: …. 2nd year girls can't wear 'Shalwar'…. isn't it?...... 3rd year only can wear shalwar…..

Person 3: …. We can wear shalwar….

Person 2: … in the 2nd year?

Person 4: …… We can't wear jeans and t-shirt……

Moderator: in the 3rd and 4th year it's allowed?

Several together….. it's allowed….

Person 1: … 2nd year's…. is prohibited…..

Person 2: …. You're not allowed….

Moderator: So, no jeans in 1st and 2nd year. If a girl is wearing jeans then they are 3rd or 4th year.

Person 1: Muslims also have a distinction…. In the 1st year you have to wear only this one (shows a style of the head scarf) …. In the 2nd year only this one (shows a style of the head scarf)….

Person 2: ….. there is a different style….

Person 1: … there is a different style…. And in the 3rd year we can wrap it (head scarf)…. 3rd and 4th year…..

Moderator: Can you change the color?

Person 1: …..can change in the….

Person 2: ….in the 3rd year and 4th year we can use color…..

Person 3: ….. 3rd and 4th…..

Moderator: 1st year it's a straight one (head scarf), 2nd one is a little wrapped….

Person 1: ….it's a little styled one…. And the 3rd year the more styled one….

Laughing…..

Person 1: …..they can use colorful abayas and shawls….. and all…..

Moderator: So, you have to plan your clothes from the beginning. And each and every time you can distinguish batches. So it's an easy target for the ragging.

Person 1: yes…..

Person 2: ……easy recognition…..

Moderator: So, you have access to rag at any time. Do they have anything else like 'ponding' or 'bonding'?

Several people together…. "P…..Ponding…. ponding…. Pond….

Moderator: Do they have anything else like this?

Person 1: they spray some colored thing also…. Isn't it one day…

Person 2: …. This ink…..

Person 3: … blue color and all…

Moderator: What is that day called?

Person 1: there is another name they say….
Moderator: Is it 'Holi' (Indian festival of colors)?

Several together…… no its not holi….

Person 1: Not holi…. These colored things…. ink and all this they spray….

Moderator: Permeant ink?

Person 1: No….. coloring…

Several whispering among themselves… (inaudible)….

Person 2: …. In the management…

Person 3: In the management…… they have been color…. They will come to the bathroom and….. destroy it…

Person 4: …. They destroy the bathrooms and lot of things….

Moderator: Which faculty do you think has the most amount of ragging? Which is the worst?

Person 1: Science….

Person 2: ….. Not exactly Arts faculty…

Several together…. Science faculty….

Moderator: Why do you think the science faculty is the worst? There must be some reason you are saying science is worse. They hit the boys more or?

Person 1: There is no….. even in the Arts faculty to compare with that…. in the Arts faculty …. they will have distinction to identify the girls…. The girls shouldn't do this one…. For the girls… for the boys.. we can slap or do anything… But in the science faculty I have heard there is no distinction… even for the girls they will slap…. I have heard…. One of my friend is being slapped by her senior….

Moderator: Girl or boy?

Person 1: Girl… in the hostel she does… even for the Muslims and Sinhalese there is no distinction….. they are asked to read and…. Even if they have lectures after the lectures for one hour they have to write…… the card…. Birthday card or something like that….. for their birthday wishes, a group of people has to come into the room and sing birthday wishes….. for their seniors…. In one room they have to arrange and…. It is also through the normal calls …. They cannot use WhatsApp calls….. to contact with them….. they cannot use WhatsApp.

Moderator: They have to make a birthday wish, they have to sing, and do everything from the phone?
Person 1: and they have to sing also….. they have to sing and I have heard…. That science faculty students have to come… and….and especially they have to….. another thing with the science faculty is they will be ragged by the 2nd year….. not by the 3rd year or 4th year…. 2nd years only rag the first years…. Because they are the hot people…. Who had the ragging and all….

Person 2: ….so, that amount will be more increased….

Person 1: ….And I have heard that they are asked to come to the home and…… do all the ragging things…. that means some kind of… hmmm…. They have to…..

Person 2: They have to dance, they have to sing….

Person 1: …… They have to change…..

Moderator: do they ask them to take their clothes off, have you heard like that…

Person 1: no…not like that ….. they have to sing….. they have to change the Sinhala song into Tamil and the Tamil song into Sinhala…. Like that……

Moderator: Have you heard anything else about the science faculty?

Silence…..

Moderator: (P) How many students are in the science faculty? (A) They might not know.

Moderator: Have you been identified as anti-raggers because you are the English group? Or do you feel more isolated because everybody thinks, oh! English students?

Several laughing….. speaking together…. Unclear…

Person 1: Some people respect and…. In some cases…. They say "who are they, like that?"…….

Laughing…..

Person 2: …. " they are English"…..

Person 3: But there is another thing also…. If they get to know we are English…. They will give us to translate all the stuff…..

Everyone laughing….

Person 3: …. So, if they ask us…. The subject what we are doing….. we will say Geography or something like that….

Everyone laughing…..
Person 4: ….we don't say we are doing literature……

Moderator: Because they try to give all the English work?

Person 1: English translation…. That is not only…. One work…. There is a huge papers and all they will give us…. We will be exactly caught in the hostel by seniors….. so we cannot get rid of them ….exactly…..

Moderator: I thought sometimes because you learn English they will either be frightened of you because of the English or you will get ragged more because you study English?

Several together….. No……

Person 1: … Because we are not identified….. even now we are 2nd years but….. but we are not connected with our batch students also….. that's a big problem…..we feel like we are isolated….. we are 3 in our batch….. the 3 of us come here…. To the university and have our lunch and….. breakfast together… we sit, we study, we read our novels, and get back home…..

Everyone laughing….

Person 2: we have limited numbers…..

Person 3: ….. very limited…..

Person 4: …. A very secluded group….

Moderator: That's why all of you are so close. But do you feel it's a bit of a problem, when the rest of your batch is not, when you don't have a connection. Do you get invited for these sports events and parties and, or not so much?

Person 1 : They invite….

Moderator: So, it's not a problem?

Person 1: No……

Moderator: So, you don't feel isolated and your included in everything?

Person 1: yeah…..

Moderator: this is just for my knowledge, did you go for, did you have your welcome party?

Person 1: Not yet….

Moderator: Do the Muslim students go for the welcome party or not?

Person 1: I have not heard so….. but we have a separate welcome party… in our Muslim Masjid….. so it will be done by second years….. so the next month we will be having our welcome party…..

Moderator: So, you have a separate Muslim welcome party. But the other welcome parties…..

Person 1: the University welcome party….. Muslims will go……

Moderator: So, the Sinhalese have their welcome party and the Tamils have their party and the Muslims have their separate party? Or it's one party?

Several together…. Faculties… faculty……

Person 1: …. Faculty wise…..

Moderator: but the Muslim boys must be going?

Person 1: Boys….. for the party they will go….. but for other things they will not go……

Everyone laughing……

Moderator: Have you heard where these boys are drinking in the hostel, you said before that they were drunk, in which corridors? University corridors?

Person 1: On the steps…. Like…. But now we don't see… like that…

Moderator: Student unions, how do you join? are there elections?

Several together…. There are general elections…..

Moderator: Someone told us that you have to be from one school, if you want to be the president of one of these associations. Is it like that?

Person 1: We don't know……

Person 2: ….by voting only…

Moderator: Are there girls as student union presidents?

Person 1: Secretary, treasurer…..

Several agreeing…..

Moderator: But there are no girls as presidents?

Person 1: Yeah….. they won't allow…. (laughing)…. Boys won't allow….

Laughing….

Moderator: Do you know why that is? Or they want to keep the control?

Person 1: Normally they have that notion….. male domination…

Person 2: …. Girls also don't like to go….

Person 3:…. Yeah….

Several talking together…… (unclear)….

Person 4: …. Girls don't want to get involved in political issues….

Person 1: …..they only want to study and get the benefits….

Moderator: So, girls are the only people who study well and they aren't interested in politics…..

Person 1: University… students president…..is involved in something related to political issues…. So, girls…..

Moderator: So, most of the time it's some political party….

Person 1: …..there is some political party influence

Moderator: Do you mean that there are political parties involved in the faculties union, you have the main student union and then you have every faculty isn't it?

Person 1: As a student's union…. The president, secretary and the members have to involve some political issues around Jaffna and….. related to our ethnic problem….. and other things…. yeah….. and reports and other things…. they have to expose themselves…..

Person 2: …. They are the link people….. they are the representatives…

Person 1: …so it may cause some problems…. So girls generally don't want to…. Interfere…

Moderator: Perhaps women are not so interested to be involved.

Person 1: Yeah…

Moderator: So, what recommendations can you give for other students, from other faculties, to respect each other at home and at university, to decrease ragging. What can you give as recommendations, what do you think can recommend?

Person 1: I think the ragging…. What they are telling all these time.. that is in away good… to know each other and to have distinction between 1st year and 2nd year…. These things are ok…… I think….. but when it comes to the physical abuse, only it is, the bad thing… otherwise they should have some interaction…. Without having the word ragging…..

Person 2: … anything if it is not…. Affecting the mental health or the physical health…. It is good…. Because it will be a good memory… or good moments in their university life… sometimes….

Moderator: What do you all think?

Person 1: The same.

Moderator: What advice can you give, like have more societies or clubs or, sometimes people say that the students have too much free time, what do you think?

Person 1: They have much energy…. So we have to engage them in various activities…..

Person 2: ….yes… must engage them in activities….

Person 1: So… we have to activate the clubs and societies and the….. we have to make links with the society…. So then we have to…. Make a room for the connection with society… and other opportunities….. then it will be of more benefit to their future…. And they….. they…… they make their time also very useful….

Person 2: … it will help them to….. to….. make their…. Leadership qualities and improve their…. Maturity and all these things……

Moderator: Are there any societies in the university? What societies are there?

Person 1: There is a literary society…. And other…..

Person 2: … associations are there…..

Person 3: ….. clubs….

Moderator: What are they going in these clubs….

Person 1: So cultural events….. they conduct each and every year….. cultural events…. And sports… they will have some….. arts…. It's called Arts week and Science week…. That time they will have a one week program… some sports activities…. Cultural events…. Dramas and …. To get them involved….

Person 2: …. But…. My opinion is that we have to make….. we have to give opportunities to link with the society…. Not inside the university….. outside the university…..

Moderator: Activities that occur outside.
Person 1: Yeah outside….

Moderator: Because before you had a lot of social work, community work…

Person 1: …yeah….. it's …it's… it maybe an awareness program or….. something…… evening schools…. It should be planned….. well planned and…. We have to engage these youngsters…..

Moderator: Because you can have these evening classes where you can help these students from secondary school or what so ever. To engage and occupy them.

Person 1: Remote area students face many problems….. seeking teachers…. The proper teachers….. so these type of students will… if we… if we have the links… they can make it…. The students also get benefits from school children….

Moderator: What do you all think? Any ideas?

Person 1: According to me it is ok…. We didn't have much harsh ragging….. my point of view is that ragging is good…. When they come to 3rd year or 4th year they can overcome this stress…. Struggles and obstacles….. if they did not face anything like that in the first year…. They cannot do (manage) do it in the 3rd year or 4th year…..

Moderator: Then the bad types of ragging?

Person 1: I didn't mean that physical abuses or something like that…. but they can make something like this small ragging….

Moderator: So, who should control that it is kept nice? If you say ragging can be nice and good, who should control it? That you don't cross that border?

Person 1: 3rd year and 4th year….

Person 2: …students…. Student union…..

Moderator: But you say they are politized, perhaps they are not so interested?

Laughing…..

Person 1: But they can take steps to minimize this….

Moderator: So, that means that perhaps if we have the right people in the union?

Person 1: only they can minimize these things…. only they have to bring a rule that….. our batch should not do ragging…. And should take steps to minimize that….

Moderator: So, they can be the driving force. So, that we keep it a good, decent joyful way and you don't cross the border where it suddenly, gets messy.
Person 1: They can sit at the back…. But they don't do that…. that's the problem….. student unions….

Moderator: You also have the staff at the university, you have some rules and regulations, who controls that you are following these rules?

Silence…..

Moderator: There are rules and regulations, that you shall not rag.

Person 1: According to our university rules… that we should not rag… rag our juniors but….. none of the staff will come and check…. Whether we are doing this ragging or not… whether we are ragging the juniors or not…. So we have to understand the fact that we should not rag or give physical punishment to the juniors…. So we should have a very good relationship with them….. we should have a set up on how we should interact with them…..

Moderator: But you should also have a control from higher up.

Person 1: Not a control…. Like a boundary…. We should have…

Person 2: …..like rules….

Person 1: … we should have small interaction… not a very harsh treatment towards the juniors but a very… like a …. Very….

Person 2: …..friendly talk… interaction….

Person 1: ..yes… way to talk…. Like…only that thing is welcome…..

Moderator: Are there any services that the students might need that are not here. Because if it says, there are sort of these not very good type of ragging, more severe ragging, would there be any services for those people that need some help? Would you see any services for you like, for example, senior counselors? Also in other places, we have counselors outside in the hospital, we can have other people outside the university, would that be something needed? Is that necessary?

Person 1: there are some counselors….. and student counselors but if the students really need some help they can go but…. I think that…. none of them will go…. I think….

Person 2: …. They are just labeled as student counselors….

Person 1: …..Just called student counselors…..

Moderator: I know but do you think it's enough with that kind of service?

Several together…. No….

Moderator: They are just lecturers, they are not trained counselors, so do you think it's better to have trained counselors?

Person 1: Yeah…..

Moderator: not only for ragging but also if your stressed.

Person 1: yeah…. Yeah….. students need a counselor….

Person 2:….. during the final year… the students feel a little bit scared about their future…. The next year what will happen…. The opportunities…. They have to face the future…. From the next year….. so that time… they… They need… counseling….

Moderator: So, someone with whom they can discuss.

Person 1: Professional counselors….

Moderator: That is what I mean. That is not involved in the teaching or training but you can discuss just those stressful things.

Moderator: Thank you very much!

FGD 3

Moderator: Introduction

Moderator: What is ragging to you? What do people say ragging is or what come to your mind when you mention ragging?

Some whispering between the participants.

Person 1: Hmm…… Physically harming or mentally harming?

Moderator: What do you think?

Person 1: Hmm…Can only complain it's ragging if it goes to the physical level.

Moderator: What do the others opinions? Do you agree or disagree?

Speaks very softly and inaudible …….

Person 2: (speaks softly) Physically or mentally harassing another person is ragging

Moderator: What do the others think, what are your thoughts, what to your parents say? You must be having some idea as university students…..

Person 3: If someone is going something that causes physical or mental harm to another person

Inaudible whispering

Moderator: Is it done with or without consent?

Everyone together: without consent

Moderator: Is ragging always bad or there also good parts?

Person 1: Sometimes can be good (others agreeing with her and talking at the same time), sometimes can be very bad (another person talking at the same time)

Person 4: If you say ragging………

several talking ppl at once

Person 4: oh I don't know…… giggles
Person 1: according to the action taken……… giggles

Moderator: please feel free to express your ideas, there's no right or wrong answer

Person 1: Sometimes ragging is like an orientation…… to get to know people….. it's good like that…… to make connections….. but after you make the connections…. If the seniors enforce….. like to get their work done……. Like trying to ask for favors every time……..If they have a project they hand it over to us, which is a real nuisance.

Moderator: Does ragging continue even after the orientation?

Everyone agrees

Person 2: Sometimes yes……….inaudible (several speaking at the same time)

Person: Some people…….. inaudible (several speaking at the same time)

Person 1: Not violence per say……. But says verbally don't do this…… you can't do this yet….. can't go to study halls… like things

Moderator: I have heard that there are some written rules to be followed, I don't know if it applies to the medical faculty. Is there something like that?

Person 1: In the beginning there is a way to talk……..

Others agree

Moderator: What about the dress code?

Everyone talking together

Person 2: Other faculties, some universities have it…… (two people talking together)

Person 1: Here the management, Arts faculties have it (others agreeing)…….. our medical faculty doesn't have it….. As far as I know none of the medical faculties in Sri Lanka make students wear "Cheetha" dresses. (another person agrees)

Moderator: What else is there in ragging? In other faculties, in other universities? I have heard different things…..Like troubling girls?

Person 3: There are some things……. Like personally….. they select a girl and call it ragging and harass her unnecessarily.

Moderator: Have you heard of anything like that happening here?

Person 4: Here we haven't heard anything like that (others agree)

Person 5: we also only heard thing from the news

Moderator: So not in the medical faculty, but what about other faculties?

Everyone agrees

Everyone talking softly together…… management faculty

Person 1: There is a little bit in the management faculty………inaudible

Moderator: What about other faculties?

Everyone mumbling together

Person 1: we had the most connections with the management faculty…… we shared the hostel with the girls from Management faculty……

Person 3: In the hostel…….

Person 4: Yes….. we were in the hostel with them

Person 1: their seniors used to come in the morning…….. have to walk in line…….

Person 2 : Seniors come to the hostels…….

Person: Science…….. seniors

Everyone mumbling…… comes to the hostels

Moderator: Who are these seniors? Girls or boys?

Person 1: Boys for the boys and girls for girls

Moderator: Do the boy harass girls and girls harass boys?

Person 5: We haven't seen

Person 1: No….. we haven't seen…. Have we?

Others…. No we haven't

Person 1: they talk but……

Person 2: yes.. they talk

Person 1: They things till the girls cry sometimes……. But it's only verbal……I don't think they do anything more than that

Moderator: so you haven't been ragged by boys?

Mumbling…….. no

Moderator: So only girls for girls?

Several people talking together

Person 2: No…….. it's not like that…..

Person 4: During the orientation……..

Person 1 : there is no rule like that……..

Person 4: Talk during the orientation (person 3 talking at the same time)……but it's not ragging

Several people talking together……. It's not a rag….. asking the name, where your from….

giggling

Person 3: Things like that…..

Person 1: They just talk in the beginning…… to sing……….

Person 6: things like that

Moderator: but nothing harmful? Do students from other faculties harass you?

Everyone…….no

Person 1: No……. not from other faculties….not to us

Others giggle

Moderator: so your lucky your from the medical faculty

Everyone giggles and agrees

Person 4: That's true……. And our faculty is also separate….. away from the main university

Moderator: Where does ragging occur mostly? Inside the faculty or outside the faculty or at the play grounds or where?

Everyone together…….the hostel

Person 2: The grounds
Person 1: Hotel…….. it happens….. during weekends and all

Everyone talking together inaudible

Person 3: different from faculty to faculty…… happens in the hostels and the grounds

Person 4 : Management ones are taken to the seniors boarding's and they do it…….some say

Moderator: That means places outside the campus?

Person 4: There are seniors who live outside

Moderator: So they can't take a whole batch……..?

Several talking together……take several students……. Select a few

Moderator: What are the times these things occur? Are there specific times?

Everyone giggles….. No not really…….

Person 1: For the females…….of course……………… during afternoon's……. when the students come to campus……. Don't come to the hostel….. don't do a lot for the girls

Moderator: the senior girls have not come to the hostel and told you anything?

Person 1: No not to us……..never tell us anything directly…… comes from the "aiyas" (senior brothers)…….. the senior girls tell the senior boys, and they tell the boys from their batch…. And they tell us…..

Everyone giggling

Moderator? From other faculties?

Person 2: no……in our faculty……..

Person 3: not a rag…….

Person 6: the seniority……..

Person 5: make a small interference……..

Moderator: I didn't understand…… explain it to me

Everyone giggling

Person 3: if they want us to do something…….

Person 4: if they want to get something done….. it will go to their batch…… then it will come to us across their batch…

Person 2: the senior girls message will go to the boys of our batch…….

Person 3: Now……..

Person 1: Even if the senior girls are face to face with us……. The won't tell us directly…. Even if it's some mistake we did to them….. for example, like closing the door in the study hall on them……..or was in the study hall…… or something….. it will definitely first go to the boys and then come to us

Everyone giggles

Moderator: Does this happen in other faculties also?

Person 1: I don't think so……..don't think it happens in Jaffna……..

Person 2: The girls………..

Person 1: The science faculty…….. we don't know…… But in our faculty don't say anything directly.

Moderator: Is it only the seniors who rag, or do the others like demonstrators also rag?

Person 3: No no……..nobody else…..

Moderator: Is it only the immediate senior batch or do seniors from other batches also rag you?

Everyone together………all of them

Person 4: I think the largest involvement is from the most senior or 4th year batch……final year

Everyone giggling

Moderator: I have heard that no matter how senior you become the batch above them will harass them…… or there are designated places for students from each year….

Person 1:……… not seating………..

Person 3: No nothing like that

Several speaking together ………. But the study hall seats a prioritized for the seniors

Person 2: senior priority must always be given…….
Person 3: Even in the cafeteria

Person 1: we are not allowed into the study hall……..giggles……

Moderator: I have heard that in some faculties, they are not even allowed to go to the bathroom…… Is that true?

Everyone together……. No…..no……nothing like that

Person 4: No there's nothing like that in Jaffna I think……..

Everyone together: Nothing like that

Moderator: Not even in other faculties? Don't allow to go to the bathroom and don't allow to go to change their pads.

Person 1: Nothing like that

Person 5: No

Person 1: We were in the kokuvil hostel with Arts students……….there are a little different ………but there was nothing like that

Person 6: there were no first year's there

Person 1: there were only 3rd years and 4th years

Person 2: They were seniors

Person 3: We didn't have a problem with them

Person 4: I don't think they caused problems for their juniors also

Moderator: Is there a group of students who is more vulnerable to ragging?

Person 3: No

Moderator: Due to their ethnicity, religion, economic state or caste?

Lot of people speaking together: No

Person 4: Not because of race or religion but can be due to their behavior

Person 1: agrees

Moderator: So never because of ethnicity or caste?

Person 2: No, no

Everyone agrees

Moderator: is this only in the medical faculty or in all faculties?

Person 5: No in it's the same in all faculties

Person 6: agrees

Person 1: Even if they rag it's done separately…..

Person 4: Sinhalese are ragged by Sinhalese seniors

Moderator: I have heard that Tamil seniors sometimes rag Sinhalese juniors…….is that true?

Few people talking at the same time; this happens at the orientation…….they just talk

Person 1: they don't call again and do anything….. just the first chance

Moderator: What do they ask?

Person 5: just about ourselves

Person 6: Like our seniors they also ask us information about ourselves

Person 1: They don't give us work or anything like that…… just ask questions

Moderator: when someone is getting ragged in an unusual way does anyone stop it?

Person 3: What do you mean?

Moderator if some is getting ragged unpleasantly, does anyone stop it? Or they mind their own business?

Person 1: No……..isn't it……

Person 3: Even during our orientation our pre-clinical coordinator is there to make sure nothing happens.

Moderator: in other cases? If someone is abusing a junior physically or something like that?

Person 2: A senior above the person who is ragging the junior will stop it. But the other juniors can't stop it.

Moderator: Who people complain to anyone? For example, like counselors, wardens, marshals, deans, lecturers?

Person 1: yes, they complain

Person 4: there was a problem in our junior batch and they complained to the dean.

Several talking together…….. they brought the wardens……. (not clear) and asked them……

Moderator: So, there are time that students complain to someone.

Moderator: What did people say when they heard your coming to the Jaffna university? What did your parents say? Were they afraid to send you? Do they say it's a safe place?

All together: (smiling) …… It's a safe place

Moderator: What do they say about ragging? That it's less or more? For example, less or more than Peradeniya?

All of them giggling ………. (not clear)…….. it's less here…… Peradeniya is more …..

Moderator: Do they say ragging is less in Jaffna University?

All giggling

Person 2: Less

Person 6: Say there isn't

Person 5: very less

Everyone agreeing

Person 4: Medical faculty has no ragging

Person 3: medical faculty doesn't

Person 1: Less in the whole of Jaffna university

Person 5: They call us "aala" (person who doesn't like ragging)

Everyone agreeing and giggling

Person: Jaffna university has less ragging

Moderator: So, the whole Jaffna university is "aala"?!

Everyone giggling and agreeing

Moderator: What do they say about the education at Jaffna University?

Several people talking together: they say the education is good

Giggling and talking together….. (can't understand)

Person 2: say it's too good…….. giggling

Moderator: Why is that? Why do they say that? Because you can't understand?

Everyone giggling

Person 2: No it's not like that

Person 3: They give the main priority for studies

Moderator: Oh, so they focus a lot on studies and not on other aspects…

Everyone agrees

Person 4: There are other things not there isn't, but prioritize education

Person 6: But priority is always for education

Moderator: So you don't have sports and other activities?

Giggling

Person 2: There is…….

Person 5: Only after lectures, can't cut lectures and in the evenings………

Person 1: Even the "Medicos week" continued for a month because the events were only after lectures in the evening……. Giggling

Moderator: So the students and the lectures focus only on studies?

Everyone giggles

Person 1: No the lecturers are the ones, students don't mind doing sports

Moderator: Since you come from far places, do you go home for weekends? Or is it difficult to go home for weekends?

Several talking together………it's difficult to go home
Person 2: Can only go during vacations

Person 3: Can't cut the lectures

Person 4: Can only finish the lectures and go home……

Several talking together and inaudible

Person 5: Or must finish lectures and go on a Friday, and stay Friday and Saturday and come back on Sunday evening……

Person 6: Otherwise must cut lectures and go …….and then you get punished….

Everyone giggling

Moderator: Are all lectures compulsory?

Person 2: They say all lectures are not compulsory

Everyone speaking and giggling together

Person 3: Lecturers get angry……

Person 1: They get angry……. And then don’t give the tutorials…. only give to the students who came for the lectures…..

Person 4: You need 80% attendance for the tutorials

Moderator: Ultimately everyone attends irrespective of it being compulsory. Does this only happen in the medical faculty?

Person 1: only medical faculty

Person 3: No right?........

Everyone speaking among themselves (inaudible)

Several talking: not all lectures are compulsory

Moderator: So, the students from other faculties have more free time?

Person 2: Yes

Person 4: They have enough free time

Everyone agreeing

Giggling

Moderator: What do they do in their free time? If they don’t have much studies and don’t go home also, what do they do?

Several people talking together……. (inaudible)

Person 3: They stay in the hostel and sleep……giggling

Moderator: Do you think ragging is more because the students from other faculties have more free time?

Everyone agrees

Several people talking together……inaudible…….our faculty…..

Person 1: We don’t have time to meet the other batches….

Person 4: Most of the time we are in lecture halls

Person 1: the seniors are also at their lectures……. They do their clinicals in the morning and when they come for lectures…….. we’re finishing our lectures and going for practical’s

Person 3: Then the we don’t meet each other

Moderator: Have you heard of any severe forms of ragging occurring here?

Person 2: I have heard of severe forms but not in the Jaffna university

Moderator: What have you heard?

Everyone giggling

Moderator: What have you heard? I'm asking you because I don't know and want to know the things you have heard.

Person 2: I have heard things in the news……. That in some universities……. They even take off clothes….. I've heard like that……

Moderator: Have you ever heard of things like that here?

Person 2:…….No…… some students………get stressed and go to the extent of getting depressed……..I've heard things like that…..not here

Moderator: Ive heard that some students want to give up their studies and leave, haven't you heard of incidents like that?

Person 3: Nursing……

Person 4: I think there are things like that in nursing…….

Moderator: What happens at Nursing (faculty)?

Person 4: Because the ragging is severe, 2 students from my area gave up studies here and went to Nursing school…….they came here to do nursing but because the ragging was too much they left….

Moderator: What sort of ragging was it? Were they abused physically or were they asked to take their clothes off?

Person 4: I think one of the boys were beaten……… not a lot but very frequently….. But I don't know much about the girl

Moderator: Do you think the boys are ragged more than the girls?

Several ……. More for the boys

Moderator: Do they get beaten?

Several together…….. not clear

Person 1: they just touch us but not physical with us (girls)

Person 2: I think they get beaten

Person 4: yes they get beaten…. Why we saw them getting beaten while going for (not clear)…….

Everyone giggling and agreeing

Moderator: Which faculty was this?

Everyone together……. The Science faculty

Person 3: Slap them, ask them to kneel and things like that…..

Person 5: things like that

Moderator: I have heard of very severe forms of ragging, are there thing like that?

Person 1: There are instances where students have died……. Died while they were being ragged…..

Moderator: Here (Jaffna University)?

Person 1: No…..no…..

Others giggling

Moderator: Where did that happen?

Several at once…….Peradeniya

Person 2: committing suicide

Several talking together….. (inaudible)

Person 4: Not too long ago……wasn't it?

Person 1: Inaudible

Moderator: What happened for them to die?

Person 1: they were physically abused…… hit or abused….like putting water and other things……

Moderator: So the Tamils don't rag the Sinhalese….right

Person 2: Only asks questions during the orientation

Person 1: They don't give us tasks or anything like that……..

Moderator: Is the ragging worse among the Tamil students?

Person 1: The Tamil students mostly come from their home (live at home)……so, ragging is less I think

Person 3: Yes…… ragging is less…….. they don't stay in the hostels and they only meet the seniors when they come to the faculty

Moderator: So you think that ragging is more among the Sinhalese?

Person 1: Yes……so…..

Person 3: Not ours……..

Person 1: Not that it is more……

Moderator: Not in the medical faculty but among the other's as a whole

Several people agreeing……. There is more among the Sinhalese
Person 2: Science faculty……has a lot

Person 1: They know you're in the hostels…… they don't come to your houses

Person 2: Agrees

Moderator: Does anything happen after the welcome party?

Person 4: No……nothing

Others agree

Moderator: In other faculties?

Person 4: No…… in other faculties the welcome party is only held after a year…….

Moderator: Why is it held after a year?

Person 3: They wait until the new batch arrives……

All the others agree

Person 3: they rag until the new batch comes

Several people say the above at the same time

Moderator: The ragging must be more severe in then because you won't be asking them to only sing for a whole year

Everyone gigges

Person 5: Nothing serious…… things like go and talk to a tree…

giggling

Person 1: They have the dress code for a year….. “Cheetha dresses” …….. there are thing like that…..

Moderator: So do these girls like to wear these “Cheetha dresses”?

Several people at once……probably not……giggling

Person 3: They have to plait their hair…….

Person 1: We saw wearing Kandyan saree……. Isn’t it……

Person 4: Plaiting their hair…… wearing bathroom slippers…..

Several talking at once…….

Moderator: Cant they complain? Can’t they say I don’t want to wear these dresses?

Several at once……. No they can’t say that

Giggling

Person 2: They will be called “aala” in the batch then…

Giggling

Several talking together

Person 3: Will be made an “aala” in the batch

Person 5: they will lose all connections with the batch

Person 6: They will not get any help from anyone or have connections with the seniors…..

Moderator: Are the “kuppi” (classes conducted by the seniors) compulsory?

Everyone together………..No……No

Person 3: Only if you want to attend

Moderator: Is that only in the medical faculty or in the other faculties also?

Person 1: No in all the faculties…..I think

Everyone together……all faculties

Person 2: Only if you want to go……..”Kuppi” are not compulsory

Moderator: Do you have to pay?

Everyone together……. No… No…..

Moderator: I have heard that you have to pay for these classes, and they are compulsory

Person 5: No, no you don't have to pay for these classes

Person 4: No you don't have to pay, they do it willingly……as a help to the students….

Few people agree and talk at the same time

Person 1: It's like something that has been continuing……..

Moderator: So it's a way of helping the juniors

Few people talking together

Person 3: It's like a continuing tradition

Moderator: I have heard that girls have to make food and go for boys…….

Giggling…….

Person 1: No……..

Several people giggling and talking ……inaudible

Person2: Not here, but it's there in other faculties……

Person 3: Tell to bring something…..

Moderator: What do you mean?

Person 3: Tell to bring something from home

Person 2: Tell us to bring something when we come back from home…

Several people together……..but there's no set rule that we must

Moderator: Have you heard that in the university, that students have been asked to bring things and they have problems because they can't afford to do it

Person3: I have heard that it happens in the "Rajarata" campus……

Moderator: Doesn't it happen here?

Several whispering……. Nooo………

Several whispering and giggling……..

Moderator: What did you say?

Giggling and muttering……..inaudible

Moderator: I heard that those thing happened here also

Person 1 & Person 2: Maybe in other faculties but not in ours…….

Person 2: Nobody asks us for food

Giggling & talking……..in audible

Moderator: Is there anything that you experienced that you didn't like?

Coughing………

Person 2: Didn't like………..

Whispering and giggling …..

Person 2: If a senior asks who they are and we don't know…….. they scold us a lot

Giggling

Moderator: Is that all?

Person 2: that's the thing we don't like…..

Moderator: Have they asked you to feed them or anything like that?

Person 3: No, No nothing like that……

Person 2: There had been before but not now……

Person 3: Not anymore

Person 2: Was there about 3-4……(inaudible)……….

Moderator: Do you think that ragging has decreased now?

Everyone agrees

Person 2: Almost disappeared now…..

Person 3: Yes…..

Person 1: It's getting less and less……much less than there was before

Moderator: You must be having older siblings…….when compared to them is it less?

Several talking together in a low voice…….in audible…….

Person 4: That's here…..isn't it…….

Person 5: Less here

Person 1: Very much less in our faculty

Person 3: In compression to before……almost nothing

Person 2: Just ask details about you……. that's all

Several muttering at once…….

Person 2: Those day……. that is…….. Not Harmful……

Moderator: So do you think this sort of ragging……asking questions and things is good?

Person 1: Yes……

Person 2: Otherwise we won't get a chance to get to know the seniors

Person 4: What we have is good….

Several agreeing

Person 5: What happens to us is good when you compare

Moderator: You have an introductory program during the orientation week; What's good in that? What did you like in it?

Several people talking together……..it's too much

Person 3: It's too long……

Person 2: Takes up too much time…….

Person 4: We did it but it's not very useful

Person 5: Wasn't useful but prolonged out time…..If someone came with hope of improving their English, it wasn't very helpful…….

Moderator: Does that mean that there were too many unnecessary things?

Several together……..No…… no

Person 2: Length was more than the substance…….

Several talking together……inaudible

Person 1: Have to pass this and the MBBS exam also…….

Person 4: Have to pass the English exam…….it feels like a waste of time because it's long

Moderator: What about the other lectures? Things like GBV, personal professional development (PPDS) …….Was there some lecture that was good?

Several…….. there were some

Person 1: Some were good……. inaudible…….

Person 2: They expect us to write everything in the exam……. that's the problem…….

Moderator: What do you mean?

Person 5: PPDS also has an exam……

Person 2: They expect us to write all the definitions and things in the same words they……so that's not good

Moderator: Does GBV also have an exam?

Several together…..Yes…..That comes under PPDS…..

Person 2: There is a big section called PPDS……. Then GBV is taught in that and these come in the exam…..

Several talking together……inaudible……

Person 2: There was a separate lecture on GBV also……. they taught about laws……… Someone from the law faculty came and did the lecture……

Moderator: Was it good?

Giggling

Person 2: the law lecture was too much……..we didn't understand anything

Giggling

Moderator: Is there something you think that should be added to the program? Do you think there is something that you will benefit from?

Person 2: It's better if it's done in a way that they don't expect an answer but in a more practical way….. better if it's done to give knowledge……

Moderator: Does the introductory program help to reduce ragging? Does it help you make better bonds with seniors?

Person 3: No not really……

Person 1: No in the orientation…….

Moderator: I mean in the introductory program

Person 2: The introductory program is a part of the orientation……..they make time for us to meet the senior batches……

Moderator: What do you mean?

Person 3: There is a hall like an examination hall……and they have like 3 hours to meet one batch that is senior to us…… this happens once a week to sort of get to know the seniors….

Others agreeing

Moderator: If you feel like it you go and talk to them?

Person 4: No they come and talk to us…..

Person 1: They prepare different programs

Person 2: No…. you cant ignore them…..

Person 1: they have different programs…..sports…..different things……

Moderator: Does ragging occur here?

Person 1: No it doesn't

Person 4: They ask us to sing, dance……do sports……

Person 2: Muslims……inaudible

Moderator: What happens to the Muslims?

Person 2: Nothing….. they can't participate in anything…..

Person 5: They never participate in anything

Person 4: Not in anything……parties, events nothing……

Moderator: Do they get isolated because of this?

Person 2: No they do it by choice……their seniors tell them not to…..and then they don't have any influence from the other races…..

Person 1: They remove themselves saying that the seniors told them not to……

Person 2: They don't come for sports meets……..not of anything…. Not for functions…….

Moderator: Their seniors tell them not to? What if there is a very social person who wants to participate?

Person 4: Don’t know what will happen to them….

Person 2: They don’t even have an item in the social….. girls cant but the boys have no problem……

Moderator: Don’t the Sinhalese tell the Muslims to do things?

Several together…..No no

Moderator: Do the Muslims come and say anything?

Several together……..No……

Person 2: It’s like they almost don’t come to the orientation

Moderator: so they are only told not to participate?

Person 2: Maybe they do something separately…..

Person 1: They stand on side and watch…….

Moderator: So it’s attendance compulsory for the Muslims?

Person 3: They just come and stand on a side and watch

Person 2: …….there is a person they was brought up like a Sinhalese person….here you have to go by the dress code…..you can only wear Black…….

Giggling

Person 2: She didn’t like it initially……. Now she has adjusted…… But the foreigner junior girl won’t listen to these things……

Moderator: You have a foreigner?

Person 2: A girl from Kuwait in our junior batch…….

Moderator: Does she become a target then?

Person 2: It’s difficult to rag her……

Giggling

Person 3: The seniors get ragged by her……..giggling

Giggling

Moderator: Give me an example?

Person 4: You cant argue with her……

Person 5: Even if the seniors say something she doesn't take notice of what they say……

Person 2: She's friendly with all the ethnicities…….. The rest of the Muslim girls follow the dress code, she's not like that. She modifies it the way she wants…… The others have a problem of getting her to follow……

Person 1: Once they had told her but she ignored it…….She had said…. "don't come to tell me anything, I have paid and come here"…….

Moderator: Can you pay and come to this university?

Person 2: Only because she's a foreigner

Moderator: Then is she considered as an anti-ragger?

Several together……. yes……

Moderator: Do they restrict her from activities?

Person 2: She's friendly with students from other ethnicities….

Moderator: So she wasn't told not to participate?

Several together……sure she was told…

Person 2: I'm Sure she was told not to several times……but she didn't listen

Moderator: I have heard you cant attend anything if your anti-ragger but how come she's not told not to attend?

Person 3: No they don't attend

Moderator: So she doesn't attend these functions

Person 2: No others from the batch

Person 4: She comes…..

Person 5: She didn't have an item at the orientation……

Person 2: Did she come for it?

Person 1: Yes she was there…..

Others agree

Person 2: During the orientation they ask boys and girls to stand together like couples…… the other Muslim girls don't come but she came……

Giggling…..

Moderator: Are the seniors angry with her for not listening to them?

Person 2: Must be angry

Moderator: So you are also her seniors, aren't you?

Person 1: We like it when she's there….

Giggling

Person 2: It's more of a problem for the other Muslims because she's active and doesn't do as she's told

Moderator: So the Sinhalese and Tamils don't do anything it's only the Muslims?

Everyone together…… yes

Moderator: How are the anti-raggers among you treated?

Person 1: Anti raggers don't stay in the hostel……..

Everyone giggling

Person 4: it's not that they can't they don't want to stay…..

Person 2: They don't stay…… they stay in boarding's from the start

Moderator: they won't stay or mix all the years?

Person 3: They stay in boarding houses

Person 5: they come for sports meets…….and participate

Person 2: They don't come for "Kuppi", parties……they won't come to places where they will have to mix with the seniors

Moderator: Is it difficult to survive as an anti-ragger?

Several talking together…….in audible…..

Giggling

Person 2: They don't have to do seniors work……. enter their data, thesis work

Everyone giggles

Moderator: Can’t you refuse?

Person 2: we don’t refuse

Giggling……several speaking together…..inaudible

Moderator: what can you do to improve bonds and respect among students and become productive people?

Silence………

Moderator: Since you say there is less ragging among you all, what advice can you give the others?

Coughing

Person 5: In our faculty the lecturers are always present…… but in other faculties the lecturers have given up……. they stay aside and let ragging occur……the lecturers won’t get involved then the seniors behave the way they want and rag the juniors…..

Moderator: do the lecturers just stand aside?

Person 5: Yes they just do their duty and leave…….at other times lecturers don’t get involved when the seniors are ragging

Person 4: Just do the lecture and leave……. don’t get involved in anything else

Moderator: Any other ideas? What else can be done?

Person 1: Sometimes the campuses are closed and the academic period gets prolonged……

Moderator: What else can you do? Maybe something involving the parents……or……what else can you do as future leaders? If you have a child and they go to Arts faculty….. what can you do to reduce this?

Person 3: Punish…….. If you let everyone know that this person did something and was punishes…….

Moderator: Any other ideas?

Silence………….

Moderator: Is there anything else you would like to tell me about this, something that I didn’t ask you?

Silence………….

Moderator: Do you think it will help if you have any activities or clubs, like dancing, volley ball? What do you think? Will it help?

Person 1: It's good, but we don't have time with the lectures…….. there are students with abilities and who would like to do these but there's no time with the lectures and academics…. There is no time to do anything extra

Moderator: I have heard that sometimes the university doesn't give permission…. Is that true?
Several people…. muttering …….. not sure

Person 3: If you have time you can do anything……..

Person 4: you can do anything…….. with disturbing your academics……

Moderator: Is there anything the Sinhalese get together and do?

Several people together…….. Vesak (Buddhist religious festival)

Moderator: anymore ideas to increase the bonds among students? Getting the parents involved?

Since…….

Then everyone speaking softly together……inaudible……disagreeing….

Person 4: the people is that when they get ragged in their first year they decide that they will do the same thing for their juniors…. That idea should be eliminated……

Person 2: shouldn't do they same thing to the others also……..
Moderator: if someone experienced very bad ragging why would you do it to another?

Silence…

Person 3: its less now….

Person 1: Less in our faculty……. not in others…..

Person 2: One entire batch shouldn't do what their told……then it will stop

Giggling

FGD 4

Moderator: Introduction

Moderator: What does "ragging" mean to you?

Person 1: Giving another not only mental person mental harassment but can also be physical harassment……. But sometimes ragging can be a good thing…….but according to what they are going can also be a bad thing…..society thinks……society tries to look down on universities…….so then the one thing they use is the concept of Ragging…….

Cell phone ringing!

Person 1: Because of that ragging can either be good or bad…..

Moderator: What do the others think?

Person 2: Usually juniors……even we thought, before we came that……

Person 3: We were worried about ragging…..

Person 2: Juniors asked us before they came "aiya (elder brother) is there ragging there?"……"What do they do"…… but we also learnt after coming here that the rag is……like an acceptance to campus…… there is a big difference between us and our direct seniors……..we can understand this…. they have better personality….. But we didn't have anything like a rag…… our batch didn't get ragged…..I feel it's how its done…. Some people…..I have heard but not in our faculty, that they harass students a lot…… really…..I don't know what they expect from that…. But a rag is necessary…… I feel it's necessary…..but there should be a limit…. That's my opinion…..

Person 4: It can change according to the person also…..some people can be sick……. People who don't belong to the university put up a false show, to cause problems among students….. people from political parties also influence……

Person 5: Madam I will tell you a little about the Jaffna university……from the Sinhalese students, the ones that lives furthest away comes from about 200km……. when they come here they don't know anything and what's happening here……. We come with different mind sets…. The ragging bought us together…… they told us that we should do these things….. like show these lecturers respect…. You should go like this on the road….. this is how you should behave in the canteen, don't do this, do this……. at times it might have felt like a headache or trouble…. But later we understood why it was said… and that they have said the truth….. things like our discipline was developed….. the small problem that can be in ragging…… like that 'aiya' said….is that some individuals….the person facing the rag and the person carrying out the rag can be different…. If the person carrying out the rag exceeds the limit….then a problem occurs…….

Moderator: So there are good things in Ragging, but what are the bad things? It doesn't have to be here or in your faculty, what are the bad things you have heard about ragging?

Person 1: Those days when you talk about ragging you think about not being allowed to go to the canteen….. give priority to the seniors….. but now when hear about ragging…… its giving harassing freshers…. It has completely changed in universities…. They physically harass…… can be a mental harassment for everyone…… but a lot of physical torture… like stripping….. we have heard things…..sometimes when you talk to other students in the same batch in other universities… we have heard things…… sometimes they don't tell these things… so sometimes nobody know these things…..

Person 2: Sometimes people with a lower mentality try to fulfill their needs…..but…..

Person 3: but from what we know……in our medical faculty…. Now ragging only exists in only 2 faculties…. From what I know…..isn't it (name of another student)?

Another person agrees

Person 3: They have stopped it…… from what I understand they have stopped ragging…

Moderator: Is that in the whole of Sri Lanka?

Person 3: Yes….. in Sri Lanka ragging is currently done in only 2 medical faculties…..

Another person agreeing….and talking softly……

Moderator: What do you think, since you'r from the first batch and when you were coming here what had you heard? What did people say? The bad side?

Person 4: The bad side miss……. There are things I have seen in ragging….. bad things sometimes

Moderator: Where did you see these things?

Person 4: On tv, in the papers…… (not clear)……but then I came here……then there was a rag…… but now I understand… that if that rag wasn't there, who knows what we would have become……a person sees the rag….. there are some in our batch…. There are a lot of students…..they see… all of them see the rag differently depending on their mental capacity….. sometimes all of us have had the same rag, but the other person sees it as a very serious thing….. then that person shows the world….that it was an even worse thing….

Moderator: So your saying that all though all of the students get the same rag, one or 2 portray it as a very serious thing. They can't cope and then give the rag a bad name?

Person 4: Yes……. For me, there are many things I have received…. I have received a big batch…. The reason being…the rag

Moderator: Alright other there any other opinions?

Person 5: In the third year……we have only one year left in campus…. If you consider the Sinhalese….. they are a small number….. but the Sinhalese don't always come together…… unless there was a rag……in this university if there was no rag….. the Sinhalese might not have come together….. I clearly see that when we have a function or a welcome….when we give the first year students the welcome party if we have some work to do….. there is some unity….. because in the first year we came together….. it was built because… actually there's some fun in it……individuals when they arrive come from different levels…. But after coming here…. everyone is made to be like one…. You have to become like one…its then that we have the strength to do something……here this was created because of the rag…..I won't say it's 100% right…..but when we consider Jaffna university….. a majority consider it to be a good thing…….. it creates a unity among the Sinhalese at least….. but there are occasions that this goes the wrong way…… we have seen in other universities some students have even filed cases because the rag was too much…… there should be certain people to prevent the rag turning bad (harmful) when it happens

Moderator: I have seen in certain places that the freshers have been given a set of written rules, what do you think about this? what do you think about the dress code? Do students like it or not? I'm not specifically asking about Jaffna, I'm asking in general.

Person 1: Actually, I don't think the freshers have even read it……. A majority have probably not read it…. Most people don't like to read printed material…… the dress code was given to us the day we came…..we were told how we have to dress all the 5 years and how we should behave…… we got a handbook but we haven't even read that handbook…. I don't think about 90% have read it…

Person 2: We usually learn something from what we see…… if a senior says you must do this…..we don't know why they say that but after a few years we understand that they said this due to this reason….we had something like this…. I don't know if what I'm saying is right…… when we first came we couldn't talk a single word of English….. we can't talk a single word in English with our seniors…. not even the word 'Facebook'……. If we accidently say a word in English, we have to write it till we fill papers…… from that…..this caused…. When we are at a lecture….if we are not attentive at a lecture we miss a lot of thing…. so that was created in us…. When we talk to are seniors we are very attentive….. we know that if we say a word in English how we will have to suffer so we are very alert…. That way when we go to lectures…… our attentiveness remained…. So they created an attentiveness in us in the beginning…… later they came and told us that we did that for this reason….so we received something from that

Moderator: Alright so what you're saying is that ragging has a lot of good things that you will benefit from in the future. If we talk about the violent forms of ragging, where do these occur?

Silence……

Moderator: I mean does this occur in the hostel, or playgrounds…..

Several people …softly….. hostel

Moderator: or at lecture or outside the campus….

Several agreeing….. in the hostels

Person 1: inaudible (another person speaking at the same time)……. That means…in the campus…... doesn't occur in an academic area…..

Person 2: Where groups are dominant…… developing capabilities can be a rag…… that's good… like everyone says…. giving seniors priority, paying attention at lectures…..for all of that it's a group activity that improves….that's alright….. but in hostels they cause physical torture….. in Sri Lanka 'Kelaniya', 'Sabaragamuwa',

Person 3: 'Wayamba'

Person 2: They cause physical harm……. But I don't think……

Moderator: Yes but the very harmful type, like sexual violence and physical violence, where do these things occur? Where have you heard that these things occur?

Person 4: mainly in hostels

Person 5: (very faintly audible) Happens outside the campus also….. not only in hostels

Moderator: where else, do things happen in boarding houses? I have heard that these things occurred in boarding houses in 'Peradeniya'.

Person 1: yes these occur outside also…

Person 2: Yes there are time where these occur outside hostels also.. these have occur in houses outside……

Person 3: What has happened in Peradeniya is the 2nd years anyway live outside the campus…. That means they don't get hostels so they have to live outside….. so when freshers come they sometimes take them to their houses and rag them

Person 4: This happens in the engineering faculty in Jaffna also

Person 5: that means they take them away from the hostels and do what they are doing…. There are instances like that…..

Person 2: what we mean by houses is boarding houses….

Several people agree…..

Moderator: where else does this occur? I have heard they take the students to the playground, for example I've heard the technical faculty keep them in the sun

Person 1: Yes that's normal…..

Yes that happens to all of us……during the orientation week….. there is a madam that also sits in the examination hall and monitors the situation….. we put them in the ground and get them to do some small sports….

Person 3: Madam also comes to the ground……

Person 1: ……that's a normal thing….. that's not where ragging happens…..

Person 3:…to be honest….. we never had anything physical here….. we only got things that benefited us….. that's it…

Moderator: Have you heard anything happening in universities, instances where physical or others forms took place? Maybe about a few batches ago …… Have you not heard anything like that?

Person 1: Yes…… have heard that students from previous batches were hit…. But by now these things have disappeared and gone…… little by little people understand…… if the batch

above us did something, we scold them….then we think a little bit about not doing that to the next batch….. we think like that….. due to this it has gradually decreased…..

Moderator: What do the others think? As the most senior, what do you think?

Person 1: It's like this…… if we talk about hitting….. different people have different levels of patience….. think about it this way…… if the person who is talking with us is aggravating….. this is not only in the campus…. Even in our personal lives….. if an argument goes too far, then it can turn into a fight…… there are occasions like this in the campus…. It could be first years, second years….. things like that can occur….. it's a general thing, not only in the campus….. some people see that also as a rag….. its not something that only occurs in campuses but happens outside also…… but most say if it happens inside the campus that it's ragging…. Or happened due to a ragging incident….. but a majority of these occurrences…. In the campus, in the academic environment….. or the basic rights of a student…. the rights a student has when they come to study at a campus… is not suppressed by a senior or any other person…… for the first years…. most of the time the seniors show them the path… if a lecturer….. let's look at it this way….. most of the time….. if one of us… if a second year student talks with a first year student….. some have a period called the "rag season"….. a time when ragging occurs…. During this period if a second year is seen talking with a first year and a lecturer sees this…. then he thinks, that person is ragging the first year student….. but sometimes we are talking about subjects, or a thing that they don't know…….. if you take a majority…. A lot of lecturers in Jaffna university are Tamil lecturers….. if a Sinhalese first year student asks a question…. Sometimes they can have language problems….but a lot of first years look for a senior that they know or to ask a senior….. when they ask a question from a senior…. And another lecturer sees that, they see it differently and take it as ragging…….

Moderator: Who generally rags? Is it always the seniors, people outside the university, or can it be demonstrators?

Everyone together…..No…..Seniors

Moderator: I have heard that boys only rag boys, is that true?

Several speaking together…..definitely yes….. males are ragged by males….

Moderator: So, males rag males and females rag females? Have you never been ragged by senior females?

Person 1: during the orientation period…….(not clear)….. inside the faulty yes…..

Several together…… just small things…..

Moderator: Like you said before there are certain groups that get ragged more, but does ragging differ according to ethnicity, race, religion, socioeconomic status or any other thing?

Several together…No

Person 1: No, it depends on how the student behaves actually……

Person 2: We call them “kulathi” (high casted?)……. They are actually corned and in a side…. We take them and bring them all to the same level…. And we fix their mentality…. When they leave here, they will have to interact with people in the society…. What is done is their mentality is fixed… we rag these “Kulathi” students and bring them down to one level……

Person 3: A lot who die are also from different mentalities…. We show how try to bring them all down to the same level…. Some people have weaknesses…… this is a good opportunity for them to get rid of those….. some students have big work load or….. they can’t continue….. but by this some students get used to it little by little… some are a little shy or…. Don’t like to come out… shy to talk… but when you go into the society you cant stay like that… but in society for some work…. Life is not only in the university, they have a life after that…. When they are going into that life, they can’t hide in the same manner…. it’s a big failure for them… it’s a big drawback….. but with a thing like this (ragging) their personality improves… it definitely happens….. this is my experience, before I wasn’t a person who would speak out like this, but little by little it improved in the university……

Person 4: Madam, because you spoke about the dress code before…. I’ll tell you a little bit….. in our faculty, during a certain period there is ragging…. There is a particular thing that we must wear during that period….. that means you have to wear short sleeved shirts…. meh…. Sometimes, it could be that I have only one…… I could be very poor and brought only one shirt with me….. but another friend could have 5-6 shirt….. then I have the possibility to ask that person for a shirt during this period…… even the shirt I am wearing today is not my own (smiling)….. become friendly

Person 5: …… there can be different groups….. students with money and without money are never ragged separately in any campus…..

Person 4: everybody is ragged at the same level……

Person 5: you try to make all of them one….. to try to bring them to the point that they act as one…. What we wore today will be worn by another tomorrow….. everyone thinks as one…..

Person 6: ….. In the Peradeniya campus they have….. there are students with a lot of money and poor students…. Then when the student who has money goes to the canteen he eats the best food… because he has money and it’s not a problem….. but when a poor student goes he eats he food that is there for Rs. 20…… then there is a rule in the Peradeniya campus that during the rag season, the student with money and without money both have to eat the food for Rs. 20…. No matter how hungry they are have to eat that…..sometimes the one with money may not be able to eat that food but they must eat that…..in that situation….. when the student with money eats something like that the poor student sees that….. their mentality… they may feel sad…….that’s what they say….. “you may have money and you may not have money, but you are not 2 people but the same. You have to become like one when you leave, you all are the same, you came to the same faculty, you study the same subject because of that you can’t be different, all of you must be the same” that’s what they say…..

Person 3: …..for example things like food…. People watching from outside will say “During this period don’t they even allow you to eat in the campus?”…..

Moderator: Some say that they don't allow the new comers to go to the canteen or bathroom, why do they tell them not to go to the bathroom?

Several people talking together….(inaudible)…… we haven't heard… they don't say that…. No that doesn't happen…..

Person 1: that's how society sees things….. lots of people who say negative things about ragging are not people inside the campus…isn't it…. Its people who couldn't come….

Person 2:…… people who couldn't enter….. smiling (smirking sarcastically)

Person 3:…… to tried to come to campus….

Person 1: …..Society that sees one incident in the newspapers, sees the whole university system as a bad thing…. they want to criticize this…. the people in our country listen more to what politicians say…. They won't listen to what a university student says….. even if we say that ragging is not there or less in this university, they don't believe us…. But if a politician says that in the Sabaragamuwa University something happened 5 years ago…. that a student died…. A girl died… after saying that…. our people…. even though it occurred 5 years ago look at it like it happened today and look at the whole system in that way…. Sometimes that child might have died not because of the ragging….. might have been due to a personal problem…. They don't check and see… they try to twist it as if she died due to this….. that's what they try to say…

Moderator: Do you have any ideas since you didn't say anything from the time you came?

Person 1: Because of the rag…… when we first came we don't know anybody….. we don't even know who the seniors are… as soon as we come….. because of the Rag, you get to know who the people are…. Who is from where…. What are the difficulties they have….

Person 2:… we wrote this in book and memorized it……

Person 3:…. We learn about the other people for an emergency even….. both of us are in the same batch…. We were living in 2 different hostels….. they had about 15 and we had about 15…. When we 15 are together for a year, we don't need to even look at the other people….. we have enough people to talk to…. There's no problem…. We stay separately and they stay separately….. when everyone is brought together and your forced to talk and learn about each other….. and find out details, what illnesses do they have… they question us separately about each one…. If they see us in campus they ask us "who is he?"…. now I know about him completely…. I know what talents he has, what he can do….

Person 4: if someone has an illness the whole batch knows…… what should we do if he's sick…. In which situations does he get sick… in situations like this…. that student may feel shy to say something, but in those situations we will step forward and say don't do this because he has a condition like this… then we get the opportunity to do that….

Person 5: A senior will come randomly and ask who is he?... then we say he's (name).. what does his mother do, what does his father do, their economical status…. We should be able to say everything about him…. We will learn this even out of fear of the seniors…. That's what we call the ragging season…..

Person 2: there was a meaning to everything they did to us….

Person 6: The same way we get to know the seniors also….. we can't directly ask them their names… we have to ask someone else and find it out…. Then we know about them also….. the importance of the is that… when we from the medical faculty…. Even when we go to the hospital….. its good to know… so they have said find out about them and keep…. They ask us, so we learn these also out of fear….

Person 7:…. These thing madam….. when there are very emotional situations…. They may ask you in a big crowd where your mother is…. But there could be apossibility that I lost my mother when I was very small… but then the friend next to me understands that my mother is not there, because he knows my details, he knows everything. What my mother does, what my father does, who earns the money…. Who pays the money, who do they survive…. They know everything…. The seniors in my batch, its difficult for them to know everything…. But I know who has a serious condition in my batch… then if I find out that he was admitted, that he was admitted due to this….. then where is home town is…. Usually madam, every student… if they are from Ratnapura, which part of ratnapura…… if it's Badulla, which village in Badulla…..

Moderator: Does this happen only in Jaffna university or does this…..

Everyone together….. everywhere

Moderator: So everywhere it's like this?

Person 1: Yes its something like this….. what we mean by ragging is this…..

Another person talking at the same time but inaudible…..

Person 2: they path that leads to it can be good or bad…… this is what happens…. the person near you…..

Moderator: so it's because of what one or 2 people do that there are problems?

Several talking together… inaudible

Person 1: when a person exceeds the limit… and goes over…. There should be another person to bring it back to the limit…. There are some seniors… who get angry fast…. Then they can punish us…. But at those times, another senior has to be there to prevent it...……

Moderator: Who stops this, is it another senior, or could it be….

Several… yes another senior…

Person 1: … yes another senior… will come and stop it….

Person 2: ……if one goes over…. Another says "stop that, that's enough"……

Moderator: if you see are serious incident, who do you complain to? To student counselors, or Marshals, mentors, who do you or anyone complain to? Or do they stop it or pretend they didn't see and walk past?

Person 1: …… depend on the situation…. If they can stop it….. if it's a senior, they try to prevent it from happening in the first palce….

Person 2; …..Now imagine… if someone from a batch below me….. that student is ragging a student from a batch below him….. then we have the ability to stop it….. if we go and talk and tell them… Malli (younger brother)…. Don't do this sort of thing, its wrong…… if we try to go and tell the marshal that, it might have happened and finished…… so for us…. There's a thing called seniority…. Then we use if in these situations….. otherwise its not like we use the seniority to be proud or for anything else… we use it to sort out problems…… seniority also comes with experience…… then when there is a problem like that…. rather than telling a lecturer or a Marshal, if we can stop it there it self, a senior will stop it…

Moderator: What if it's a situation you can't control? Or that it's another person who witnessed it happening, will they tell someone or just ignore it?

Several together….. they will tell someone… (rest inaudible)

Person 1: Sometimes a student can't do anything…. in Jaffna, the connection we have with the lecturers is very less… we even talk less to them…. in other campuses they talk with the lecturers…. Like in school how you spoke with the teachers…. But we're not like that…. specially the medical faculty….. we don't really talk to them…..

Person 2:….Here of course the difficulty to talk to lecturers is the language problem…..

Several people agreeing….. the language… language….

Moderator: If you have a problem can you go to anybody?

Several together…… yes….

Person 1: they have the opportunity to do so…. If I didn't like the way I was ragged… I have all the rights to complain….. it's there in this campus….

Moderator: But do they complain?

Several together…. Yes, they tell…… complain……

Person 1: they have said and the rag has stopped…..

Person 2:….. the best example is….. the senior batch…… they had a rag season…. Then there were some who said stop…… they had complained to the UGC or…… (unclear)…..

Person 3:…..then it was fully stopped……

Moderator: The Sinhalese people, you must not be from here, where do you come from?

Person 1: Anuradhapura……

Person 2: Chilaw…

Person 3: Badulla….

Person 4: Anuradhpura..

Person 5: …..(unclear)

Person 6: Matale…

Moderator: These are very far places, so what the difficulties you have? It must be difficult to go home often. You mentioned the language barrier….

Person 1: This you university has a lot of big problems in comparison with other universities…. Let me tell you one problem….. a lot of students are from very far…. Maybe the students from Anuradhapura are the closest ones… everyone else comes from even further…. We get hostels…. Not for all the years…. Only the 1st year and 4th, final year that we get hostels…. but everyone here need a hostel…. So they absolutely have to go to an outside boarding house…. So the cost is more than when you stay in a hostel…. To be honest, some students here might have poor parents… for them this is a big problem… another problem is the students that stay in the hostel… the first years…. To buy food they have to go to other places…. Because the hostels have no canteen or other facilities…

Person 2: Even the canteen inside the university is more expensive than the ones outside

Person 1: ….if we see approximately, with the canteens in other universities…. the cost here is much more…..

Person 2: …… The cost is more than twice as much….in peradeniya a meal that costs Rs. 40 ……

Several talking at once… not clear……

Person 3:….. the food problem is there a lot…..

Person4:….. costs about Rs. 100-150……

Person 2:…. We spend about Rs. 300 per day…… for buses, and things we definitely spend Rs. 300 a day……

Several people agree…..

Person 5: …..Madam… several of our juniors, about 60 of them have received hostels in Induvil….. its about 5.5km from here…. If there is one lecture at 8.00am and the next one is at 4.00pm….. they have to spend twice for the bus….. then they spend Rs.80 only for the bus….

Person 6:…. In the same way madam…. There are no buses to this place…… so the campus students don't have a bus to come to the campus

Moderator: What did your parents say when they heard that your coming to Jaffna university, what do people say?

Person 1: They said in the beginning that there might be problems and were scared…. But after we came here….

Moderator: What sort of problems?

Person 1: Problems as in an ethnic clash…. And we know the war was there…. And that they might treat us differently…. They will treat us badly…. They will harass us….. problems like that…..worried our parents…. but after coming here after about a month, we realized that there was nothing like that…. then our parents also knew there was no such problem…

Moderator: what about you all? As you newly arrived, what did your parents have to say?

Person 2: First they asked me if I want to go or not…… with the environment I was also concerned about what will happen….. will they treat us badly…. Will there be more Sinhalese… I ha those concerns…. After I came… there are a lot of Sinhalese…. So, I didn't feel a difference….. there is a language problem….. its difficult to exchange ideas with the people around…. So that is difficult…. But now it's not that much of a problem…. We have gotten used to it…..

Moderator: Do Tamil students rag Sinhalese students?

Everyone together… No that never happens…..

Person 1: ….they have never even said a word…..

Moderator: I have heard that when students come from far…. They sometimes get ragged more, is that true?

Person 1: not really ragging….. but people in Jaffna have a different mentality….. they think that they are the most supreme….. then they don't like to get involved with other people….. they won't talk to the other Tamils from outside…. That's not a problem among the Sinhalese….

Several speaking at the same time… among the Tamils…. Not seen among the Sinhalese

Person 2: …. The Jaffna Tamils, ignore the other Tamils…. It's a caste problem….

Several agreeing…..it's a caste problem…

Person 2: …. But if you take the Sinhalese…. They mix with everyone… all the Tamils including the ones that come from outside Jaffna…. With Tamils from Jaffna…..

Moderator: So you have never had any Sinhalese, Tamil problems?

Person 3: No…..never….. even if we have a problem now and give them a call…… they will send someone to help find a solution…..

Person 4: Even our lecturers are the same….. just as the lecturers don't talk to us… they don't talk to the Tamils also….

Moderator: So there is no ethnic conflict

Person 4: …….we have male lecturers… they don't talk much with us….. only during the lecture… unless we talk they wont speak…… its common to both (ethnicities)…..

Person 5: That's the same way they treat the Tamil students also…..

Moderator: So, there are no problems due to being Sinhalese, Tamil or Muslim?

Person 1: There's nothing that we can see…

Moderator: What do you think about the introductory course in your orientation week? Does it have some benefit?

Person 1: In our faculty it's good….

Person 2:…… when we look back at it afterwards….. we remember those when we are stressed…. Or look at a video or talk about something that happened then and alleviate our stress…

Moderator: Do you feel the severe forms of ragging decrease due to the introductory program? For example due to lectures like gender based violence?

Person 1: We didn't have such lectures….

Person 2: We had a lecture on GBV

Moderator: Do you have any other lecture that helps reduce violent forms of ragging?

Person 1: We have a lecture called…. SAS(?)…. Society and harmony…. In that lecture they spoke about these things a little bit…. If we take the science faculty, its physical science, biological science and computer science…… the lecture was done as a common one for all 3 of these….

Moderator: Is there anything else that you would like added to your introductory program? Something that you think will be beneficial for you?

Person 1: It's difficult to think of something at once

Moderator: Do you have any recommendations to respect each other and become better individuals in the future?

Person 1: Now madam….. if we take society…. Different people work in different ways… if you see the problems that we have in our university are not the same problems that will be

there in another university…. We spoke about the problem of canteens… a student in Peradeniya goes on strike for free education….. we go for another reason… my idea is that the whole university should focus on one idea…. We should do this…. why should we do this?.... is there a need for this here?..... there's a problem here, so it should be fixed…. We should do things like that… apart from that we can have small small functions….. from our science faculty we have science week…. We have very less sports functions also… it's not enough, we don't join together often…..

Moderator: Are you saying that you want more sports clubs ans societies and things like that?

Person 1: Yes…… we don't have much here….. we have very less here in comparison to other universities..

Person 2: It's very much less here….

Person 3: We also get very less permission to do things…. Even like music programs….

Moderator: Is it difficult to get permission?

Person 4: yes

Person 5: it's very difficult to get permission….

Person 1: Usually madam….we have a big problem….. from the science faculty we annually have a function for Vesak (main Buddhist festival)…. We have a program…. We bring a Buddhist monk from a temple elsewhere…. For a thing like this to take permission it takes at least 3 days ……. We have to take a letter and go and have to put a lot of effort to get it signed…….

Person 6: In the main faculty there is a little….. in our faculty….. madam you didn't see… we only do the Vesak function as a Sinhalese function…… for that we usually get permission… but when we try to do some function like this….. also in the management faculty, we have heard that they don't get permission…

Moderator: Why don't they give permission? Is there any special reason?

Person 7: I feel it's a problem with ethnicity….

Some laughing

Person 4: To be honest madam….. its not just with students… it's a problem with the administration…

Moderator: Is it because they think that there will be ethnic problems?

Several talking together….. inaudible
Person 5: Madam…. You might know…. The Vauniya campus…. They didn't let the students have a statue of Lord Buddha…..

Moderator: No, I don't know about this. What happened?

Person 5: what happened is… 2 students tried to keep a statue of load Buddha in the hostel and they have not allowed it…… they have asked them to remove it…. something like that happened… we don't know exactly what happened…. Because the media always likes to increase it and put it out to the public….. but there is nothing like that in our faculties…. They have everything in the examination hall also…. From all religions…..

Person 6: most of these sort of things come from outside the campus and political parties….. that's they way in the union also… when I was also in the first year…. What we had in our hostel rooms… someone came and broke them with a broom…. This person was staying for a long time as a student in the university but his entire control was by these political parties….

Moderator: So he was staying by repeating his courses?

Several people talking at the same time…..(unclear)…

Person 6: ….Yes….. he received money…. But he had to leave the campus due to a reason… but after he left, there were no problems like that again…..

Moderator: So, there's a political influence?

Person 3: Madam…. There is… people here say… I can remember in the orientation…. They said it without saying it, that this is a Tamil campus….. we received that message and it went into our heads….

Moderator: Who says things like that?

Person 3: We get the idea when we talk to them…..

Person 4:……. Everyone is Tamil…. The lecturers are Tamil, everyone is Tamil……

Moderator: Are there no Sinhalese lecturers?

Person 6: No…. not here…..

Person 1: visiting lecturers come and go….

Person 2: We don't have any…….

Person 5:…… but its like this….. were in an environment that is not Sinhalese….. so it would have been nice to have a Sinhalese representative to discuss our problems…. Someone from the academic side…..

Person 4: Even if there was a counselor…. It would have been very valuable to talk about our problems…..

Person 3: …..Our MSU …..we have a thing called the medical students association…… all the medical students should be included in this but….. but it is completely run by the Tamil students….

Moderator: Why are there no Sinhalese students in these unions?

Person 1: in unions…..

Several people talking at once and unclear

Person 2: to be honest from our batch 2/3rds are Sinhalese…. More than 2/3rds….. but actually….. I think we also should have a place…..

Person 3: …. Madam….. they are of the opinion that this is a Tamil university…..

Person 4: ….. Lets look at it this way…… If I want to do something…. Say I want to have a guiding camp or something in the ground…. I want to do something and get people together… something like that…. if we want to get permission to do something like that…. the chances are less that we will get permission…. Me as a Sinhalese person… if I say I want to do something, I have less chances…. But if I tell one of my Tamil friends…. And try to get it done through him…. It's much easier to get it done….

Moderator: So, what you'r saying is that there are problems with administrators although there are no problems among the students…

Person 1:….. not really…..

Person 2: there are occasions where there are problems among the students….. yes… we have problems with the Unions.. if we want to do something…. And decide lets do something like this….. then we have problems…. Our Tamil friends, then tell us the union said so, "what should we do?"....

Person 3: A lot of these things occur due to politics……

Person 4: Completely due to politics… from students who belong to political parties….

Person 5: They said they have more than 2/3rds in there batch….. in our batch we are more than ½….

Person 6:… most are Sinhalese….

Person 7: but still a union…..

Moderator: Can you not have a Sinhalese union?

Person 1: ….Nobody can…

Person 2: …to think about it…..

Several people talking together…. (unclear)

Person 3: …..in ours…. In the MSU….. the union president has to be….. not even another Tamil student…. But a student who was selected for medicine from the 'Hindu Collage'….

Several people agreeing…..

Person 4: ….thats the most popular school…..

Person 5: There is a thing called and election….. but it's a fake thing….. They say if he's from Hindu collage lets take him….. but the also put up a false show of it…..

Person 3: Then they have an election…. Laughing sarcastically…..

Person 4: The selected person has to definitely reside in Jaffna….. It's the same in the science faculty also….. if you live here only can you…

Person 2: ……..until now…. Even if there are elections…. Nothing changes….

Moderator: So the president of the Jaffna students union is also a a Tamil student residing in Jaffna who went to Hindu collage?

Person 1: ….Yes… he is….

Person 2: Then he works according to a preset schedule…..

Person 3: ..the previous president will say…… you be the president… put this one as the secretary…. Take this one as the treasurer…..

Person 4: So, the person that comes next…. The batch that comes after that…… the president has already been decided from that batch….. they haven't even entered university yet…. There results are out and they know they will come to this campus….. but by then they have already decided who will be the president of the MSU from that batch….. they have already decided…..

Moderator: Is there something more you would like to add? Or is there anything you want to know from us? Or give us another recommendation you would like to give?

Person 1: Madam…. I would like to tell you what I have understood about ragging so far…. from ragging what we expect to do is to bring make the person a little better… we were fixed…… now when we have a junior batch…..I will never use drugs in front of them….. then they respect me….. because I need to maintain my respect I will mind my own business… then we both get fixed…… the seniority also develops…. But when we try to make them focus on something good… you do this function, go to temple….. do this, do that, do this camp….. when we tell them like that and they focus on one thing…. then they…. There is a group of seniors that have no meaning…… there is a group of seniors from our batch…. That have no proper intentions and then they change these newcomers…. That is when these problems arise… there is a group that has no gravity of things…. everyone is focused on one thing, something good….. then groups start to separate…… then these are the one that next year….. will rag or cause a problem….. I have never….. I know my limit so I will never cause a problem…. But there is a senior who is with me, that doesn't care about what happens….. So he has the right….. he is a senior so he has the right to rag….. so then the problem arises from there…..

Moderator: Who are these people? Are they not interested in studies or do they have some connections or?

Person 2: If you look… a lot of them….. their academic side is lagging…. They are last to go to lectures….. things like that…. so they try to do things that take a bad side of ragging….

Person 3:…. Because of their fault……

Person 2: Because of these peoples faults…. That these things happen and… because of one or 2 people's faults that there are problems…

Person 4: Generally, if we take ragging in a university…. It's generally a good thing…..try to do something good for the new students that come ….. to build their unity…. For things like that…. but if there are one or 2 bad people, they spoil everything…..

Moderator: Have you had such bad experiences?

Person 1: Yes…. I have had

Several answering softly….. almost everyone has had….

Person 2: At times like those….. after a while we realize that this senior is different…. When I was a junior…. I knew this senior was different so I can have problems…. So what I would do is….. what I usually do is…. Without going to a student counselor or marshal, I will tell another senior….. I will say, something like this happened to me….. please solve this for me…. Then the seniors will step in and resolve it….. but if I also don't have patience….. then I will go there and then the problem will get aggravated….

Moderator: So the seniors will stop it?

Person 1: In Sri Lanka usually ragging is carried out by the second years…. Under the supervision of the seniors…

Several agreeing….

Moderator: But I have heard that 4th year's harass 3rd year's and 3rd year's…..

Person 1: That is there only in the first year…. And is never there afterwards….

Moderator: Is it only until they give the welcome party or does it continue?

Several together….. only till the welcome party.

Moderator: Does the time you have to give the welcome party change?

Person 1: its different from faculty to faculty….

Person 2: if there is a strike or something…… then the time increases….

Person 3: With the problems we have with Jaffna campus…. Sometimes its very difficult to get a date for the welcome party…..

Moderator: Why is it difficult to get a date?

Person 1: the administration says…. These juniors have come and you have to give a welcome party within 2 weeks…. Then they give us a date… to be honest that time is not enough for us…. Usually when you say it's a welcome, it should be done in a nice way…. We have to spend a lot and decorate and …….. the welcome they expect us to do is a small thing…..

Person 2: Madam…. The welcome is the only thing that is there in the science faculty that can showcase a students talent… usually when we go out of here that opportunity is lost….. but the last function that is there in the campus sometimes….. there are times that we don't get the permission…..

Moderator: So if you don't have the party in the first 2 weeks and then want a date it's difficult to get permission for it, is that right?

Person 1: If we say we had an exam….. there was…. To do a welcome it costs money…. We never take any money from the first year's….. we won't take money from them…. its because this is organized by the seniors… the senior will contribute in a way they can and collect money and go something….. sometimes when they suddenly say have the party on this day…. we can't collect enough money….. we don't have the ability to do the party in a good way….. then we say we cant do it on this day…. give us another day… then when we ask for the next date, they wont give us permission….

Person 2: Due to reasons like this sometimes we can't have the party for about 5 months…. Then it gets pushed back….

Moderator: Thank you for your thoughts and opinions!

FDG 5

Moderator: What is the role of the student union? How is it organized?

Person 1: Members of students union will change every year such as president, secretary. If president from commerce now next arts then science. Medical faculty don't take president post because of work load. Vice president is permanent post for medical faculty. Now president is from commerce. It will change next year. In one year committee will be changed. Our duty is helping students. Now our recent problem is convocation does arranged properly. But in ragging we cant involve directly. It is their faculty union's responsibility. Actually we are under faculty students union. it seems like we are in union so we can do anything. But its under our faculty students union only. We will just give an information to reduce ragging. That is what we are doing now. We will do functions.

Person 2: sports

Person 1: we do sports separately.

Moderator: What financial help do you get? For example, from the university, or any other organization or individuals?

Person 1: nothing much

Person 2: we didn't started to get finance.

Person3: in reality in medical faculty we have canteen. We will get monthly salary. We do alumni get together. If we are in need we will do fund raising activities also. Alumni fund is not permanent.

Person 1: it is according to how passed out students are helping. So it is not permanent.

Person 3: permanent is canteen.

Person 1: in main faculty there are 2 canteens. They are running. They have photocopy centers.

Person 3: its also.

Moderator: do you get funds from organizations?

Person 3: we can get funds only from public individuals. Not organizations.

Person 1: we can collect funds from shops as sponsors.

Moderator: How is the Jaffna student's union related to the inter university student's union?

Person 1: it has good relationship. when there is a flood in Batticalo we went to help them like that if we have any problem here they will come to help us.

Moderator: Do they have control over the activities of the Jaffna students union?

Person 1: no. we are independent.

Person 3: Jaffna student union is the collective body of independent faculty unions. IUSF is the collective body of 9 national university unions. Other unions have executive committee. But IUSF have convener under him there are 6 members in executive committee. From every university there are members.

Moderator: is there anyone from Jaffna university in IUSF?

Person 3: there should be. But…. IUSF has been functioning since 1980… 1970s something. I don't know the extract date. There was an private medical problem so that this IUSF was formed. During war time University of Jaffna independently functioning. They lost connections. With the issue of SAITM. 2nd private medical college issue connections re-established. Now there is no sitting member. There will be.

Moderator: Do they ask you to send students for picketing in Colombo?

Person 1: yes we go.
(someone is disturbing)

Person 3: meeting is going on brother.

Person 1: we used to go. And they also help us during our problems.

Person 3: purpose of IUSF is usually university union is concerned with students of particular university. Also about welfare and all those things. Where as IUSF has few missions and goals. Protecting free education and health care in srilanka and also students rights and those things. And so what happens is a centralized decision towards policy makes by srilankan law makers how that effects university systems and also some common issues like if the councils of srilanka are pointing the society towards even more….. ahhh… lets say… racially charged that short of situation where ever that the students body feels there is a critical situation where we must do something. It is not an executive order. internal democracy within the union. in the last instant where we re-established connections with IUSF in the case of SAITM issue ahhh….. that's how there was a delegation came here. And they explain the issue because independently university of Jaffna or any medical faculty is not aware usually students are not aware what going on in political arena and what dictating our future. So this is the basic model and vision of IUSF.

Moderator: ok

Person 3: im saying this with pretexts. All that IUSF does is not what you have to do. Sometimes within that there might be personal agenda being pushes.some how we maintain democracy.

Moderator: What is your role when freshers' or new students enter the university?

Person 1: you mean how to come?

Moderator: Do you take part in the introductory program? Do you take part in the welcome parties?

Person 1: no. only faculty is responsible.

Person3: IUSF doesn't play any role here. Students are coming directly to faculty or department.

Moderator: do you even go to introductory programmes?

Person 2: that is faculty students union.

Person 3: no. no. nothing. Faculty unions have.

Moderator: introduction part is over now. We are moving to next part. There is a lot of talk in the media about ragging, what are your thoughts about ragging?

Person 2: in media even for a small ragging they will show it bigger. In AHS now a days student count is increasing. So ragging also increasing. Previously it was 70 now tamil students are 40and Sinhala students re 120. Even lecturers cant control it. Media know what is happening in common union not within faculty unions.

Moderator: What do you think about different forms of ragging?

Person 1: every faculty do in different way. In medical faculty we don't do ragging. Some people do ragging like compulsory wearing shoes, tuck in shirts.

Person 3: one thing I have notice recently some faculties have their own dress code. They have certain haircut that everyone should have. Certain dress code everyone should follow. That should be followed throughout introductory period. I cant stand for that. Im not believe to comment on that. I have seen this all.

Moderator: we have also seen it. I have pictures also.

Person 1: in our faculty introductory period is for 2 weeks. But in arts its for 1year. During that they should wear black bata. Haircut. Half sleeve plane shirt. No belts. Its for 1 year.
Person 2: we also have that hair cut. Within Sinhalese. Even after welcome party they should come like that.

Moderator: What do you think about the severe forms of ragging?

Person 1: we are trying hard to stop them. But inside hotel we cant do anything. Out siders cant go inside hostel. If that junior complain about ragging only we will know. He wont complain because he have to travel with his senior. He cant study without his senior. Even he wont speak out. We don't know more about it. In our faculty we don't have such ragging.

Moderator: If you hear of severe forms of ragging occurring can you take any measures to stop it?

Person 1: yes. Already for a problem we did a meeting with all faculty union presidents. We will talk about it in meetings. We will do everything but faculty president should take decision.

Moderator: Do you take any measures to stop the severe ragging? What sort of measures can you take?

Person 3: we will do in future because now even public can identify what is going on in ragging. If we don't stop here it will go without limits like flood. So we are hoping to do.

Moderator: Do some faculties have more ragging than other faculties? Why do you think that happens?

Person 1: yes. In our faculty we can say no ragging. Its reduced. There are differences between faculties and also tamils and Sinhalese. Sinhalese do more severe ragging.

Person 3: and also their way of ragging also differs. Think about tamil student in Sinhala majority area and Sinhala student in tamil area. When you enter a university you have to create more bonds. You have to rely them to live. When I go to a different area I will directly reach my area direct senior who can talk my language for help. So they are using it as an advantage. Senior's mindset also influencing here. If that seniors mindset is good he will take that junior to a good place through unions. If he is having different mindset ragging will happen there. That junior for his survival he is indwelling it and thinks its normal and started to adjust. When it is conveying to future generations batch by batch it becomes ragging culture.

Person 2: now it seems compulsory.

Person 3: it is like a compulsory thing. There is a false justification. A senior student should kept his seniority in perspectives, personality and leadership. By his age and social connections, he is using as an emotional tool to suppress his junior. It is a tool to down our psyche. Some students cant survive here. Some students may from aggravated families. Their reactions my defuse or take this to eruption point.
Person 2: sometimes he will think he should do more than this.

Person 3: some people wants to stop it. They become anti raggers.

Person 1: they isolate him from all the batch mates. So no one is coming like anti raggers.

Person 3: it creates hive mentality within students.

Moderator: What are your opinions about the Technology faculty and the situation there?

Person 2: no

Moderator: no?

Person 3: I went their to talk about it. Problem is there is a students who came in first enrollment. 2013 students. They are the first batch of technology. They don't have seniors. There is only administration. They also create a union. majority is Sinhalese. So there is a Sinhalese centered union. it creates an internal dynamics. Its in Kilinochi and first dean is Sinhalese. Someone from Moratuwa. And first union is there for 2 years because they don't want to give union to first years. They wants to keep this union in Sinhalese hands so that they let every students think racially. They use ragging to achieve it. A students was complained about it. They took action against a union member. This is a first incident. FSP enter here. And show that suspended student charismatic. I don't know the final outcome and how it goes. The person who can be more racist is charismatic. That is what propagating the issue further inside faculty. To my understanding.

Moderator: What do you think about them having their own student's union?

Person 1: they should have but it should be under common union.

Person 2: now its running individually.

Person 3: technology , agriculture and engineering have their own unions.

Moderator: they have their individual union right?

Person 3: every individual faculty have their individual union.

Moderator: is it approved?

Person 3: this is the thing. To own their own union it is their in universities ordinate

Moderator: are they part of university of Jaffna union?

Person 1: they are but they are not coming for activities.

Person 3: thing is Kilinochi is states as subsidiary of UOJ. Kilinochi, agriculture and engineering are considers as subsidiaries of UOJ. Also some in Vavuniya. Recently they have been gazetted in combination as university of Vanni.

Moderator: oh.. really?

Person 3: until it passed cabinet approval it will come under university of Jaffna. If that law is passed and it is gazetted then it will be university of Vanni and they will have union.

Moderator: in your constitutions do you have only tamils students can be in union.

Person 3: there is no law like that.

Person 1: in engineering university president is Sinhalese one.

Moderator: no I mean Jaffna main union.

Person 1: mainly tamils. But not only tamil.

Person 3: it is an unspoken rule.

Moderator: ok.

Person 1: each faculty president will select one person to add in main union. mostly presidents are tamil. So they will select tamils peoples.

Moderator: it is not written it can be only tamil student?

Person 1: no. also Sinhalese are less.

Moderator: Are the student called before they come to the university?

Person 1: every university is difference. I think every university will have that. We think its good to talk. Every senior is different. But we will tell how to come but there are some people who do ragging in calls.

Person 2: 1-2 hours talking.

Person 1: they should call when they said.

Person 2: should not complain anyone. If they ask to sing you have to sing.

Moderator: why it is happening?

Person 2: it is to show themselves to all junors.

Person 1: commonly juniors don't know about each of them. They will just come and go.

Person 2: which students are from which district.

Person 1: because of this ragging only we know who are district juniors.

Person 2: in my batch in total 70 students 23 are tamils. In next batch 50 tamils. No connections between us. They don't even know our names. We will know someone who call us. Others we don't know. They just come to study.

Person 3: mainly it will happen district wise. When someone enter to university from a district he will have a connection with his school. He will gather information who is going to come next year. He will get numbers from is village. It is to record my practical. To do my back lock works. To get connected with a newly coming girl.

Person 2: it is actually unnecessary.

Person 3: according to me university is open and transparent. But it gives a closed loop. Our talks, plans and decisions wont go out from that loop. It is a closed connection loop. Both ends are in seniors hand. I can take them anywhere because they don't know about university. Functions, what is happening, what kind of place is this? They know nothing. They will use their fear and insecurity. I am the first one enter from Gampaha district. My problem was no one here to rag me and help me. No one to show me where is canteen. They are giving justifications for their closed loop connections that they have to know their seniors. It break our batch into many subsides. Some students wont come under any of these subsides and they will be alone.

Moderator: Do the staff and the administration work together when it comes to ragging?

Person 1: yes. There are some staffs to stop ragging. But students are not much obeying them. They will ask to stop. University students union will also support hose staffs.

Moderator: What do you think can be done to reduce severe forms of ragging in all universities in Sri Lanka?

Person 1: can reduce interaction. But it is necessary one. Seniors should think. If a whole batch didn't do ragging next batch will also stop doing that.

Person 2: in our faculty until 11th batch there is no separation. But in 12 batch even Sinhalese don't talk with Sinhalese. So ragging is needed.

Person 1: we can say interaction instead of ragging.

Person 3: ragging and interactions are being used as synonymous. Those are 2 different word. Ragging is hurting someone for one's or one groups entertainment. It is a kind of torture. Interaction is creating a place to get connected with different area, society students. It becomes a problem when you use is as inter changeable. They get confused with this two words. There may be some overlaps. But those are 2 distinct things. Interaction is needed but not ragging.

Moderator: What do you think can be done to reduce severe forms of ragging? in all over Sri Lanka?

Person 3: we cant totally eradicate it. We cant deny when a case came with malaria after we said malaria is eradicated in srilanka. To eradicate something first thing is philosophy. We cannot achieve eradication of ragging without the needs of ragging, mentality which drives it, philosophy which protects it is abolished. Until we eradicate the mental background there is no possibility. As a union we can give punishment or we can track them. Until we eradicate the mentality of seniors to suppress their juniors we cant eradicate ragging. To do that mentorship is important. When a student enter university we have to give them the idea about what is university. Mentorship by lecturers. When a professor tell me what is university, what are my rights here, how can I reach help for security. This message should reach students before their seniors closed loop communication capture them. There is a predatory set whom are looking forward to victimize students in closed loop. Till their mindset changes.. a whole batch should get together to eradicate it. Anyone should bring that philosophy. There only we have a possibility to eradicate it.

Moderator: Is there anything else you would like to tell us or is there anything you would like to clarify?

Person 1: there are anti-raggers in every batch. We can form a group through administration with anti-raggers to identify raggers and give them punishment or suspension. Don't know how possible it is. Most senior batch anti raggers can do that. They will think it will create problem with their batch mates who likes to do ragging. But it is hard.

Person 3: whatever purpose I came into university for, I can complete that purpose self sustainably rely myself independently. We can instill this feeling in the core of every student who is walking through this grow he is not going to be subverted by enemies. Seniority or closed loop connections. Whatever maybe. If we instill this feeling to students who walk through we will not be subverted.

FGD 6

Field notes: Interview was on 17th of march in dept of Community Medicine. There were 6 Muslim girls including 3 first year students and 3 final years from arts and management faculties were participated.

Question: According to you what is ragging?
Person 1: Hurting an individual mentally by talking or physically by doing anything without their wish.

Person 2: It is a kind of violence done by senior students to their newly coming Junior students.

Person 3: Seniors they wanted to change their juniors to the new environment. They use that chance and make fun of their juniors. Their fun becomes hurting to the juniors.

Person 4: (Not Clear…………) because when I was come to the University our seniors gave a lot of works us. We have to call them through normal Call. If to say what's app call is then it's ok. We are from poor family. We can get punish not our Parents. We are getting money from our parents. They punish to our parents also. But they didn't understand. They said if you can't take the part in ragging you will be entering again you will get any place in university in leadership part also. Then I become Silent.
When I call to them they ask to wait dear I wash and come my face then until we want to wait but not cut that call.
Question : how much time it took for them to come back and talk?
I didn't wait like that. One of my friend did it and at the end of that call it took rs 124. I didn't took them seriously. Because I cant purnish my parents for that. We borrow money from our parents only. So it's a kind of purnishing our parents. Mmmmmm… Iam not at all agreeing to this kind of activities. Then I wrote a letter and gave it to seniors. They accept it. (laughter)

Person 2: No no. you can talk. I'm her direct senior. (Laughter) even though I'm collected reloads cards. I'm also I have called to ask seniors but our period have to call to our boys seniors also. After we stop that because in our culture it's not allowed to talk to boys. everything from our period stops we ask to juniors just to call and talk.

Question: do you talk about ragging with your friends?
Everyone: yes
Question: what all you have talked yet?
Person 2: common this we talked about is when we were juniors its very hard they stop us everywhere and ragg. But now we felt after times passed in Jaffna we need some those ragging to mold us the this new place.
Person 1: in our times there were some ragging hurt us. Our seniors was very bad that time. But now there is no much ragging because we are seniors now. We are in a policy that we should guide our juniors in a good way instead of hurting them and getting their scoldings and curse. Because when we were getting ragging from our seniors we never bless them. We trust the words comes from our mouth when we were hurting will make a bad effect on others. If we did same it will damage us.we wanted to guide them in a correct way because number of muslim enteries to Jaffna is very low. We have experienced everything here. We

wanted to guide them to maintain our culture here also. Because for Jaffna people our culture is a different one.

Person 7 : usually we talk about the insidents which hurt us. There was an incident happened one of our batch mate never greet(saying salam) seniors. One of the senior called her and ask her why you don't greet seniors. After that we teach our batch mate that greeting others will bring to good things only. Telling salam means telling them that you will have peace. It will also bring us peace. So we told her come front to say salam. Then we tell our seniors there is no rule to say salam in islam. Don't create fake rules and spoil our religion. After that incident in a Arts faculty general meeting they took a decision that anyone can say salam not only juniors. So we make some change at that point.

That time in a AGA meeting committee ask us what changes you want ? we wrote in a paper that talking in a way to hurt someone's heart is equal to breaking our spiritual temple. If you are a pure muslim you should not break others heart. We wrote it. Then some seniors understood us and they treat us in a good way. But still 1 or wanted to misuse their seniority.

Person 8: In our time there was a meeting called senior bonding. They call all of us including boys to a common place and all our direct seniors will come there. They will ask someone to go front and sing or tell story. On that time we ask our seniors call only boy or girls. Don't call everyone and do this ragging. Like that we call our female juniors to our bording and ask them to sing or tell stories.

mmmm…if you ask this question when I was in first year I would have tell her answers(pointing her direct junior) I was in a different mind set that time. I also wanted to write an article about ragging. Now iam feeling ragging is good. Because when we were juniors we were new to this place. First time we were coming out from parent's control. in hostel no one will care you until 7.30 pm. Wherever you want you can go. You will have full freedom. We dont have any friends or relatives here. If a Muslim girl go to a Colombo university she will get fear to do fault or go out. Because she will have some relatives there, maybe father's friends or mother's friends. She will have this fear in Colombo, Perdeniya and Kelaniya. Jaffna university is not very much related to Muslims. We don't know anyone. There are lots of students who lost themselves in Jaffna. So we wanted to guide them as a parent. But they wont obey us. If we tell like "if you go, we will purnish you" then they will obey us. From the starting we cant control them easily. If we use some strong words they will think, if we go out it will be a problem. After some time they will get to know everything. After some days they will come to know that everything seniors told are only for our safety. Sometimes they will think first we go out then later we can call them and tell. So we talk little harsh. Harsh means not hurting them. Sometimes we have to do this to control them. Sometimes all the students cant tolerate this. Maybe some students will feel bad and think why they are scolding me. We can't identify them from the starting first we treat all of them in a same way then only we identify each of them

If we take arts faulty they have more crowd. Cant observe each and everyone. In arts faculty lots of students went in a wrong path. But in our faculty crowd is not much. If someone go out anyone will call us and tell "your juniors are here now why don't you observe them?" this is the first time they were coming out from parents. When they need love if someone gives, their heart will melt. When a boy talks thy can go in a wrong way. For that only we are giving ragging. When a girl call boy senior and talks boys get their number. Among all seniors someone will talk nicely and be ready to help her. He may tell "don't worry about anything. If you have any problem tell me. I will take care of you." In future this relationship can end up in a problem. I am not telling falling in love is bad its their personal. In the name of love they should not go in a wrong way. Our parents trust us and sent here. For that we are giving ragging. We don't do anything in extreme. Previously in our faculty ragging between Muslims is they should their seniors and talk even girls should call boys. Now we have

changed it to girls should call girls only not boys. In that call we will ask about them and ask them to sing a song. That's all. Then during mixing up we will go and talk. Nothing else we have done.
Person2. Same Thought. Now I am in second year. I have registered. When I ask my friends do we need to ragg juniors or not? Some people telling no. I told we have to do ragging. Mmmmm… We need Racking because mmm… we game to places firstly week got independents rags. That time we need control them self;. Actually I hate black dresses but I like the in ragging period we must wear black dress 1st when I came to the University I Completely disagree do that point but now I like that Because due to that ragging only some people wear bad dresses even they now wearing black.They want even go that much Badly. Because of that only now iam respecting that black dresss and Still im wearing black.mmmmmm…..ragging is need that but is must not Excluded.

Question 2. When and where can these types of violence occur?
Person 3: In our faculty during mixing up only we can give ragging. Other times if we talk about subject matter our lectures will ask "ahhhhh… whats going on? Are you giving ragging"? during mixing up they will give 2hours for each batch like 2nd, 3rd and 4th years. After that night times in rooms only we can give ragging?
Person 4.: In our faculty ragging during faculty hours is low because arts students will be separated to different departments. So a muslim rag muslim is very very rare.
Person 5: During mixing up all our juniors were there. They came to give us hostel. They gave us hostel sheets. During that seniors didn't gave us ragging. They just talk like why didn't you call us? We was new that time. So we got fear.
We are coming from different parts of country. When I am coming from kandy some my seniors will come and tell why don't you call me. She may be from Navalapitiya. Its very far from Kandy. "You call them why don't you call me??" they will ask.
Person 6: hat time there was another group of seniors will tell "for you only kandy people are your seniors not us". In arts faculty crowd is high. If it is a 10 or 15 seniors we can call them but its more than 20 or 30 here. We cant call everyone. There are some seniors ragged us because we didn't call them. We were ragged by our department seniors. Department seniors can rag everyone. No muslim or tamil variation. There will be a 2 hours gap between lectures. They will rag us during that time.

Person2: Even they ragged us in hostel. There were seniors with us in hostel. When we go to hostel for the first time there is no seniors. They went on leave. That time we met our dean once. He told you guys are new to Jaffna .why don't you go and see the places. So we have planned a trip to Jaffna and we went to many places. No seniors know about it. After a month one of my senior saw photos we took in Jaffna fort. Then she investigate me then she made it as a big issue. She sent all those photos to our boy seniors. It gave us big stress. After that our seniors always spot us and ragged us in many ways. We were in pain. We told when we come here we don't have any seniors .we don't know about the rules. When dean told us we planned and went. They throw very bad words. We were very much broken during our first years. According to arts faculty we were the last batch got ragging.
Now we are the seniors we can give ragging. We don't even ask them to call. We don't know much of our juniors.

Person 4 :When a girl in a problem we will think "ohhh she was called me. I know her" then I will tell her to call me and meet me.now I will help her. Due to this calls we can know our junior. Juniors can get to know even their super seniors.

Person 1: In ScienceFaculty there is a chance. In our faculty there are 130 juniors now. We cant remember everyone. It's a big crowd now. When the number of juniors increasing we are moving far from our juniors. We are close to our department juniors. We know our department juniors now. If any junior comes from our area we know them. otherwise when crowd increases our relationship with juniors will decrease.

Question : who do you think will act? Other students, university staff, health staff, wardens, or any other?
Person 1 : University Student Counselor is the main.
Person 2 : Marsel, Dean
Person 3 : Our lecturers used to talk about it. They told us "now you are suffering don't give it to your juniors. I told this to your seniors also. But still you are suffering."
Person 4 : Our Lecturers talks a lot about ragging. Sometimes if we don't took a subject we will have free hours. Others will be in lectures. That time those lecturers will give us permission to sit back in the lectures. It may be the subject we don't need. But still ragging shoud be stopped.
Question: what about hostel warden? Will they help to stop ragging?
Person 3 :if ragging happens they will help us to stop.
We don't need ragging. Because We Are under Graduated in Future some people become professors leaders when become the Juniors almost all the people lost respect. Respectless people . whats the need of ragging. I felt sad. Mmmm… for me that's ok. For boys they rounduping boys. it's very bad.

Question: do you see boys hitting the boys?
Person 3: yeah….

Question : infront of all?
Person 3: yes during mixing up.
Question: If anyone notices any type of ragging violence among the students, what do you think people will do? Or what is generally done?
Person1: there was an incident happened. When I was leaving university. I saw a boy was scolding a girl. Both are muslim. I went and ask. She told he is asking to sing song. He told she is not behaving good. Her friends are complaining me like that. I told that boy its 5pm passed. You don't need to rag anyone. You can go. Then I sent her to hostel. I have stopped some incidents.

Person4: if I saw I will stop. Now only I came to 3rd year. Now we have some power. During 2nd year we are also like 1st years. We don't have any power. We cant rag others. Now we are in 3rd year. Our seniors are busy in doing research work.
Ragging is a useless thing. We don't need it. Its not good to make someone cry.

Person 2: recently when we got our juniors there was an incident happened. One of my classmate told to junior to collect all junior girls phone numbers. He wrote all girls numbers in a paper. I pluck that paper and throw it. Later I had big issues with my classmate boy. But now I can handle it. But junior girls are pitty.

Person 3: we never allow others to play with someone's self-respect. Mostly here in the name of ragging they are damaging our self-respect.
Person 2: our best memory during university is ragging period only.

Question: what they are doing to damage your self-respect?

Person3: when they spot a girl if she talks to someone they will spread that this girl loves that boy. That boy maybe a non muslim.
Question : do you know about students drinking within university campus?
Person 6: yeah they drunk.
Person 2: during welcome party in stage they drink. During welcome party one boy holding the bottle. Drinking and dancing. (showing poster)(laughing)
Question: where the money comes from?
Person 2: they are collecting money.
Person 3: welcome party is organized by second years.3rd years ask this amount of money for drinks. They allocate some money for drinking.
Question: do you see they are using ganya and all the things?
Person2: we have heard. But we never saw.
Question: girls do they use?
Person 2: no
Person3: during welcome party one of my Sinhala friend told she use ganya. Yes she told.
Person 5:During DJ party and 31st night party they drink. But I don't know about girls.
Question : Do they drink after 5 pm?
Person 5 : Yes. After 5 pm they will come drunk. During first year only we have 4pm-5pm lectures. They focus first year students only. They force first year students to drink. That's why they don't let our first year Muslim boys to stay in hostel. Senior muslim students will keep the junior muslim student outside in a known place. They will start to drink in first years then they will be in addiction for life long. After lectures juniors will go in a line. That time there was a senior who drunk and fall down in front of them.
Question : do they drunk and fight?
Person 4:They drunk and fight with boys . they pull girls hand like that happened. There were fights between 3rd and 4th year students during meetings. They want to prove who is having power. Then administration will suspends them. Then again we have to do strikes to bring them back. Recently one of a tamil boy got admitted in hospital.
Question: do you tell everything to your parents?
Person 2: we don't tell these things to our parents. They will feel bad. We adopt it and we are now safe. If we told every small small things they will tell "aahhhhh do you have this kind of tortures?" we used to tell I don't have ragging.
Person 3. We only like this course and came. They don't. already they are missing us. I don't want to hurt them more. So I don't tell.
Person 4: I don't want to hurt them. Evening I call them and ask what you cook today like that. Already they feel bad for our foods here. Normally we used to have nonveg daily. But here now daily we have vegetarian foods only. If they know about ragging they will feel more. Within friends we talk about it. That's all.
Question: do you get ragging from your district union seniors more?
Person 1: we don't have ragging from district union. We are maybe allmost 10 people from same district. So we cant form a union. For Sinhala and tamil people they get ragging from their district seniors. Like north east people.

Person 2 : In Jaffna district they have north and south unions. For eastern they have Baticaloa and Ampara unions. Recently there is a Kurunagela Muslim union present.
There is a district union from Trincomale for Tamil and Muslim people. But they dot rag us. They used to help.

Person 3. Culture is different in every district. Eastern muslims follow different rule in dressing. Sinhala students and people grow in Sinhala are have different culture. For the small changes in dressing muslim seniors will react more. During this situations our district seniors will help us more. They will explain them about our culture and dressing.
Almost our district seniors helps us a lot.

FGD 7

Moderator: explanation about the study and purpose of this interview.
Moderator: In different type of media we can read about serious type of ragging. You can read
such articles frequently. In your group do you sometimes discuss these question?
Person 1: yes.
Moderator: what comments do you have?
Person 1: yes we do
Moderator: Do you think the discussion in media are true/ exaggerated?
Person 2: yes yes. Its there
Person 1:they know how to show things. So they will do. Sometimes they will support staffs. Justice may be in students side but they will write to support staffs.
Moderator: Are there are good ragging also?
Person 4: yes.
Person 1: according to me ragging is unwanted. Then how can it be good.
Person 3: instead of ragging they can give guidance. We can for them to do activities which can
improve their skills. They will do. They can improve their personality.
Moderator: What is serious ragging?
Person 1: physical tortures. Slapping without their permission. Gossiping a girl with a boy. Sexual harassment. These are serious issues. Others are not serious. Juniors don't know about this university. So we guide them to a limited time.
Person 2: for a long time there is a myth juniors should obey seniors. Whatever we tell they will
do. It's in university. If we tell good thing to improve their personality its good for them. They
wont face the problems which we face.
Moderator: what types of violence do your friends talk about?
Person 2: talking in a harsh way.
Person 4: physically touching and slapping.
Person 1: using bad words.
Person 5: calling them after 12 am out.
Person 2: making them to do things which can take their freedom away.
Person 1: using them for our personal needs.
Moderator: how they use them for their personal needs? What they are doing?
Person 1: giving our writing works to them.
Person 2: education related works. Then when they ask us to come to a particular place on 4 o clock if we go in 04.01 they will punish us.
Moderator: ok. When and where can these types of violence occur?
Person 1: most of the times ground.
Person 2: next is on the ways when we go to lectures.
Moderator: on the ways?

Person 2 : yes.
Moderator: ok what else?
Person 3: canteen
Person 5: canteen site. Then hostel also. According to our knowledge there is ragging incidence
in hostel also. Not commonly but we are telling past incidences.
Moderator: According to what we have discussed now who are the possible raggers? I don't ask
names. I have options. Are they senior students, others at the university, people you know/don't
know?
Person 6: direct senior
Person 1: commonly direct senior. Others wont consider us. Within that there are students who
underwent more ragging and they will decide we have to take revenge. They do more.
Moderator: in females? Do females also doing ragging?
Person 1: no
Person 2: maybe Sinhalese.
Person 4: it doesn't happened to us.
Moderator(Ayanthi): can I ask one? Now this Muslims rag only muslims and Sinhala rags only
Sinhala like that?
Person 1: yeah yeah. Like that.
Moderator: you are engineering technology right?
Person 1: yeah.
Moderator: you have only one senior batch?
Person 1: yeah one senior batch
Moderator: they are the only one who rag. not engineering once who come and rag you not
Person 1: no no
Moderator: only your seniors
Person 3: but we had miss our seniors agriculture faculty some thing
(not clear)
Moderator: do anyone from other faculty rag you?
Person 1: no they didn't rag us.
Moderator: Are there any student groups that are more vulnerable to violence?
Person 6: commonly everyone
Person 5: everyone
Moderator: do they spot anyone specially?
Person 1: when they don't obey.
Person 5: they will note from beginning.
Person 1: when someone don't obey their words.
Person 4: when someone behaves differently from others like modern ….they will note them and
equalize them with poor. They will select a poor and modern students and press them to equalize.
Moderator: ok. Is there any differences between Sinhala tamil and muslim ragging?
Person 1: there are big differences.
Person 2: we don't have any sexual harassment.
Person 1: but still Sinhala girls have sexual harassment. That one matter.
Moderator: boy to boy only?

Person 1: girls to girls also.
Moderator: ok. Is there any differences in ragging between urban and rural students?
Person 1: no no they don't divide it.
Moderator: ahh…. ok. If anyone notices any type of ragging violence among the students, what
do you think people will do? Or what is generally done?
Person 3: we did inform our department head. They gave contact numbers.
Person 2: not apparently.
Person 1: we were also scared so we didn't complain openly. Secretly we complain it.
Moderator: ok
Person 1: they took actions for that.
Moderator: ok. Not you. if someone else notice ragging will they help?
Person 3: some people have helping mind so they will help. Others…
Person 2: if we do this they we cant fit with batch. So they wont do. If you are apart from that you can stop it.
Moderator: ok so When people talk about the university, what do they say? Do they say the university is a safe or unsafe environment for you?
Person 2: my family told me whatever happens adjust for 3 -4 months.
Moderator: how people from your home know about this university news?
Person 2: they know because we are telling.
Person 1: they know viral news in viber and whatsapp. Last time they do bucketing in muslim girls and spray water. From that 2-3 girls discontinue their study. They know this news because it
went viral.
Moderator (Ayanthi): do you know a boy in Ruhuna he had some sexual assault. You know recently. You don't know in papers??
Person 1: (not clear)
Moderator: have you heard any story? Not technology maybe agriculture. Science management
have you heard any ?
Moderator: do you know any story like in Ruhuna there is a boy committed suicide?
Person 1: yeah.
Moderator: not this faculty maybe other faculty.maybe in main campus.
Person 2: one person from Kilinchchi from our faculty
Moderator: little louder you have to tell
Person 2: ahh. Before he come to faculty they rag. He cut his neck.
Person 1: one person use tablets because they rag him before coming to faculty.
Moderator: which place?
Person 1: Kilinochchi
Person 2: here only
Moderator: Do you think problem of ragging violence on campus gotten worse, better, or stayed
the same in the last couple of years?
Person 2: reduced
Person 1: I don't know about other universities. But here reduced. I don't know other faculties.
Moderator: ok. Why it becomes low?
Person 1: because they don't ragg us.(laughter) that's why. Also we don't like ragging. I need a
guidance only. Give guidance for 3-4 month. No physical and sexuall.

Moderator: do you have students union?
Person 3: yes
Moderator: do they help to keep ragging in anyway?
Person 1: how they can stop. When they meet us they will tell we don't do anything. Obey us like that. But when they meet their friends they will talk how to do ragging.
Person 2: officially they will tell we will do this and that. But secretly they will do. they will help. They will support
Moderator: do they support?
Person 2: not apparently because in their rules they should not support. So
Person 1: when the union person is against ragging its ok. But if he is not support or against ragging we cant trust him. He will play double game.
Moderator: ok who are the people got selected as union members?
Moderator (ayanthi) : do they have only these people can.
Person 1: no no.
Moderator (Ayanthi): if you want to be in board can you go like that?
Person 1: we take votes like that. We have to elect. Like election
Moderator (Ayanthi): you are the part of student union? Jaffna university students union you have a branch here like that?
Person 1: yeah yeah
Moderator(Ayanthi):if you want to be a president you are from technology can you be in Jaffna
students union. you have to be in science or management?
Person 1: nothing like that.
Moderator: do people addicted to alcohol and drugs in university? Not for you. Others.
Person 1: yeah. There are some changes. There are people who start drinking here. Reason is they have more stress here. So they learn to drink. Sometimes seniors force hostel students.
Person 2: when everyone is drinking in hostel room he can control him in hostel. Sometimes without self-control they started to drink.
Moderator: do they force juniors to drink?
Person 2: yes there are incidents.
Person 1: later he become addicted. Some students they learn to drink in their place and continue
to drink here.
Moderator: ok any drugs usage going on here? Like Ganya?
Person 1: yeah. Ganya is usually used. (laughter.)
Person 2: they said ganya is available outside hostel. If you can do something to stop it.
Person 1: I don't know drug. They use ganya.
Moderator: who is bringing? Students are bringing or someone from outside?
Person 1: students they go outside to bring it.
Moderator: what percentage of students using it?
Person 1: I don't know 20 % using ganya.
Moderator: OMG.. its big
(not clear)
Moderator: do they smoke in rooms?
Person 1: yes. For the people who are not familier with it is a diffecult situation.
Moderator: where are they getting money? Where Is the money coming for alcohol and ganya?
Person 2: from home
Moderator: its expensive know.
Person 1: yeah. Expensive

Moderator: How do they collect money? From juniors?
Person 3: no not like that. Some rich students helps. First he will help them to buy then after they
learnt they will buy themselves. Its true.
Person 2: its called batch fit (laughter)
Moderator: (laughter) do you have to cut your hair wear white shirt like that?
Person 1: yeah. Yeah.
Moderator: what girls wear?
Person 1: muslim girls wear habaya. Others jeans and skirts.
Moderator(Ayanthi): no cheeththa dresses?
Person 2: 2 plaits
Moderator: no dresses and slippers?
Person 2: you have to wear slippers.
Moderator: Have you attended the introductory programme in the university and what does it include?
Person 4: we had common programs.
Person 1: they spoke about ragging. Who are responsible like that?
Person 2: how to do complains? They gave mobile numbers
Person 1: they told everything.
Person 6: they gave marshall's mobile number.
Person 1: our lectures and staffs support us.
Moderator: what is your senior's contribution for this program?
Person 4: they did announcement.
Moderator: any programs? Anything helpfull ?
Person 2: yes they did something.
Moderator: what all they did?
Person 1: they did motivational speech.
Person 2: it was our new day. We don't know anyone. So they make girls and boys to sit beside
and let us to know each other.
Person 1: they share their experiences by showing their pictures.
Person 3: some people have some talents. They helps to know that. They ask random students to
sing. Like that.
Moderator: Do you think it helps in reducing ragging violence among the students?
Person 1: after orientation only we know we can inform.
Moderator: What do you think should be added to the introductory programme in order to reduce
ragging violence at your university?
Person 1: you have to give UGC information and contact numbers in their own 3 languages. Because new students don't know English. You have to introduce in their own language.you have to let the responsible people to talk with students. You can show the videos or pictures how
previous students seek help for ragging.
Person 2: if you show punishments of past events they will get hope. It will be beneficial.
Moderator: what else?
Person 2: nothing
Person 4: you have to do awareness programs. 3 months before they come to university you have

to do leadership programs. Because I spoke to a senior about doing ragging. He told it is to take
your leadership quality out. If you don't obey your senior how you will obey your higher officer
in future. Students should gain confident that no one is under others.
Person 1: we cant get respect without giving it. But here they don't give respect in return. You should not hurt a person who respect you. So do a leadership training for that junior and 2nd years. Responsible persons should conduct that. So we can reduce ragging.
Person 2: ask them to do awareness programs every 3months. We develop some skills in university. Still some students don't know anything.
Moderator: Is there anything you would like to have in your university that is not there yet? Such as sport facilities, meeting places, mentors, counsellors, health clinic?
Person 1: we have asked so many things yet.
Person 3: sources?
Moderator: whatever.
Person 6: gym
Person 2: our faculty is not yet finished. We still need some furniture.
Person 1: first we need lecturers.
Person 2: some of our seniors are always sleeping. Sleeping sleeping and go to ground. When we
ask they told no lectures. So we need lecturers. If we complete it only we can go further. We are
in 3rd semester. Other universities are in 4th semester end. Already we are 8months late. Also we
don't have buildings. We are in short of practical equipment. We are going to nearby engineering
faculty. It is a shame for us and also for our faculty. Please give recommendations.
Person 1: roads.
Person 2: we need good transportations. Transportation is important for a development of a country. Here we don't have good roads. If there is a strong wind all the dust will be over you.
Moderator: What do you recommend should be done for the students to respect each other both
at home and at the university?
Person 1: I don't tell about respect. I will tell everyone should be friends.
Person 2: everyone should have helping mindset. Then they will definitely help. Likewise we should respect others.
Moderator: what else?
Person 4: before expecting. We have to give respect to others.
Moderator: that's all.
Moderator (Ayanthi): where're you all from?
Person 1:ampara
person 2: akkarapattu
Moderator: you go every week?
Person 2: no no. 2week or some times every week end. He is from Puttalam. We will go every week.
Moderator: are you happy with your course?
Person 1: I like only course. I cant like this university.
Moderator: why?
Person 2: Im studying engineering technology. I love my course.

Person 1: climate problems
Person 4 : we have food problems.
Moderator: where you get food? Canteen?
Person 5: our canteen and agriculture canteen.
Moderator: is there anything you like to tell us we didn't ask you?
Person 2: do some awareness programs.

FDG 8

Moderator: According to you what is ragging?
Whispering….
Moderator: little louder.
Person 1: insulting a person
Person 2: Asking our juniors to do the things which they don't like to hurt them.
Person 6: Making a dissociating person to socialize with the university environment. Previously students were living inside a circle created with school, home, and class. I think they need ragging to think about society, they should make opinions to get connected with society.
Person 3: When first years arrived previously studying students making fun with them. Instead of having advantages it has more disadvantages.
Person 4: ragging is the thing between seniors and juniors. So seniors can get to know about juniors information. Some students maybe poor. So we can use ragging know our juniors.
Moderator : do you talk about ragging violence with your friends?
Person 4: can you repeat question?
Moderator: explaining again.
Everyone: yes.
Person 1: There is a difference between universities even between faculties in ragging. Example : Arts & Science faculties.
Person 5: when we think about outside universities, here we have nothing.
Person 6: If he is a senior he have to beat others.
Person 1: People can do lots of good things by using ragging. But they don't do.
Moderator : When and where can these types of violence occur? Can you mention the most common places?
Person 1: Rooms
Person 2: university Lecture hall … sometimes……
Moderator:Hostels???
Person 3: hostels also.
Moderator : which faculties are you from?
Students: management and science.
Moderator : who is management? Who is science?
Students: explaining.
Moderator: which year you are from?
Everyone: 2nd year and 3rd year.
Moderator : where are you from?
Person 2: Im from Anuradapura. He is from Kalmunai.
Moderator: is there any place called "valimadu or valikada"? not boy students tell about it. We got it from femlaes. Can you please tell more about it?
Person 1: yeah.

Person 2: it's a village.
Moderator: not valimadu it is valikada.
Person 2: its prison.
Moderator: Sinhala boys told there is a place called valikada where they take juniors to hit.
Person 1: no.
Person 2: no I don't know
Moderator : don't go and ask your friends.(laughter) we know science faculty seniors took juniors.
Person 1: this is the first time I have heard this name.
Person 2: me too.
Person 3: we didn't heard before.
Moderator: ok (laughter)
Moderator: is there any particular time for ragging?
Person 1 : There is no particular time. It will be more soon after junors came later it will reduce.
Person 5: Whenever seniors have time they will ragg us.(laughter)
Person 6: Before and after lectures. Whenever we go to another lecture hall. When a lectures finish at 11.50 am, we will be free up to 12.00pm. There will be a ragging within that time.
Moderator: do you ever go to your seniors rooms before they wake up to greet them?
Person 1: no. we never did that.
Moderator: According to what we have discussed now who are the possible perpetrators?
Person 1: seniors.
Person 2: seniors.
Moderator : who give more ragging?
Person 1: The person who got more ragging when he was a junior.
Person 3: Person came from a low level society. Person who have studied in a low level school. Person who didn't enjoy any speciality in his life and wanted to enjoy everything here.
Person 5: A person who wanted to make fun like his seniors did to him.
Person 2: they are trying to show their experiences which they took before.
Moderator: is there any unknown people do ragging?
Person 1: no never.
Moderator: Are there any student groups that are more vulnerable to violence?
Person 1: Commonly arguing students. Also they will select active students.
Person 3: They will hit us if we laugh.(laughter) Expressive students, disobedient students. If we don't obey seniors commands they will rag.
Person 2: Jovial and fun persons don't take it seriously. Others will get hurt.
Moderator If anyone notices any type of ragging violence among the students, what do you think people will do? Or what is generally done?
Person 1: When ragging becomes violence we can stop it. We will stop it. If it is our friends we can stop it. We cant stop our senior.
Person 1: if it is a violent only we can stop. When they just talk, ask their names we cant..
Moderator: I am asking violent only.
Person 1: we can stop.
Person 3: yes.
Moderator: what will you do:
Person 3: violent means hitting. Come machan… come come. We can tell lecturer coming. Like

that.
Person 5: we can solve if it is our friends.
Moderator: Who do you think will act? Other students, university staff, health staff, wardens, or any other?
Person 1: everyone can
Person 2: vice chancellor
Person 3: Marshall, Lecturers, Hostel warden.
Moderator: other staffs?
Person 3: they don't interact much with students.
Moderator: ok.
Moderator: When people talk about the university, what do they say? Do they say the university is a safe or unsafe environment for you?
Person 1: When we were in first year my family members were in fear. Now its ok.
Moderator: what that time?
Person 1: little fear
Moderator: do your parents think you are safe? What your parents and relatives think?
Person 2: There is no problem in my family. Because my parents were studied in University. But in our area there are different opinions about Universities. They think there are more violence happening against women in universities. There are sexual tortures in universities. They think 3 times free abortion for university students. Some people ask me if you go to campus you will get an abortion is that true? There are such opinions in society.
(two more students joining)
Moderator: are you muslim students?
Person 7: yes.
Moderator : sit. (explain about interview.)
Moderator: do your parents think you are safe?
Person 7: yes
Person 8: yes
Moderator: do you know anything about seniors drinks in parties and forcing juniors to drink?
Person 3: no. it don't happened for us.
Moderator : We don't ask about Muslim community. We know you take your juniors separately. ?
Person 3: others don't call us for their ragging. So we don't know.
Moderator: can to contact any lecturer or anyone if you have a problem?
Person 1: in Jaffna university we have.
Moderator: do you know about coming drunk for meeting and breaking chairs and fans? Is there anythink happened like that?
Person 2: no. nothing like that.
Moderator: no. nothing. ..?
Person 2:in meeting….. no.
Moderator: in parties, welcome party?
Person 1: welcome party……..(laughter)
Person 2: ragging…??
Moderator: no ragging. Some students told final year students told our friends will come drunk and fall in front of others. They don't come with their consciousness. When they go in suspension we do strikes and take them in. they told like that. We want your opinion about this.
Person 2: there are people who went in suspension for doing ragging. But there is no people like

this.
Moderator: Do you know anything about people posting news about ragging inside universities?
Person 3: now a days lots of news comes in medias so people think ragging is more now.
Person 1: ragging is reduced now.
Person 2: Peoples are doing online complaints. Reporting news directly to UGC via internet.
Person 1: Sometimes media enhance the news and releasing fake news for their profit.
Moderator: do you think ragging is reduced now a days?
Person 1: its reduceds .
Person 2: it is reduced more than before.
Moderator: Do you think district union have influence on ragging? Do your district union rag you more?
Person 2: yes.
Person 1: Our district union will rag more and hit more.
Person 2: After I pass my A/L they call me and ask me to come to a common place. If I go they
will hit me. So I avoid it. I never go to thing king of thing. Later they make issues with that. They told we will separate you from the batch. I was ready to face everything. There is no such
things in Sinhala areas. But in north east area it's a big problem.
Moderator: What does introductory programme include?
Person 1: they told us about how to complain about ragging, they introduce about BBC online updates and counselling.
Moderator: What do you recommend should be done for the students to respect each other both at home and at the university?
Person 1: Reason for ragging is there is no much interaction between seniors and juniors. They
should increase the interaction time.
Person 2: They should change the dress code. Time duration for welcome party should be reduced. District union should not give ragging. This method should be changed.

FGD 9

Moderator: explanation of study.
Moderator: In different type of media we can read about serious type of ragging. You can read
such articles frequently. In your group do you sometimes discuss these question?
Everyone: mmmm… yes
Moderator: what all you have discussed?
Person 1: mmmmmm……..
Moderator: tell me about some recent events.
Person 1: if we know anything about other university ragging news we will talk about it.
Moderator: ok. Did you see any news about your university in media?
Person 1: no nothing.
Moderator: Do you think the discussion in media are true/ exaggerated?
Person 2: they exaggerate.
Person 1: they exaggerate for their needs.
Person 3: there was a news in media. They put our faculty name. When we did inquiry we got to

know it is not from our faculty.
Moderator: what happened?
Person 3: here we have Buddhist temple but there is no hindu temple. So they fight to remove Buddhist temple from here. It came with our faculty name. but it is done by another faculty from
here.
Moderator: ok. Are there are good ragging also?
Person 4: ragging means its bad only. Then what is good there?
Person 5: sometimes we can learn something. Our tamil seniors did not rag us. We can learn something from that. In the same time Sinhala students had something different. That is ragging.
Person 4: we cant tell its ragging. Its just dress code. Wearing Long skirt. That's all. Its enough.
Person 6: asked to walk in line.
Person 5: for Sinhalese its bad.
Moderator: What is serious ragging?
Person 5: serious physical ragging is there. In our batch one girl become tensed and she got admitted in hospital. She had mental disorder and they gave outer bounce.
Moderator: to that girl?
Person 5: no for raggers.
Moderator: ok. What else is happening?
Person 5: they wont share anything with us.
Person 6: we are not involved in ragging. So they don't share anything with us.
Person 5: they wont talk anything when we were there.
Person 2: they will cooperate well for ragging. They wont complain. They have that understanding.
Person 6: from our batch we did complain during a meeting on behalf of our Sinhala students but
they didn't talk anything about it and they ask them to chase us out. (Coughing)
Moderator: what types of violence do your friends talk about?
Person 2: what is that?
Moderator: sometimes there will be violent ragging like hitting. Do you talk about it?
Person 2: we don't have that experience.
Person 3: there is no such ragging we had. We know they are doing for Sinhalese.
Moderator: When and where can these types of violence occur?
Person 2: Gound. Canteen. (Laughter)
Person 3: main canteen.
Person 4: ground.
Person 3: now not much ragging in ground.
Person 5: we don't have proper lecture halls. So during we move from one place to another.
Person 4: outside.
Person 2: not inside university. They will take them outside.
Person 1: they have a temple for them.
Person 3: first they will select a place then they will take their juniors there.
Person 6: the way to their temple. Our university surrounding is like a forest. So they will take their juniors to their temple in the name of pooja. They will do meeting. We don't know anything.
Person 5: if we ask they will tell we went to temple.
Person 2: they will go with their temple dress code.
Person 6: whatever you can think, no student will expose their senior.

Person 1: even the victim itself don't tell us. they don't even tell us. They support that much. One day they will give it back that's why they are taking it now. It wont stop.
Person 3: because they thinks that is university fun. That is university life.
Person 4: if you don't engaged in ragging they will isolate you.
Person 2: within tamils noting like that. They are being like a community. If one person got isolated its very hard to live here. So they are cooperating everything.
Person 5: it is a tamil area. Their language is different. If they got isolated they cant live here. So
they are adjusting everything.
Moderator: According to what we have discussed now who are the possible raggers?
Everyone : seniors.
Person 6: currently our batch.
Person 5: yes.
Moderator: direct seniors.
Person 5: yes.
Moderator: others at the university, people you know/don't know?
Person 5: seniors wont allow that. Now our batch wont allow others to rag our juniors.
Person 1: now we have 3 batches here. Our batch will do ragging. super seniors will give ideas.
Moderator: do you get rag from agriculture faculty?
Person 6: no
Moderator: only engineering or bio?
Person 6: bio
Moderator: iam just asking you. Is Sinha people have more ragging than you?
Everyone: yeah
Person 3: because they like it.
Moderator: you are from Jaffna or ?
Person 3: we are another district. But some peoples from Jaffna Trinco.
Moderator: Sinhalese ones say no we are not the one. But everybody knows.
Person 3: they like it. We don't know why they like it.
Moderator: they like it?
Person 3: yeah all the peoples are like it.
Person 6: they want to do again their juniors. That's why
Moderator: so they like to do that?
Person 3: yes. They think ragging is one of the fun of the university life. So they can enjoy. After
ragging they will become very friendly with their juniors.
Person 6: next year juniors will do ragging.
Moderator: so what sort of things they do? We have heard they do very bad things?
Person 6: sexual harassment
Person 2: physical harassment
Moderator: boys or girls?
Person 6:both are
Moderator: like what? For example?
Person 3: actually we don't know.
Moderator: what you have heard?
Person 3: ahhh. Act like some sexual activity.
Person 6: ask a fat girl to do jumping and they watch her parts.
Moderator: ohhhh….
Person 6 : shaking body.

Moderator: girls are doing this?
Person 6: they support. They give ideas to boys.
Person 3: they are not separated like we are boys and girls. They both are same. Whenever they
do ragging they think they are just seniors.
Moderator: where they are doing?
Person 4: ground.
Person 1: anywhere. Ground temple canteen.
Moderator: did you see any pornography things they do?
Person 3: we don't see. Just heard.
Moderator: they hit only boys?
Person 2: one girl I heard pull her dress.
Person 3: she cry. She don't like ragging so they pull her dress.
Moderator: ohhh. Do they touch her body?
Person 3: some girls are ok with that. Some are against with that.
Moderator: do Sinhala people are friendly with you?
Person 2: friendly ragging time only…
Person 5: ragging time only they separate we separate. (laughter)
Person 3: batch fit.
Person 4: batch fit.
Moderator: Are there any student groups that are more vulnerable to violence?
Person 2: beautiful person
Person 5: fat people
Person 3: who is arguing.
Person 6: if you argue once its ok. You are the one.
Moderator: is there any difference between tamil Sinhala muslim ragging?
Person 6: tamils and muslims don't have any ragging. Muslims have ther dress code. Fardha. Tamils long skirt. Nothing we had other than this.\
Person 4: just they will scold if we don't greet. That's all. Nothing physical or sexual.
Person 6: also for our juniors.
Moderator: is there any difference in rural and urban students during ragging?
Person 3: everyone will change after they enter university.
Person 6: everyone will get same ragging. No differences.
Person 3: person who do more ragging is the powerful senior according to juniors. when result
came all are repeat. (laughter)
Moderator: ahhhh If anyone notices any type of ragging violence among the students, what do you think people will do? Or what is generally done?
Person 2: we don't even let our boys to rag. No night calls.
Person 6: if someone call our juniors outside we told them don't go. Tell us we will see.
Person 1: we already told our district juniors if someone called tell us first.
Person 5: morning 6.30 all Sinhalese have to go to canteen. We told our tamil juniors don't go.
After you eat go to lecture hall.
Person 1: now they are only using technology canteen. After ragging period only they will use all
canteens. They will come in one queue and buy food. 2-3 people can buy same time but still they
will buy in a queue.
Person 2: everyone should assemble in that place. If they miss one student they will wait then

they will go to lecture late.
Person 1: when we were juniors we will go early for lectures. They will come late and stand outside.
Person 6: they will get scolded. But still they wont open their mouth.
Person 1: they will tell food came late.
Moderator: who do you think will act? Other students, university staff, health staff, wardens, or
any other?
Person 2: lecturers
Person 6: they should not be ready to be ragged.
Person 1: there are some people. Some committee.
Person 6: unions , student counsellors. Even if we inform they will come to stop.
Moderator: who else?
Person 6: all university staff.
Person 2: warden
Person 6: warden will be very much protective. If juniors are going outside she will help them to
escape from ragging.
Moderator: people talk about the university, what do they say? Do they say the university is a safe or unsafe environment for you?
Person 3: because it is Jaffna, a tamil area so they have a good opinion. Because in Jaffna ragging is very less for tamil people.
Moderator: do they think it is not safe, it is danger?
Person 6: no
Person 3: no
Person 6: they have fear about this environment. It is like a forest. Previously we went to lectures
here so its ok. Now we are going to technology faculty. So they have fear.
Moderator: do your parents know about this faculty aging issues?
Person 6: yes. We tell them.
Person 2: I don't tell.
Person 5: I used to tell them. We are not doing that
Person 6: if we did mistake we can hide it from parents. We never did this.
Moderator: Do you think problem of ragging violence on campus gotten worse, better, or stayed
the same in the last couple of years?
Person 2:hmmmhmmm…
Person 4: they are not showing out. But its there inside. Like peradeniya.
Person 5: they said zero ragging in peradeniya. But our friends are telling us.
Moderator: do you think here you have raging?
Person 5: yes. In every faculty.
Moderator: is it not reducing?
Person 1: no they are hiding.
Person 5: yes.. they are taking actions but still they are doing again. We were unwantedly there
in home for some days because of this.
Moderator: do you have students unions?
Person 4: yes
Moderator: do they help to reduce ragging?
Person 6: they are also students so they wont bother about it.

Person 1: they will tell any important announcemnets. That's all.
Person 2: they will become very close even after very bad ragging for 3months.
Person 6: they did same to our batch also. Now our batch girls are very fit with their seniors. They will cry and tell us their problems. If we complain they wont expose those seniors. Then we will be in danger.
Person 3: now they are very fit.
Person 2: they are telling now they will tell their children about how much they have enjoyed in
university.
Person 5: during their bucketing event bathing in mud we felt very bad. But they are telling it is a
part of fun.
Person 6: we don't have welcome party or bucketing. Because we don't do ragging. Our seniors
didn't rag us. So we didn't give ragging to our juniors.
Person 5: we didn't cooperate for ragging.
Person 6: for tamils they don't give welcome party and bucketing.
Person 1: they don't give welcome party inside university. They did outside in a hotel. For us in
the name of welcome party they give a tea and a role. That's all (laughter)
Moderator: how students got selected as union members?
Person 6: its according to student union. this year tamil president. last year Sinhala president.
Person 4: secretary Sinhala.
Moderator: are they girls in union?
Person 1: no boys
Moderator: if you want to go to the union, can you go or no?
Person 3: no only boys will go.
Person 6: they don't like to go.
Moderator: is there any rule girls cant go?
Person 6: no no. already they will fix who is going to be member. If someone who did more ragging this time, he is the president next time. Its fixed.
Person 5: its very hard for girls to go everywhere. So they put boys.
Person 6: they are not doing anything like that. They just see faculty problems only. Next year president from our batch. So they will put the guy who torture very much as president and put a
tamil guy as secretary. Just for a name.
Person 2: for all functions they will decide and organize everything. We will just go and participate.
Moderator: you guys are newly coming out from home and staying alone. Do you have any friends who are getting addicted to alcohol?
Person 1: no girls
Person 5: not in tamils.
Person 5: now they are staying in outside boarding so we don't know what is happening outside.
Person 5: its more in boys.
Person 4: it is there within Sinhala girls
Person 5: there are girls also.(laughter). Its there. You all don't know
Moderator: do your seniors force you to drink?
Person 6: maybe for boys.
Person 2: for boys.

Person 4: Sinhalese doing.
Person 4: it was there in welcome party.
Person 2: no it was there as their wish. With side dish and all.
Person 6: during ragging also.
Moderator: do anyone using “Ganya” in hostel?
Person 2: not now in hostel. They are using from outside.
Moderator: outside means?
Person 1: now they are staying with freedom in outside boarding.
Moderator: is that became an issue?
Person 2: no. they are doing with their wish.
Moderator: Have you attended the introductory programme in the university?
Person 1: yes.
Moderator: what does it include?
Person 1: about course outline.
Person 3: about university
Person 6: one full day they talk about how to handle ragging.
Moderator: what your seniors did?
Person 1: nothing.
Person 5: they gave tea (laughter)
Person 4: they ask them to come officially. So they didn’t come. Only little seniors attented. They did announcements.
Person 2: they know each other before they come to university. They will create a group and talk. In our batch they asked a girl to form a whatsapp group and seniors also join there like a junior. They will see everyone is talking. If someone told I will complain about ragging they will
notice that person and do more ragging.
Person 3: like that seniors create 2 whatsapp groups. One will act like helping us another will do
ragging. So if we talk about ragging in another group they will know.
Person 4: if we are angry one senior will come and talk nicely and wont let us complain.
Moderator: Do you think it helps in reducing ragging violence among the students?
Person 2: yes. They gave many numbers.
Person 6: spoke about student counsellor.
Person 4: how to complain in UGC
Person 5: they introduce staffs and student counselors.
Person 6: they did very much to stop but until Sinhala students cooperate you cant do anything.
Now they are being ragged after some time they will tell we are going to rag.
Person 5: cant change it.
Moderator: What do you think should be added to the introductory programme in order to reduce ragging violence at your university?
Person 1: they have tried maximum.
Person 2: they should think..
Person 6: they should how what is their mistake.
Person 2: continuously you should put exams quiz everything. (laughter) or you should put seniors result in front of juniors. Everyone is repeat so…
Person 4: you should show who all got outer bounce. They should know what you will do for ragging.
Person 6: they know if you give ragging all students will do strikes and take them back. Even those juniors will do strikes. So they don’t have any fear.

Person 2: 2 days before they call 5 student to admin because of ragging complain so immediately
they spread a message no one should attend lectures. So they didn't went to lectures.
Person 6: hey will support immediately. They started striking then tamil boys create problems then they went.
Person 5: they will create a lie immediately.
Person 1: by doing strike they will change the lie as truth. If we went to complain they wont trust
us. They have a cooperation.
Person 3: if they strike we will go to lectures.
Person 6: sometimes our boys also support them but we are one once who didn't support them.
Person 2: they also have some people. But they will isolate them. There are 2-3 people like that.
Person 1: no functions. No t-shirt.
Moderator: Is there anything you would like to have in your university that is not there yet?
Person 4: shops.
Person 5:we have nothing here.
Person 2: our faculty is there.
Person 1: no transport
Person 2: no canteen.
Person 5: even for a photocopy you have to go to junction. Miss will give only one copy. We are
110 people. If we take it there. They will tell machine is heat. We cant take now. Also it wont be
a clear copy.
Person 2: food is not good. In Peradeniya government will pay half. But here we have to pay full. They are giving hostel so we can manage. Otherwise it will be very bad.
Person 1: in hostel if we go at 6.31 pm we cant go inside.
Person 2: its ok. It should be there.
Moderator: anything else? Such as sport facilities, meeting places, mentors, counsellors, health
clinic?
Person 1: we have.
Person 2: if we don't have sports facilities they will make it.
Person 5: health clinic, sports all we have.
Moderator: What do you recommend should be done for the students to respect each other both
at home and at the university?
Person 6: it is their humanity came from birth. Until a single person thinks to be happy by hurting others ragging will be there.
Person 2: when we see our seniors who rag us they are from very poor families. They cant study
without Mahapola. But still they are the violent raggers. They are not even studying. All are fully
repeat. They are not thinking.
Person 6: all those raggers will come to all exams with us. Even for English they will come. In
orientation program you should show their result and tell them these peoples are coming here to

rear cows.
Person 2: you should do that.
Person 1: they will be in front for ragging.
Person 2: ahhh….
Person 6: you have to tell if you do this you will also become like them.
Person 2: you should tell them what hey will do if you get outer bounce. They will cut
Mahapola.
Some students from very poor family so they will know.
Person 6: no need to threat them. You have to do that. If they do strikes they will they the
outer
bounce students back. You should not do that. If they really did something and caught with
evidence you should punish them. No matter whatever they do.
Person 2: you should tell their parents.
Person 6: send them with their parents.
Person 5: sometimes they will feel proud for whatever they are doing. They will tell their
parents
from room that seniors are doing ragging. We are cooperation. We respect our seniors. They
feel
proud.
Person 6: if anyone doing strikes, don't you want study? Go home. You have to send them
also.
Person 2: yeah.. we tamil students attend all lectures. But when they will outer bounce they
will
give it to everyone. We are also get affected. Lecturers wont separate us like tamils and
Sinhalese. They will show their angry as a whole.
Person 2: they will try to hide their fault in the name of strikes. They wont let us go to
lectures.
Even it is 5 students you have to do lecture. They should cut their attendance. So they will get
fear if they don't o their GPA will become low.
Person 6: -3 days they wont come to lectures then automatically they will come. If you gave
outer bounce it should be there whatever happened. They should inform it to their parents.
They
should be insulted. If you have evidence then why you have to worry? Why university staffs
are
not doing that?
Person 2: university management is not good. They should send them home. They will talk
about
it 2-3 days then they will forget. So again they will start.
Person 6: in meetings they just tell we know your faces we are watching you. But they treat
both
of us same. No difference. First university should change. Staffs should change. until that
nothing will change.
Person 2: they will say its student's freedom. Some girls are there in our batch they are doing
very bad ragging. They don't even care it when staffs talk about them. They are not getting
fear.
Person 6: staffs used to say we have photos we will see you. They will be like "do whatever
you
can"
Person 2: they think if they got outer bounce hey are best. They don't have shame.
Moderator: what those girls are doing?

Person 2: girls are guiding boys how to do ragging.
Person 5: they don't have differences. Girls only knows how to irritate girls. Show they are guiding boys. They are not like us. They will do everything together.
Person 2: at the end of ragging they will ask girl juniors to write about boys and boy juniors to write about girls. They will write very bad thing. But they will enjoy that.
Person 4: they will put names. It will have very bad meaning. For the whole 4 year they will use
that name only.
Person 6: some days they will feel bad for those names them after some time they will put those
names to their juniors.
Person 1: every single name is very ugly.
Person 6: in university staffs should be strong. They should punish them.
Person 1: one day seniors drew a teasing picture about a lady lecturer. So from that she don't take lectures for the whole batch. After that during a problem in hostel all seniors didn't went to
lectures. They told they will treat all same only. So they wont go. They ask us to not to go to lectures. But we went to lectures. But they gave us outer bounce. It is a big mistake of university.
Even though we went to lectures we went outer bounce for 3 months.
Person 3: will you help us in anyway?
Moderator: no advantage or disadvantage.
Person 6: you advise them to change their system. If someone do a mistake ask them to punish
them
Person 2: no problem if you tell about us. But ask them to change system. In schools if there is a
problems they wont go to roads. But in university for everything they will go to roads. So even if
you do strikes for jobs they don't bother.
Person 1: its good if we get something good through this.
Person 2: during that drawing issue we went to lectures to obey her. But she was angry with us
also.
Moderator: who drew that?
Person 2: they is a gang for this kind of things. You don't know from where materials are coming but they will do in 5 minutes.
Person 4: they can do very good translation. Every one is thinking we are helping them. But they
have very good translators.
Moderator: what else they are doing?
Person 1: asking foods.
Person 4: in boys hostel they ask juniors to skip meals for 2days.
Person 2: some good habits also they are developing after ragging. If someone don't eat they will
share. They develop equity.
Person 3: sometimes we will feel very bad when we see them. When someone passed away they
will collect money and do something for that family.

Person 1: when there is a program all of them will be there. But in tamils only we will be there.
Person 6: they are fit for everything. No matter Its good or bad. We like it.
Person 4: they have a thought they have to show their majority here. Even in a small thing they
will show their majority. That's why they are not coming under control. They will be motivated
go go do do don't stop like that. They are thinking in this faculty Sinhalese are majority. So because of them only this university is running.
Person 1: they are doing all programs. They think if they don't come there wont be any crowd here.
Person 6: we should change Srilakan system. Within tamils 1.1 z-score cant enter university. But
with 0.7 score they are coming here. It's a big mistake.
Person 2: government systems should be changed.
Person 3: one time we complain to president when he was here about canteen. But he didn't do
anything.
Person 6: majority is not a problem. But they are doing all wrong.
Moderator: ok.

FDG 10

Moderator explaining….
Moderator :According to you what is ragging?
Person 1: It is not only hurting a person physically but also mentally. Hitting a person, hijacking
others property or teasing a person in front of others. When a person is crossing indirectly teasing
/ mocking that person by using words is also ragging in my point of view and they hurt others physically.
Moderator : again explaining question
Person 2: All are thinking after ragging they will develop a bonding and an interactive relationship.
But I will tell ragging is not necessary. Ragging is giving problems mentally and physically.
Ragging is for an outstation student to adopt this environment. But I think ragging is not necessary.
Moderator: Anyone else???
Person 3. Senior students trying to show their power to their juniors. They can use their power.
They can talk with their juniors but here they are misusing it. In my view ragging should not hurt
others. All things we are doing to hurt juniors are ragging.
Person 4. Hurting a person mentally is ragging. But I don't tell ragging is no need completely.
Because when juniors come we need a relationship with them. Now I am in 4th year i have a good
relationship with my juniors. When I was a junior I don't know my seniors because they never
come and talk with me. We should not hurt them mentally. We should not force them to talk. It

will be good if we behave like that.
Person 5: Ragging is……….. mmmmm…..university culture is juniors should obey seniors. We
should not talk to our juniors in the name of ragging. Juniors don't know even their area seniors.
Without hurting them we should talk them nicely. Then only we can know their problems.
Question 2: Do you talk about ragging with your friends?
Everyone: yes.
Question 3: What all you have talked yet? Your experiences???
Person 5: mmmmm…. When we were here our seniors were the best guide for us. But now students don't even smile at their seniors. They don't even wish. They won't lose anything if they
smile. They will behave like they don't notice us. Because we never treat them like how our seniors treat us. We never go to ragging. Even we don't go to their orientation programs. We used
to talk about how we obey our seniors during our first year of life and now how our juniors are
behaving. Our seniors make our dress code. We wear according to that only. We used to talk about
our juniors dress code. Now our juniors dressing is very bad. They wear short dresses over their
knee. Our boys used to make fun about their dresses. For boys it's a pleasant thing. But students
things its good. How to say?..... they enjoys their dressing. They think it's a style. Some boys will
talk about it with us. We used to talk about it within our groups.
Person 6: If we talk to the juniors about dress code suddenly they we tell others we are ragging
them. No one will try to find the truth. Everyone will support juniors if it comes as ragging. Like
marshall. So we used to talk within us about it. If we try to talk with juniors it will registered as a
ragging.
Moderator: what all you talk?
Person 6: mmmmm… About dress code and lack of obedience.
Person 7: There is a time for ragging even if juniors are ok with that. 1st year students are talking
with final year students even after 6pm in the stone bench. That time we should tell them to go to hostel. First years also laughing with seniors. I saw a girl sitting on a final year student's bench after 6pm and laughing with seniors.(doing gastures how they laugh) After 6pm there won't be any students in university. Even students won't come in a stable mind. They will be drunk. Everyone knows it. They don't know what will happen to them. When we saw that we get anger. They should think about their protection. They should think I should go back home safely. Then only they can lead a good life here.
Moderator: what else??? Explain question again.
Person 8: We used to share our experiences of ragging. During first years we were suffering. They rag us in hostels. Singing and acting in dramas. They scold us many times. Now we talk about that and we feel happy. We had a best experience of ragging.
Person 3: (talking in very soft voice) usually boys talks about ragging in a different way. If a boy

got ragged in future he will think how he could develop that ragging and give it back in a better
way. They will talk in which way they could improve it. Generally boys are affected by ragging
and they are the people who give it to juniors.
(whispering..)
Person 2: Now we are in hostel. There are lots of first year students here in hostel. There are arts,
management and science faculty students staying. When juniors coming management students
trying to show their power on arts students. We had an incident here when we were first years.
We are trying help our juniors. We never allow other faculty seniors to rag our juniors. If any
problem comes we will discuss within our groups and trying to solve them.
Moderator: asking question again. Every one is whispering .
Moderator: let us go to next question.
Moderator : When and where commonly ragging happens? What are the common places ? which
situations helps seniors to do ragging?
Person 1: In our first year of study we got ragging even in classrooms. Before and after every lecture
seniors will come to lecture hall and rag us. In every hour at least 10 minutes seniors will be with us.
Seniors will leave lecture hall after lecturer arrived only. They will make us to sing and they will beat
male students. After 5pm only our English lecture will finish. After that they will ask all the students to
come to benches and rag there. After 5pm benches were the main place to do ragging. They will ask us to
sing and dance there.(smiling)
Person2: Hostel is another main place. He had a senior in our hostel she ask us to greet us daily morning
and evening. (smiling) We have to go to her room daily morning 6am. She will wake up at 7am only.
Until we have to wait there and greet her. After that only we can go to lectures. In evenings we have to go
to her room and greet her then only we can go to our rooms.
Person3: They won't let us study in night. They will arrange meetings at 7 -8pm.
Person4: There is no particular time for ragging. Whenever they bored they will rag.
(everyone laughing)
Whenever we see seniors we will have fear. In canteens also they will rag. They will ask us to buy meals
for them. Every student is come from different economic status. I have seen some students bought them
foods and they don't eat for the whole day or they eat only one time per day. For those students this is
very worst situation.
Someone interfering….
Person 3: in our first year we never go to canteen.
Moderator: can you tell some common places?
Person5: Boarding hostels….

Commonly seniors took junior students to their boarding houses and beat them. For girls they make phone
calls and scold. Sometimes seniors took juniors to the place called "valimadu" and beat them.
Person 2: also they hit boys in bathrooms. If they tell come to bathroom. It means they are going to hit
him.(laughter)
Moderator: Why they hit boys?
Everyone: its their rage… (laughter)
Moderator : who do more ragging??
Person 1: Commonly raggers are person who got more ragging or person who never got ragging.
Moderator: do you think seniors can only rag?
Person 3: 3rd year and 4th year can rag. Direct seniors cant do.
Moderator: university staff?? Others ??.
Person 1: no no.. only seniors . particularly faculty seniors.
Person 2: they was an incident other faculty seniors call our boys and did ragging in our first years. They
told we are your seniors only. They did many things.
Moderator: can someone else from another faculty rag you in hostel?
Everyone: yes.
Person 5: they will do more. They think we are arts faculty. Shut up you are Tamil medium. They are
English medium. Treat us very badly.
Person 2: they will do more ragging.
Moderator: is there any type of students group who are more vulnerable for ragging?
Person6: boys will hit handsome students. Because he can impress juniors.(laughter)
Person 2: handsome boys.
Person 4: They mostly select beautiful students and odd persons.
Person 8: they select students who looks pitty. How to say….?who dress well or talented students.
Person 7:Students who talk more.
Person 1: if we cry also they do more ragging.
Person 5: disobedient also..
Person 1: one time we will meet and tell them don't do this. If there is no change in their activities we
will call them and rag.
Moderator: What will you do when someone giving violent ragging? (explaining question)
Person1: I will stop for sure. If they were our friends we will talk to them and stop it.
Moderator: what will you do?
Person2: With the help of lecturers or students counsellor members we will stop it.
Moderator: can you meet them easily?
Person 2: yeah. We can. They gave instructions. If anything happened how you have to complain?
Moderator: what will you do ? I don't ask normal talks I mean violent ragging?
Person 8: if someone is doing violence he wont be in stable mood. So we wont interfere directly.it will
become problem for us. We will ask help of lecturers, marshall and student counsellors.
Person 1: because already he is not in consciousness. It is not good for us.
Person 2: I will have angry. (laughter)

Person 5: before going to counsellor we can tell his friends. Because if we go to counsellor it will
become problem for him.
Person 1: there are some boys who don't like violence. We can get their help.
Person3: Sometimes it will become problem to us. Sometimes they will stop at that point and later they
will take that student alone to presumable place and do violence. They will tell you have more support. It
will be more vigorous.
Person 4: if someone from first year reports about ragging to marshal or social medias and make an
issue seniors will isolate that student from that batch. Others won't talk to him. We call them anti
raggers.
Student counsellor, student union, president, department head & wardens can take actions against ragger.
Moderator: Do you know anything about posting ragging issues on social Medias?
Person 1: Peoples are eagerly waiting to post news about ragging on social media.
Person 2: They was an incident happened in university. Social media built up like a mass issue.
Actually it's a small problem
Person 3: Some students writing about ragging from a fake account. Yesterday was a cultural day
of Management faculty. Their juniors were kept their books on head and walked with wearing a
saree. That is not a big issue. But someone took a video and upload it on social media. Now it became a big issue in social media.
Moderator: Who do you think will act?
Person 1 Student union
Person 2: Student councilor
Person 3: President
Person 4: Department head
Moderator : Other students, university staff, health staff, wardens, or any other?
Person 8: warden also will act.
Person5: Commonly warden is a Sinhala person. Even if there is a meeting for tamil students warden will inform us and come in.
Moderator: When people talk about the university, what do they say? Do they say the university is a safe or unsafe environment for you?
Person 1: Public thinks in Jaffna university no ragging. No ragging in law faculty.
Because….There is ragging in Law faculty. We do welcome party in 1year. They do in 6months.
There is no much ragging. (other speaker interfering..)
Person : Law students tell there is no ragging. But still there are many female students got affected.(laughter)
Person 7: compare with other universities in Jaffna ragging is less.
Moderator : explain question again?
Person 1: Often we have strikes. Our studies are affected because of it.(Someone interfering)
Person 3: there is no such ragging in Jaffna university to become famous in ragging.
Person 1 continuing: When we came first we got 6months strikes. Strikes wont affect hostel

students much. But students who are staying in hostels have to pay for their rent even if they don't
have studies. Because of this problem students don't like Jaffna university.
Person 2: In other universities ragging is more. But sill they have continuous studies. So students
wont affected more.
Person 3: One of my brother in Peradeniya university. For them they have rules like "yasthuma
…. Yasthumi…" Before registration students have to memorize and tell everything.
Person 4: A book full of rules they will give. Everyone have to memorize and tell them. They should not tell hello. They should tell their code. It is very big paragraph. There is more ragging.
My sister cry daily. They will give one toffee to all students. Everyone have to chew it. They will
give Panadol. One by one everyone have to chew it. There are lot more. More ragging in canteen
only.
Moderator: what parents thinks?
Person 1: My parents are not happy with my education. They told me if you want to study go to
college. Previously I was working in bank. Now they were asking why you left that job. My sister
also studying in University. She used to call them and cry. They ask her why you went there? They are not happy with this University.
Person 2: Its up to parents. My parents were happy. They feel proud that we are studying in Jaffna
university. My parents thinks Jaffna is a cultured area. It's a valuable place. They are very happy.
Person 3: Here studies also well. They want to send their children's to university. That is their aim. I am the first graduate. Our sir also told Jaffna is a cultured place. You can study safely. They
think we are studying in a strong place.
Person4: Inside we have lots of problems. They are thinking it's a cultured place. They don't know the truth. They think if we go to Colombo that environment will change us whether we like
or not. They think Jaffna is safe.
Moderator: Do you have freedom to contact the responsible person to complain about ragging.?
Person 1: There is no problem to contact the responsible person. But we don't do that because if
they expose us it will be problem. If they don't tell our names they won't trust. So they have to
expose us.
Person 5:Anytime we can talk to them freely. But we don't do that. It is the problem inside us.
Moderator: why you don't tell?
Person 2: If we complain to someone maybe it will be problem to us. Affected person will form
a network and start to take revenge.
Moderator: Do you think ragging is reduced now a days?
Person 2: reduced very much.

Person 5: It is very much reduced from our batch. Now a days if we just call them and talk they
will go and complain that we did ragging. It happened for our friend.
Person 6: main reason is population. When we enter 200 seniors for 375 juniors. But now we are
300 juniors are 700. We don't know many of our juniors. With time ragging will vanish.
Person 7: In Jaffna university studies are very length. If we do ragging and get suspended it will
be problem for us. So students don't like to do ragging.
Moderator: Have you attended the introductory program in the university and what does it include?
Person 1: During orientation program they put more A/C. Already we were shivering.(laughter)
Once lecturer finish their talk they will tell your seniors wants to talk with you and they leave. We
will be in fear. We will think what will they do. When will they leave? They will rag boys. They
will scold girls and make scared.
Person 2: Even if they don't talk with us we will get fear.
Person 1: I had an incident. One senior girl scold me for putting kumkum in forehead. She asked
"which boy put kumkum in your forehead?" they will use harsh words. From that I stop putting
kumkum in my whole first year.
Person 3: Here they don't let us put red coloured bindhi. In Jaffna only married women's will put
that. They scold me. But I don't change it.
Moderator: what do you think about introductory program?
Person 1: In introductive program they told ragging is bad then they will send seniors inside.
Person 6: yes they sent.
Person 2: They explained how we have to take our studies during this four years and from whom
we can get our services in university.
Person 3: Every lecturer explained their subject and its importance.
Person 4: We don't know what subject we should take. How the incourse exam will be? How
exam will take place. They will explain everything during this orientation program.
Moderator: ok.. anything else?
Person 5: They have explained about Library section, where to go and what to do.
Person 6: They will take us to whole university. We were going in a line. Our seniors will come
in between. They protect us from getting ragged by other faculty seniors.
Person 7: But for our juniors we didn't do that. Because they don't take us inside. I can't say it's
safe for juniors.
Moderator: Do you think it helps in reducing ragging violence among the students?
Person 8: They introduce all seniors.
Person 1: They will tell you where you should complain for ragging. But we didn't had that guts
to complain.

Person 2: Now a days students are very strong. (laughter) They don't scared to complain. They
have that guts. That is the difference between our batch and juniors. We can tell there is no ragging
now a days.
Moderator: asking question again.
Person 1: Absolutely not. There was a 5days program for us. Main thing is introduction of subjects and selection of subjects. But they can take ragging inside it.
Person 2: In first day they ask a boy sit near me and they beat him. Next day also they did it. I don't want that program so I stop going.
moderator : What do you think should be added to the introductory program?
Person 3: Preference of subject in job requirement is difference in every district. No one knows
about it. If they guide us according to our district it will be good to get a job. In my Mannar district
for 7-8 years there is no students in Tamil subject. We all like Geography. In my bath there are 15
students. It is hard to get a job. So they can give an information about vacancies in every district.
Moderator: what they can add more to reduce ragging?
Person 4: There is no much ragging here.
Person 1: so what to give?
Person 2: They got courage. They know they can complain everywhere. If we just talk they will
complain. So we don't need to give advice.
Person 5: But it is not good for the society. If we told maybe in fear they will wear appropriate
dress from first year. But now there is no such environment. They will face problems when they
go out. So completely we cant stop ragging. No ragging and violent ragging will affect the society.
It should be in proper way. It should help the junior students.
Person 6: We cant manage university without ragging. First year students will form two groups
within them and start to fight. Its better for that.
Moderator(everyone should answer): What do you recommend should be done for the students to respect each other both at home and at the university? ;
Person 1: first I should think that I wont do ragging. If I do ragging I cant stop others. First I should change. Then only I should change others.
Person 2: First we should change the concept of ragging. Here ragging means hitting and scolding.
From ragging we can help them. We can take their problems out and help them. Ragging will be
good if we change the concept of hurting other in the name of ragging instead of helping them.
Person 3: I think before coming to the university we should identify our district juniors and help
them to follow dress code. We should tell them how to behave inside university. If we do that then other students wont rag them. Our siblings only going to come from our area. So we should

help them.
Person 4: There is a group of people who engaged in ragging. We should identify them and help
them to understand student's problems. We should explain them ow much students are struggling
because of ragging. We should help them to understand student's family background. If they think
no one should get hurt because of us then they won't do anything. Then they will change .
Person 5: Instead of giving fear we can give happiness in the form of ragging. We can change ragging from hitting others to taking their skills out. If someone knows singing we can tell them
to sing. It will be fun also. When we have a cultural show we can use those identified pupils. In
those situations ragging will go smoothly. We can develop a rapport between seniors and juniors.
We can also stop violence.
Person 6: Ragging depends on a way of treating others. Some people treat others harshly. Then
that victim will think I got affected so I should give it to someone. In this way only ragging cycle
continues. When a person talk with others smoothly. There won't be much problems. No one will
be hurted. No one will think to show it to others. Ragging will reduce step by step and one day
there is a chance for no ragging.
Moderator: what else you like to recommend?
Person 7: We should stop allowing drunk students inside university.
Moderator : can they attend lectures ?
Person 2: noDrunken students should not attend lecturers in evening. I don't know whether we
can stop usage of alcohol inside university. Because dunk person will show a violent mental state.
They should give very bad punishment. If they do that others will get fear to do that.
Person 8: If there is a meeting surely there will be a fight. They won't come to know what they
are telling in meeting. They come to make problems. So they will drink and come. They will break
all chairs and fans in the auditorium.
Person 1: When there is a final year student talking there will be a 3rd year group to disturb them.
They will drink and come. Their focus will be on disturbing that meeting. Drunken person will
have elated mental state and they will hit juniors and break chair to get attraction and make others
fear.
Person 3: if there is a fight girls will standup and see. So they wanted to show their power to girls.
If it is a boy's university there won't be much problems. Our also also will standup and see their
fight. This is what happen in meetings here.

Students who drinks only problem makers.
Moderator: Do you want to tell more about drinking inside university?
Person 1: We can't conduct a welcome party or any celebration. They will come drunk.
Person 2: For welcome party they will collect 1000rs from every student. Then they allocate a amount for 3rd and final years for alcohol and cigarette. They will come drunk and they don't know
what is happening on that function.
Person 3: But they don't know how much that student suffers to give that money. There are some
students can't continue their education but still they collect money and drink. They make fun.
Person 4: If a senior is beating juniors it means he is drunk. They should hit juniors if they drink.
They will put juniors on a line and hit them.
Person 5: In last meeting one boy telling "sister I am walking in road" and he was walking in grass. I know he was drunk. I don't know he is walking or rolling. He lost his self control.
Person 6: Here there is no control like school. We have student's counsellor. There is no time management. Whenever they can come and go. Some people do studies after 6pm.
Person 1: They will drink here in the name of birthday party. There is no control in hostel. Cleaning staff will normally took those bottles in hostels.
Person 4: they will hit Boys and scold girls. Everyone is affected. If someone got suspended from
studying for drinking inside university whole batch should do a strike to take him inside. Maybe
it will take 2-3 months. Until that everyone's studies got affected because of his drinking habit. He
is doing this from his senior level.
To ban ragging we shouldn't cut branches. We should plug from roots. For that we should stop
drinking and forcing juniors to drink.
Person 7: first year students mostly don't have alcohol drinking habit. They invite juniors to their
homes for meetings and force them to drink. They isolate the students who don't drink from batch.
They use different words to call them. Students will have fear for to get isolated them from batch.
So they also drink.

FGD 11

Person 1: within friends groups…. When we were first years we used to talk about ragging. Then
when we go up 2nd & 3rd years it is not a big issue to talk. But now media is talking about ragging.
Now we talk a lot about it.
Moderator: now a days there is a news a boy post his ragging experiences in facebook and discontinue his studies. do you talk about it? Is he from arts faculty?
Person 2: yes. Arts faculty.
Moderator: do you talk about it?
Person 2: Generally we don't talk about it until it becomes a big news or suicide case. We talk

like why they did it like that. Normally we don't talk about it.
Question: According to you what is ragging? Everyone should answer.
Person 1: Interaction between seniors and juniors.
Person 2: It is a rule to guide newly coming students in a right waay.
Person 3: they can use it in good way. They use this to hurt a person mentally.
Person 4: This opinion will change according to the person you are asking. According to me my
personal opinion is there is a duty for every person in this society to guide. If you are newly enter
university in a free education country like this this society will expect more from you. People thinks you are the server for society. Society don't give that mindset to him. Society told him you
have to get more marks, go to higher places and fulfil your needs. So a person who walked through
these directions should interact with newly coming students. (not clear) but here it is like I m here
for long time. You are coming new. Now I am going to show you my superiority. Now I am going
to show my superiority physically, mentally and psychologically. it is ragging.
Person 5: My opinion is to introduce one to new place showing them the way how to behave and
reducing their capacity of self thinking and decision making from own experiences.
Moderator : now think about the word "pakidivathai" in tamil. What you want to tell about it?
Person 6: There is nothing positive in ragging. Even though I don't have anything superior because
of my superiority complex I am going to under mind others physically and trying growing my superiority. It's an infantile mind set of myself.Things I do to grow my not existing superiority.
Some people will use your race or identity to target you. It is differs personnel to personnels.
Moderator: What you have talked about ragging with your friends?
Person 1 : Recently we talk about Eastern university incident which came on facebook. In Jffna
university no one experienced this kind of incident.
Person 2: In eastern university they ask muslim females to walk in a parade then they hit them by
stagnated dirty water. That video gone viral in social medias.
Person 3: people talk about it in ethnic and religious way. But we talk because there is no incident
happened in Jaffna like that. Still we are opposed for that.
Moderator: what all you have talked?
Person 4: We talk about ragging when our 34th juniors came. We discuss about what all we have
to include in orientation program, which mindset we have to give them. We talked about are you
going to study goal oriented or inquisitive nature oriented? We did a discussion to them. So because of that we talk about ragging. how they have to continue their study here. For that we did
a discussion.

Person 5: we can tell it in different way. He is from medical faculty. In other faculties their way
of handling is different. ……clean shave, dress selection. If the students population is more there
wont be much ragging. May be in other faculties there are ragging. Ragging will depends on how
they handle students.
Person 6: ahh… What he is trying to say is, when I am coming from medical faculty, my opinion
is more polarized. If we go to science or arts faculty situation is different there. That is the place
where students are being hurt.
Moderator: are you going to tell something? You can tell in tamil.
Person 1: tell in tamil.
Everyone is silent.
Moderator : When and where can these types of violence occur?
Person 1: In every situations of life people should have obedience. We should obey someone.
Maybe in office or a shop. You should obey your boss. When a person can't obey he is subject to
face violence.
Moderator: so do you think everyone should be obedient?
Person 2: When a person cant obey his seniors it wont be a big problem that time. Senior will hide them according to his age, knowledge and experience. When he became senior he will think.
If He obey seniors he will come to know what is ragging and what are the rules. He will know they should not spray water over girls. juniors who cant follow rules only becomes violent seniors.
Moderator: here senior only did mistake he spray water over girls. Why do you blame juniors? I think if I get violent ragging I wont give it back to juniors. So I wont do a violent ragging.
Person 1: In my brother's university there is no ragging. Full interactive program. If someone don't attend that program how he will rag juniors?
Moderator : I don't ask you have to rag.
Person 1: To be social towards juniors you should have good interaction with juniors.
Person 2: ragging is happening in the places which are not under tracking.
Moderator : can you tell examples?
Person 2: There are responsible peoples in university. Student counsellor and more. When they
don't function properly or if they give more freedom to students when they find a suitable place
they will rag.
Moderator: what are common places?
Person 3: Rent house, boding house, ground, maybe
Person 2: remote areas.
Person 4: something I like to add to this discussion.Not all ragging become violence. It is depends
on involved personnel's violent tendency. It depends on our social background and excisting philosophy. Some students comes from nicely help up family they will under mind them self to

survive here. Some are independently brought up. They think why do I have to suppress my self?
That time When they were in a group when there is a person cant control his emotions it will lead
them to violence. Then it become as a communal issue. They scold a batch. They use bad words.
They hit. If they could control their emotions it wont become a big problem. Basic is when a individual had violent tendency or he cant control his emothions.
Moderator: ok. Tell the common places where they do ragging?
Person 3: places?
Moderator: yes .. like boarding houses. Places.
Person 1: In some universities there is a separate place for that. There wont be any camera there.
Person 2: mostly secluded areas. It tends to provoke ragging.
Moderator: can you tell commonly in Jaffna?
Person 2: Commonly when they give building, rooms or block to students they wont put camera
there. Those were the places for ragging inside faculty.
Moderator : who are the common perpetrators of ragging?
Person 2: who…
(two peoples joining)
Moderator: can you present your self
(noisy and not clear)
Moderator: explaining question again
Person 1: seniors only. Some seniors have mindset to make fun with junors. They want to do interesting things with juniors.
Person 2: In my personal experience and what I heard from seniors only doing ragging.100% No
chance for staff to do ragging. Some seniors will call you and talk they want to get to know about
you. you feel good when you leave. When i come out from that I will think I had a healthy interaction with him, I was happy and I don't have any problems. Those are the seniors who can
respect others equally and treat others as his siblings. In the same time there is a another kind of
senior. He himself has an inferiority complex. A boy is coming. He is talking clearly. He has lots
of knowledge in his mind. It makes me to feel inferior. I am the senior here. I want to establish my
superiority. That kind of peoples because of their inferiority complex they will have violent tendency they want to establish their superiority they are doing this.
Moderator: explaining question again.
Moderator: so far you mention only seniors. Do other faculty seniors rag you?
Person 2: chances are rare for that.
Person 1: We have that problem in hostel. Commerce and science faculty hostels are being nearby.
When a new batch comes to science faculty, commerce seniors will rag them. They will identify
their seniors within one week. They will rag them during that period.
Moderator: so other faculty senior also can rag you?

Person 1: they don't rag me.
Person 3: when there is a chance they take advantage
Person 2: If this news come out it will become a big issue. Whole faculty will become against another faculty.
Person 3: It will make a communal instinct. Someone ragged medical faculty. Science faculty ragged management faculty. They will form a community.
Person 4: mostly same faculty senior will rag.
Moderator : which faculty you represent?
Some students: AHS
Moderator: which year are you?
Person 6:1st, 2nd, 1st, 1st
Moderator : Quick summery
Moderator : Are there any student groups that are more vulnerable to violence?
Person 1: mostly socially reclusive people they suffer more. student who don't maintain interactive relationship with others in his society or school. Ragging seniors will get provocation
when seeing those juniors. They will think he cant do anything back to me. He cant complain anywhere. They will take it as an advantage. They tag him and hurt him.
Moderator: are you from first year?
Person 7: yes its going to finish.
Moderator: according to you who is affected more?
Person 7: whole 1st years
Person 2: student who don't have a strong background about university. We should tell everyone
what will happen in university. Not everyone know about university. Parents maybe university
graduates. Maybe brother or sister studying in university. They know everything. They will manage every situation. Students comes from different background, no one educated will do whatever seniors ask then only they can survive in university.
Person 3: for some students when they enter university they will know some people. Brother's friend or seniors. Others they don't know where to eat, where I am going and they depends on those lines. He is the spotted one.
Moderator: If anyone notices any type of ragging violence among the students, what do you think people will do? Or what is generally done?
Person 1: When a senior doing violent ragging we have to stop him. Even if it is senior we have to
stop and talk to them.
Person 2: mostly it is their faculty, their junior senior we cant interfere. Student's union will make
problem. They will have fear. But in same university we will notice everyone's behavior. We will
talk to their friends and give warning to their them via students union
Person 3: There sh.ould be an intervention there. When there is a vigorous ragging before it goes
to violence even if you are a junior you can tell your opinion that you are rejecting it. If he is your
senior you will have fear and reluctant. But you should have courage to show your distain. We can
use some path to do that or we have to do directly. But the chances are rare. Need courage to do

that at that point.
Person 4: some in reality people think its not our problem. We have thousands of problem in our
life. They will just look other side and go.
Moderator: who do you think will act? Other students, university staff, health staff, wardens, or any other? (explaining)
Person 1: Victims and seniors.
Person 2: Seniors
Person 3: Only evidence is the victims. So they should tell it out. We don't know how many incidents were happened without knowing others. Important thing is they don't tell it to their parents because they will feel. First they should come out and through senior marshal they should
go to faculty and university.
Moderator: who do you think will act? Other students, university staff, health staff, wardens, or
any other?
Person 4:Person who did have to think. It is a cycle. It may be good or bad. When it seniors should have the duty to change it.
Person 1: truly when we give solution administratively from out it will escalate instead of passify.
To break this cycle interventions should come from the students. we are using is wrong, we should
change it. Then only we can get proper solution for this. When a person got affected seriously administration and marshall should interfere.
Person 3: Should bring the mindset to use good things and avoid harmful things.I have heard in
other universities they said ragging is going in a line to canteen, giving space for seniors and punctuality. But here ragging is hitting. They should be a common rule for ragging.
Person 2: they should standardize (laughter)
Moderator: When people talk about the university, what do they say? Do they say the university is a safe or unsafe environment for you?
Person 5: According to 1st years it is not safe.(laughter)
Moderator: why it is not safe?
Person 5: commonly when we see from things happening in other universities.
Moderator: so it is not safe?
Person 5: yes until 1st year.
Person 2: Now in srilanka in this situation university students are contributing in socio political
state. What I mean to say is discrimination is everywhere. Government office, schools and universities. Violent ragging is a king of discrimination. University is the cream of society. Total
expectation of the society is in university. When a problem comes from that area it becomes a big
issue. I don't tell everything inside university is wrong. I think universities contribute more to make socio political stage. Backlash also here. That contribution is in everywhere. University is
an unsafe environment for others outside university. Because of that only incidents happened inside universities becomes a big issue. (not clear)
Person 3: In every area they decide according to what they heard about university. It may be safe

or unsafe. In my area university is unsafe because first engineering student registered death student
Varaprasanth from my area. So for them it’s not safe. For my area Peradeniya and Ruhunu are unsafe. (unclear)
Moderator: Do you think problem of ragging violence on campus gotten worse, better, or stayed
the same in the last couple of years?
Person 1: now ragging is low.
Person 2: reduced but the fear of society is more. When university students are ring to play a role
in socioeconomic status of srilanka backlash comes from propaganda mechanism so It seems like
an inflated issue.
Moderator: Have you attended the introductory programme in the university and what does it include?
Person 2: I have conduct the introductory program actually. it will give different opinion from others. When we were juniors interactive program is they teach how you should behave in the faculty, how you should use the resources, they orientate faculty, how to interact with superior
students like that. They told how you should behave with lecturers and senior students and how
you should interact with others. We did orientation to our juniors in which way they can use their
mentality and philosophy in university. It is different in every faculty.
Moderator: can I hear from other faculty students?
Person 1: we are medical faculty.
Person 3: question again?
Moderator: Have you attended the introductory programme in the university and what does it include?
Person 3: there as an interaction happened between students.
Person 6: games
Person 4: students population is high around 150 – 200. Not everyone know others. They introduce
everyone and did group events, drama and dance.
Moderator: what do you think about it?
Person 6: they will talk about dress code. It will be there in all universities. Why dress code is it
will give formal neat look. In a new place formal look will give you protection. In Jaffna rowdy
problems also we have. So its good to have dress code.
Moderator: is that good or bad?
Person 5: its essential because now we are in a transformation period. We came to a different place
from where we live for 15 – 20 years. Now I am coming to a new place. Guidance is important in
this time. Its beneficial. In a correct way we should give guidance.
Person 3: For someone’s mistake in some places we can tell the system is fully wrong.
Moderator: Do you think it helps in reducing ragging violence among the students?
Person 2: interactive period give me time to develop relationship and share about violence and discrimination maybe with my senior or my co-students. It gives opportunity to make

conversations to develop relationship to bear your sorrows. If there is ragging without interactive
programs those news cant come out. They don't know where to complain or to whom I can talk
and how to protect my self..
Moderator: :What do you think should be added to the introductory programme in order to reduce
ragging violence?
Person 1: they conduct 3days program if they did it for 5 days it will be good. Students can understand more. They can develop communications. (voice not clear)
Person 2: in some faculties lecturers will be there during interactive period. Every faculty should
follow that. Mixing up with seniors should be an official one in front of lecturers. Then there wont
be any problem.
Person 3: observation will be best than control.
Person 4: In medical faculty lectures will be in front of lecturers. There wont be any problem. If
all faculties follow that there will be interaction and violence will become low.
Person 2: one more thing. During introductory period instead of preaching we should give importance to their self skills. Because people feel defensive. For example in this place some talks
but some feels hesitation. introductive programs should reduce that hesitation from students and
let them feel like all are same
Person 4: interactive program is a fun for us during our first year. Still we think about it and laugh.
Some people sing some dance. Many talents came out.
Person 2: some felt shy after they forced to do we did.
Moderator: What do you think should be added to the introductory programme in order to reduce
ragging violence?
Person 1: we should make students clear about their duties and rights. They should know what
they did for you is wrong, where you can complain. They should not have reluctant and fear. No
need to have fear here. If they understand it they can overcome all side effects.
Person 1: we should teach them their rights. No one can stop you. Like that…..
Moderator: now we are in final question. Everyone should say something.
Person 2: everyone should say something ahh…
Moderator: What do you recommend should be done for the students to respect each other both
at home and at the university?
Person 4: they should change the way they behave. Juniors call them "Anna" and seniors call them
"Thampi". Then everything will change. violence will reduce automatically. They will have affection.
Moderator: what do you think to do to reduce ragging?
Person 1: When I am senior I can give a philosophical idea to my juniors how ragging should be.

Our next generation will come and live here. We need a better system to guide them. Ur society is
expanding. It is our duty to understand and guide juniors. The whole batch should take it as a social
responsibility. How we will finish it as a duty? What is our purpose? What is our target? We should
grow our personality. It will increase our intimacy. It is best to give mindset and philosophy to our
juniors how to move with those basic purposes. If it spreads within juniors it will break the cycle
in one point. This is what I can do.
Person 2: I will stop doing and stop others to do things which I felt violence when I was a juniors.
Person who got hit by seniors will try to give his pain to juniors. We should break it. If we break
once it will reduce.
Person 3: they should arrange a program in which seniors should explain about junior's rights.
Person 1: Senior is not only one year elder person but also whole passed out students. We need all
of their guidance. In 3rd and 4th years when they leave they know whether it should be stopped or
not. Seniors should not just take and give ragging. They should notice what is happening. It is their
duty.
Person 3: everyone should understand the human value of others. Then we can totally stop violence.
Person 4: in our batch everyone is friends. So we should discuss within our batch and let them understand about ragging. Then we can break it.
Person 5: we should avoid the things which hurt us when we got ragged. We should give them
what they need.
Person 2: mostly 1st and 2nd year students have ragging. So 3rd and 4th year students should notice
it and give advice.
Person 3: Discreetly is very low between 1st and 2nd years.so ragging is more violent. To reduce
that tension it should be done officially under administer observation. In this superior students should show more involvement. Because younger generation is following us only. So we should
give more involvement. We should take it to an official standards. Then only expected change will
come. Truly there is an overlap between 1st year and 2nd years. We should reduce the chances to
make them tensed.
Person 4: another problem for violence is, in a same class A/L students one enter university in first
attempt another enter in second attempt. Now this junior cant give respect to his senior friend. There wont be any problem if they see him as a senior only.
Person 5: sometimes personal conflict can turn into communal conflict after they enter university.

To stop that seniors help is very much important.

FGD 12

Moderator: explanation about the study
Moderator: In different type of media we can read about serious type of ragging. You can read
such articles frequently. Do you discuss this with your friends?
Everyone: yes
Moderator :In your group do you sometimes discuss these question and what comments do you
have? Do you think the discussion in media are true/ exaggerated?
Person 1 & 2: very much exaggerated.
Person 1: sometimes they post thing which never happened.
Person 3: sometimes they will talk about the things happened 10 years ago and tell its now. (laughter)
Moderator : ok. Anything else
Person 1: if they got any news about ragging they will tell it is from technology faculty.
Technology faculty will come in their mind for every ragging. They don't try to find out where it
happends, wherever it happens they will put heading that it happens in technology faculty. But
the truth is there is ragging but not this much.
Person 4: compare to other faculties ragging is less here.
Person 1: here only the staffs and security officers are telling ragging is less. But I don't know why when talks about ragging they mean technology faculty.
Moderator: so you think they exaggerate?
Everyone: yeah yeah
Person 1: very much. They will write whatever not happened here. It may happened somewhere
else. They will write it technology faculty.
Moderator : Are there are good ragging also?
Person 5: its not ragging. Its guidance. Like we can show our talents. Singing and dancing.
(Everyone talking not clear)
Person 6: when we do something with our wish it also comes under ragging. then they ask them
they will also tell the same.
Moderator : then what is serious ragging?
Person 1: we don't know. We never experience it.
Moderator: don't you?
Everyone : no.
Moderator : When and where can these types of violence occur?
Person 6: mostly ground.
Person 5: ground.
Moderator: ok what else?
Person 3: in our faculty before university starts. After it starts there wont be any ragging. Before
admission. We had call ragging.
Moderator: sorry what is that?

Person 2: after 2nd for they will find who got selected from which area. They will ask our details
ask us to call in nights and asked to sing or if we do any mistake we have to write bad words many pages and have to send then a picture of it. Asked to tell all district seniors.
Moderator: ok. Do you know any places inside university?
Person 2: no chances in hostel. Its different place. Common in ground and some seniors will take
juniors to their boarding and do. Second years will stay out. So they will call their juniors out.
Moderator: ok. According to what we have discussed now who are the possible raggers?
Person 1: who means?
Probe; Are they senior students, others at the university, people you know/don't know?
Everyone: seniors….
Moderator: within seniors?
Person 1: second years are the most
Moderator: which means your direct seniors?
Person 1: yes. According to our faculty ragging among tamil and muslims are rare. Likewise we
dont rag our juniors. Its more within sinhalese.
Person 2: they wont tell out.
Person 1: even if we ask their juniors they wont tell. There wont be any ragging complains within tamil students. You can ask our staffs.
Moderator: female or male which senior is doing severe ragging?
Person 2: we don't know. They also do.
Moderator: do other faculty people, staffs any unknown peoples do ragging?
Everyone: nothing like that we have.
Moderator: Are there any student groups that are more vulnerable to violence?
Person 3: innocent
Person 2: active people
Person 5: people don't know this place
Person 6: poor students.
Person 5: active students who can take responsibilities.
Moderator: is there any differences in ragging between tamils, Sinhalese and muslims?
Person 4: Sinhalese do severe ragging. We have to do welcome party after ragging. Sinhalese will say you don't rag your juniors then how you will do welcome party with our juniors. You have to do ragging to do welcome party with us. Tamil seniors don't rag. They will ask to follow
dresscode. There is no ragging other than that.
Moderator: is there any kind of differences in ragging between students come from city and rural?
Person 2: no
Moderator: If anyone notices any type of ragging violence among the students, what do you think people will do? Or what is generally done?
Person 2: do you mean our batch?
Moderator: yes
Person 2: if it is tamil I will go and ask if it is Sinhalese when this case go to administration juniors will tell seniors didn't do that. So we don't care Sinhala people. We don't let tamil people
to do ragging.
Moderator: who are the responsible people to stop ragging here?
Everyone : yes

Person 2: there is a crew.
Moderator: who all are there?
Person 4: student counsellors, marshall
Moderator: do you have marshall?
Person 4: now we don't have separate marshall. From Jaffna university one person come frequently. In future we will get a separate marshall.
Moderator: ok anything else?
Person 2: but illegally unofficially they told everyone like securities and staffs should complain
if there is ragging took place anywhere.
Moderator: do wardens, health staffs and staffs will stop ragging?
Person 2 : yes.
Person 3: lecturers also will help.
Person 2: here there is no ragging.
Moderator : When people talk about the university, what do they say? Do they say the university
is a safe or unsafe environment for you?
Person 5: Jaffna is safe only.
Person 6: no language issues.
Moderator: ok ahhh… do they feel it is safe.
Everyone: yes.
Moderator: how your parents and relatives get to know about university issues?
Person 1: victims will tell their parents. They saw news.
Moderator : Do you think problem of ragging violence on campus gotten worse, better, or stayed
the same in the last couple of years?
Person 1: reduced
Person 2: its reduced now.
Person 5: its reduced than last year.
Moderator: do you think students unions helps to maintain ragging in university?
Person 5: we don't have an official students union. only 3 batch we have. Only for the functions
we have a union.
Moderator: ohhh so you don't have a union.
Person 5: we need 4 batches to make a union.
Moderator: ahhh ok.
Person 5: we cant register now.
Moderator: you peoples are recently separated from your house for the first time. Here do you face the problem of getting addicted to alcohol or drugs?
Person 2: no.
Moderator : do any seniors force their juniors to drink? Anything like that?
Person 2: no
Person 1: nothing like that according to our knowledge.
Moderator :Have you attended the introductory programme in the university?
Everyone : yes
Moderator: what does it include?
Person 1: first of all they talk about ragging. They told doing and getting ragging is a crime. Then something about subjects.
Moderator: ok. Ahhh what was your senior's contribution on your orientation programme? (unclear)

Person 2: they did a small program.
Moderator: ok what do you think about that programme? Do you think it helps in reducing ragging violence among the students?
Person 5: it helps us to know about others
Person 6: yes.
Moderator: aahhhh…Do you think it helps in reducing ragging violence among the students?
Person 3: ohhh they gave some address and mobile numbers to contact for help. Then they gave
web address to complain.
Person 2: there only they told where you can inform.
Person 5: we can inform to UGC
Person 2: they told where all we can inform and how to do that.
Moderator : What do you think should be added to the introductory programme in order to reduce ragging violence at your university?
Person 1: you have to inform the students before they come to university. Because after 2nd form
they find out students are they told them not to complain about ragging.
Moderator : ok mmmm…Is there anything you would like to have in your university that is not
there yet? Such as sport facilities, meeting places, mentors, counsellors, health clinic?
Person 1: we need lots of thing.
Person 2: many things.
Person 3: we need wifi in university. Important.
Person 1: morning we will have lectures there. Then to practical we have to walk here. Agin we
have to go back.
(unclear)
Person 4: that is they build a building for us. It is not yet completed. Because of fund issues works are going very slow. They said they will finish before last december. Then they told last
February. Now they use agriculture's and engineering's lecture halls. For practical we have to come here. Again we have to walk back. Within one hour we have to eat and go back. In between there are 2 gates. If hey open it, it will be little near. But they are not doing that.
Person 2: Even if they build faculty it is very far to come from hostel.
Person 4: leave hostel. If we go to hostel we will be there till evening.
Person 2: canteen. We took food from canteen. We can get foods from there but we cant eat. It
is very dusty.
Person 4: its ok for us. But there is no vehicle for lecturers. Some times they will come by walking. From our faculty. Sometimes they will come 15 minutes late.
Moderator: ok what all you need?
Person 5: ground.
Person 1: previously we use this ground. But now they stop it.
Moderator : What do you recommend should be done for the students to respect each other both
at home and at the university?
Person 3: if we have functions...
Person 2: if we know each other only we can respect. Now we don't know students. We don't know them at least just to say hi..

Person 1: frequent discussions about ragging within students. If we expose in public meetings….
Moderator: ok what else?
Person 2: we can go to trips.

FGD 13

Moderator: According to you what is ragging?
Person 1: they don't know what is ragging. Here ragging is hitting and scolding via phone call. In
tamil we call it as "Pakidivathai". Seniors and juniors should have good relationship. but here they
spoil that relationship and they do ragging by hitting and kicking juniors to make them get fear
when juniors sew seniors.
Moderator: tell me what do you mean by ragging?
Person 2;: really they do ragging bring senior-junior understanding. But some people use it wrongly.
Moderator: what they wanted to show?
Person 2: They show their seniority in unwanted place. They should show it in studies or in a passion. Instead of that they rag now and when we go out they will work under us. Ragging is when
a person who don't have any ability use seniority to show him huge and hurt others.
Moderator : In your group do you sometimes discuss about ragging?
Person 1: Yes
Moderator: what things do you talk?
Person 1: we will talk only what we can talk with them. We don't rag now.
Person 2: one day hey ask to wear dhothi little higher. We talk about that.
Person 3: they were asked to put their books over head. JVP news published that. We talk about
that. One arts boy discontinue his study because of ragging. It was a big topic.
Moderator: When and where can these types of violence occur?
Person 1: whenever there is no lecturers, seniors will rag.
Moderator: mention the most common places..
Person 2: hitting will not be there inside university. Outside university in room ragging only they
will hit.
Person 3: in one of the seniors room
Person 1: in hostel
Moderator : tell me specific places like grounds class rooms…
Person 4: mostly they will hit in room ragging. Specially their district seniors will hit. (laughter)
Moderator: instead of helping do your seniors hitting you?
Person 2: our district seniors will hit first then they will help.
Person 3: according to Jaffna they don't need to help. It's a Tamil area. We will do everything.
Mainly they will hit you.
Person 5: in Jaffna University we never hit others. After our batch no much ragging. -
Person 6: now its reducing little by little. In all districts its reducing.

Person 7: (unclear voice)
Moderator: when they hit?
Person 1: after lecturers if we get 2hours or 3 hours gap they will do
Person 5: no ragging inside university
Moderator: can you tell me very common places ?
Person 2: if we cross a senior on bench surely they will rag.
Moderator: is there any special bench for seniors?
Person 3: there are 2 benches. For seniors and final years.
Person 4: if juniors cross that when more seniors sitting they wont hit they will ask us to sing.
Moderator : According to what we have discussed now who are the possible perpetrators?
Person 1: whoever got ragged more will rag others. But not everyone like that.
Moderator: do every senior engaged in ragging?
Person 2: not seniors means they will rag you. some people who got ragged more wants to return
them.
Person 3: our seniors hit us. We will give it to our juniors. But if we stop hitting them next batch
also wont hit their juniors. If we hit it will continue. (laughter)
Moderator : do any other persons other than your seniors can rag you?
Person 1: no
Moderator: demos? Lecturers? workers?
Person 2: no but they will encourage (laughter)
Person 3: no we don't know about it.
Person 4: we don't know.
Moderator: did you experienced it?
Person 1: no it never happened to us.
Moderator: Are there any student groups that are more vulnerable to violence?
Person 1: if any strong juniors or adamant juniors.
Person 2: if they got different slang of talking. Rude talking.
Person 3: respectful.
Moderator: soft students?
Person 2: no
Person 4: if we got a junior whose big brother was our senior. He is our enemy now.
Moderator: why?
Person 4: We will return everything we got from his brother to him.
Moderator : can you describe common characteristics of those juniors? Looks? Activities?
Person 2: when they have different hairstyle. (laughter)
Person 1: they should follow dress code. If they get angry when we call them to talk.
Moderator: what is wrong with dress code?
Person 1: dress code…. Open shirt buttons and very slim fit jeans.
Person 3: when they don't looks like first years.
Moderator: what is the dress code for juniors?
Person 3: They should wear stretched shirts. plain shirts tugged in, without belt and shoes.
Moderator: why because of dress code it goes violent?
Person 2: why dress code matter go violent is we will tell them 2 days. If they don't obey we will
rag on third day.
Person 3: when everyone is wearing same dress code one will come differently. It means we wanted to show him superiorly. Because of it we took a common thought and hit him.
Moderator: he may don't have a dress to wear.

Person 4: we know how poor student will come. We know the difference when they don't follow
dress code because of poverty. Students who wear High level dresses only being targeted.
Moderator: do you mean wearing stylish dresses?
Person 4: yes
Moderator : why do you target them?
Person 5: we have to give equality to everyone. When a single person come oddly we have to care
that. First years have dress code. Thy have to follow it. We don't know who create it.
Moderator : is that a good thing?
Person 6: when they follow it , there wont be any differrence between rich and poor.
Moderator: If anyone notices any type of ragging violence among the students, what do you think people will do? Or what is generally done?
Person 1: if our batch did we will join them. When other batch doing we will feel pity. (laughter)
Person 2: not like that .sometimes we will tell them to stop.
Person 3: sometimes we will take photos and publish them.
Moderator : will you stop even if it is your senior?
Person 2: seniors means they came before us. That's all.
Person 4: we wont take any actions when final years doing ragging.
Moderator: do you stop it indirectly?
Person 5: we can stop it but we didn't do that. If they got suspense their degree will go back one
year. sometimes it will spoil his life.
Moderator : why you don't stop your seniors?
Person 2: it will make problem between us. Now we are seniors.
Moderator: so will you stop now?
Person 3: if they talk normally we cant do anything.
Moderator: if it goes violent?
Person 3: we will stop.
Moderator : what will you do to stop?
Person 2: they will listen if we tell. Sometimes it will become as a fight.
Moderator: what if fight comes?
Person 2: there will be a fight between us and our friends.
Moderator: ok
Person 3: junior will escape
Moderator: who do you think will act? Other students, university staff, health staff, wardens, or
any other?
Person 1: Lecturers, dean, Marshall, Student counsellor
Moderator: do wardens help?
Person 2 : in hostels wardens can stop it.
Moderator: university staff, nonacademic staff do they involved?
Person 3: they wont help. They will be in administration room.
Person 4: as a staff marshall will help.
Moderator: only marshall?
Person 4: he is responsible for ragging.
Person 2: if they put CCTV cameras everywhere there wont be any ragging.
Moderator : When people talk about the university, what do they say? Do they say the university

is a safe or unsafe environment for you?
Person 5: they will tell us to go to Jaffna.
Person 1: compare to other universities in Jaffna university there is no ragging.
Person 2 : instead of university when we look district unions ragging is more when we enter. In
Vavuniya district union ragging is more when we come. Now it is reduced.
Person 6: we don't know about other faculties. Arts and management faculties are like this.
Moderator : what is your parent's view?
Person 1 : we don't talk about ragging with our family. So they think its safe.
Moderator: they think its safe? Do they talk about ragging with you?
Person 1: they will talk if we told.
Person 1: my family knows.
Person 2: they would have known if anyone studied previously.
Moderator: do they like now? Do they thinks you are safe now?
Person 5: yes they think.
Person 3 : they had fear when I was studying in first year. Now its ok.
Person 4: they got fear when they read news about other students.
Person 2 : during first years we had fear to complain. If they are closed to seniors they will tell.
Again when we think about seniors we had fear. Maybe they will do anything.
Moderator : can you complain about ragging? Can you easily contact them?
Person1 : now ok but its not same in first year. We had fear. Maybe they can tell to seniors.
Moderator: I am asking how is your relationship with your lecturers?
Person 2: now it is ok.
Person 5: we don't go because of the fear we ha inside.
Moderator : do they upload news in medias?
Person 1: yes true. maybe students can do
Person 2 : they will take photos and upload on facebook from university itself. We don't know
who is doing that. But its happening.
Moderator: do you know them?
Person 3:no no. if they have any relatives in media they will took pictures and send it.
Person4 : now everyone have facebook. So all are media persons.
Moderator : do 3rd and final year students attend meetings drunk and hit you?
Person 2: in hostels
Moderator: in lecture halls?
Person 1 : not in lecture hall. It is there in hostels.
Person 2: it will happen in any functions. Welcome party, marcose, sports.
Moderator : do they force juniors to drink alcohol?
Person 1: we never experience it. But we heard that it happened.
Person 3: Not inside university
Moderator : do you experience it outside university?
Person 3: we heard. We never experience it.
Moderator: do they attend meetings after drunk?
Person 2: not everyone like that. When 10 people arrange a meeting 1 or 2 will come drunk.
Person 3: when they arrange ragging in rooms within a batch seniors 1 or 2 will be drunk.
Moderator : do they drunk and gave violent ragging?
Person 2: no no. there will be one non drinker. He will control them.
Moderator: do you celebrate Midnight Parties?
Person 1: no no. we don't have that.

Moderator: birthday perties?
Person 1: no. we will celebrate birthday parties within our batch. We will cut cake. Nothing else.
Moderator : we got some points from previous interviews. They said after 5pm boys will be drunk
in stone benches and they will make problems with girls. Is that true?
Person 4: nothing like that.
Moderator : Do you think problem of ragging violence on campus gotten worse, better, or stayed
the same in the last couple of years?
Person 1: its reducing.
Person 2: its not the same like our first year. Very much reduced.
Person 2 : it will become no ragging in future.
Moderator: ok. There wont be any ragging in university. What about room ragging?
Person 3: they do district wise.
Moderator: it wont change.
Person 4: yes. It wont change. if one batch stops it wont continue.
Person 5: not every districts are same. In Jaffna district no room ragging. People comes from outsides do that.
Person 4: outsiders only stay in rooms. So
Moderator: not in Jaffna district?
Person 4: yes
Person 6: they wanted to take revenge. They hit me so I will hit. That kind of mentality.
Moderator: do they hit to take revenge?
Person 6: yes I want to give back.(laughter)
Person 2: if one batch stops other batches don't know about it.
Person 3: they can hit the person who hit them. But they are hitting others.
Moderator: Have you attended the introductory programme in the university and what does it include?
Person 1: they do interaction. They explain about university. They will talk about ragging and actions have to be taken for ragging.
Person 2: to whom we have to complain about ragging…..
Person 3: what are the facilities available in this university. Like that they will tell everything. It
happends for one week.
Moderator : Do you think it helps in reducing ragging violence among the students?
Person 1 : yes
Person 2: they gave phone numbers of responsible personalities and person who can took action
against ragging.
Person 3 : they gave introduction of responsible personalities.
Person 4: I ran away from first year after they hit me. (laughter)
Moderator : What do you think should be added to the introductory programme in order to reduce
ragging violence?
Person 5: cant do anything.
Person 1 : first years have fear. They wont tell about ragging to anyone. Even if we ask who did
ragging they wont tell.
Moderator: What do you think should be added?

Person 1 : by taking actions district wise only we can stop ragging.
Moderator: what kind of actions?
Person 4 : we should dissolve district union and form a common union. Districts union are doing
room ragging.
Moderator: do they have unions for every district?
Person 4: Vavuniya, Jaffna, Mannar
Person 6 : some unions are registered in university. Some are not.
Person 5 : unions should be registered and they should have a responsible person. Then they wont
do ragging.
Moderator: where you have to register?
Person 1 : when we form a union we should register it in university and come under a formal union. MCSE , CSU are registered unions. Except it every district Vavuniya, Vadamaratchi, Thenmaratchi form a union for them.
Person 2 : they wanted to lead their district students.
Person 3 : commonly we don't have ragging. We have ragging only district wise.
Person 4 : we can have ragging. But they should not hit. They can advice juniors. But here ragging
is only hitting.
Person 5: by hitting only they gain respect
Moderator: what is the contribution of district union in ragging?
Person 1: full contributions comes from district union only.
Person 2: our district seniors are our problem. We don't have any problem from Jaffna seniors.
Person 3 : ragging happens in faculty wise. In management faculty their district management seniors only rag you. Its not in hostel.
Moderator: can other faculty seniors rag you?
Person 4 : other faculty seniors cant do anything. Our seniors wont allow other faculty seniors to talk or do rag.
Person 5 : they was a problem. They hit another faculty because there is no difference in dress code.
Person 2 : other faculty seniors should not hit our juniors. thats why we have a dress code. Only
we will hit our juniors. (laughter)
Moderator : What do you recommend should be done for the students to respect each other both
at home and at the university?
Person 6: they will help you in getting funds.
Person 1 : there were ragging. Also they will help.
Person 2:Only one day they will hit you
Moderator: only one day?
Person 2: yes. After that when someone rag if I tell leave him, he will let him go. One day only we
will hit.
Person 3 : do they hit you only one time? Don't you remember they hit you in my room?
Person 2: its only one day.
Moderator: do they call you and hit after A/L exams over?
Person 2: they will hit soon after A/L result came. They will hit commonly.
Moderator: how they reach you?

Person 4: they will get one phone number through that thy will contact everyone.
Person 5: senior students from our school will get to know who is coming .
Person 6: not every union do that. Only Vavuniya union doing that.
Person 5: do something and dissolve our union.
Person 2: don't dissolve union. Stop ragging.
Person 5: management don't need a union. You can dissolve it.
(unclear)
Person 3: during first years everyone will hit you. 2nd year, 3rd year and final year. Only Vavuniya
like that.
Moderator: what if you don't go to ragging?
Person 2: fear in our mind only let us go.
Person 5: they will scold you. Really I didn't go. They scold me. I gave phone to my parents.
Person 2: if you don't go later you cant attend any functions like welcome party.
Person 5: they told me not to come. I don't mind it. I didn't go.
Moderator: how you can stop this cycle of ragging?
Person 2: for that we should not do anything.
Person 3: if one batch stop ragging. Everyone will stop.
Moderator: why they are not stopping?
Person 2: when we were in first years we thought we should not rag. But when we got seniority
we wanted to hit.
Person 3: some people wanted to gain respect by hitting.
Person 2: if we ask them to stop or juniors now they will tell when you hit me we accept it. Like
that I will hit.
Person 5: it will be a problem for the person who gave ragging and person who tried to stop.
Moderator: have you done anything so far to stop ragging?
Person 1: no. we didn't do anything.
Person 4 : we stop giving ragging. So now we don't have much ragging in our faculty.
Person 2: now ragging is reduced very much. Really we don't have aging now.
Person 1: we have to talk to the juniors.
Person 6: we should change their mentality.
Person 5: if you ask first years they will tell you more
Moderator: no we are interviewing every years.
Person 2: what they told do they wanted to give ragging?
Person 3: now they will tell they want to stop it.
Person 5: you can ask them not to go to room ragging. You can tell if you went we will cancel your
admission. First year students will have fear for everything. They don't know about university. If
you advise them during orientation it will be helpful.
Moderator: do you think first year students can stop ragging?
Person 5: yes. If they don't go to room ragging.
Person 2: if they don't cooperate seniors cant do anything.
Person 3: administration should help to stop. If they give suspension to 5 people then they wont
do ragging.
Person 2: if we don't do anything they wont respect us.

Person 3: they should obey us. They don’t need to give respect. If we call they should come to talk.
Person 5: we ask only to stop hitting.
Moderator: do you think if you stop hitting they wont give respect to you?
Person 4: no. its not means only ragging brings respect. Juniors will talk to you only during first
years. In 2nd years they wont talk to you.
Moderator: what will happen if you stop ragging?
Person 2: they will started to dress as their wish. We wont help them
Person 5: during events when seniors show their performance juniors will get respect. By hitting
only we don’t get respect. By showing our skills we can bring respect.

FGD 14

Moderator: Introduction

Moderator: What is ragging to you? What do people say ragging is or what come to your mind when you mention ragging?

Some whispering between the participants.

Person 1: Hmm…… Physically harming or mentally harming?

Moderator: What do you think?

Person 1: Hmm…Can only complain it’s ragging if it goes to the physical level.

Moderator: What do the others opinions? Do you agree or disagree?

Speaks very softly and inaudible …….

Person 2: (speaks softly) Physically or mentally harassing another person is ragging

Moderator: What do the others think, what are your thoughts, what to your parents say? You must be having some idea as university students…..

Person 3: If someone is going something that causes physical or mental harm to another person

Inaudible whispering

Moderator: Is it done with or without consent?

Everyone together: without consent

Moderator: Is ragging always bad or there also good parts?

Person 1: Sometimes can be good (others agreeing with her and talking at the same time), sometimes can be very bad (another person talking at the same time)

Person 4: If you say ragging………

several talking ppl at once

Person 4: oh I don't know…… giggles
Person 1: according to the action taken……… giggles

Moderator: please feel free to express your ideas, there's no right or wrong answer

Person 1: Sometimes ragging is like an orientation…… to get to know people….. it's good like that…… to make connections….. but after you make the connections…. If the seniors enforce….. like to get their work done……. Like trying to ask for favors every time……..If they have a project they hand it over to us, which is a real nuisance.

Moderator: Does ragging continue even after the orientation?

Everyone agrees

Person 2: Sometimes yes……….inaudible (several speaking at the same time)

Person: Some people…….. inaudible (several speaking at the same time)

Person 1: Not violence per say……. But says verbally don't do this…… you can't do this yet….. can't go to study halls… like things

Moderator: I have heard that there are some written rules to be followed, I don't know if it applies to the medical faculty. Is there something like that?

Person 1: In the beginning there is a way to talk……..

Others agree

Moderator: What about the dress code?

Everyone talking together

Person 2: Other faculties, some universities have it…… (two people talking together)

Person 1: Here the management, Arts faculties have it (others agreeing)…….. our medical faculty doesn't have it….. As far as I know none of the medical faculties in Sri Lanka make students wear "Cheetha" dresses. (another person agrees)

Moderator: What else is there in ragging? In other faculties, in other universities? I have heard different things…..Like troubling girls?

Person 3: There are some things……. Like personally….. they select a girl and call it ragging and harass her unnecessarily.

Moderator: Have you heard of anything like that happening here?

Person 4: Here we haven't heard anything like that (others agree)

Person 5: we also only heard thing from the news

Moderator: So not in the medical faculty, but what about other faculties?

Everyone agrees

Everyone talking softly together…… management faculty

Person 1: There is a little bit in the management faculty………inaudible

Moderator: What about other faculties?

Everyone mumbling together

Person 1: we had the most connections with the management faculty…… we shared the hostel with the girls from Management faculty……

Person 3: In the hostel…….

Person 4: Yes….. we were in the hostel with them

Person 1: their seniors used to come in the morning…….. have to walk in line…….

Person 2 : Seniors come to the hostels…….

Person: Science…….. seniors

Everyone mumbling…… comes to the hostels

Moderator: Who are these seniors? Girls or boys?

Person 1: Boys for the boys and girls for girls

Moderator: Do the boy harass girls and girls harass boys?

Person 5: We haven't seen

Person 1: No….. we haven't seen…. Have we?

Others…. No we haven't

Person 1: they talk but……

Person 2: yes.. they talk

Person 1: They things till the girls cry sometimes……. But it's only verbal……I don't think they do anything more than that
Moderator: so you haven't been ragged by boys?

Mumbling…….. no

Moderator: So only girls for girls?

Several people talking together

Person 2: No…….. it's not like that…..

Person 4: During the orientation……..

Person 1 : there is no rule like that……..

Person 4: Talk during the orientation (person 3 talking at the same time)……but it's not ragging

Several people talking together……. It's not a rag….. asking the name, where your from….

giggling

Person 3: Things like that…..

Person 1: They just talk in the beginning…… to sing……….

Person 6: things like that

Moderator: but nothing harmful? Do students from other faculties harass you?

Everyone…….no

Person 1: No……. not from other faculties….not to us

Others giggle

Moderator: so your lucky your from the medical faculty

Everyone giggles and agrees

Person 4: That's true……. And our faculty is also separate….. away from the main university

Moderator: Where does ragging occur mostly? Inside the faculty or outside the faculty or at the play grounds or where?

Everyone together…….the hostel

Person 2: The grounds
Person 1: Hotel…….. it happens….. during weekends and all

Everyone talking together inaudible

Person 3: different from faculty to faculty…… happens in the hostels and the grounds

Person 4 : Management ones are taken to the seniors boarding's and they do it…….some say

Moderator: That means places outside the campus?

Person 4: There are seniors who live outside

Moderator: So they can't take a whole batch……..?

Several talking together……take several students……. Select a few

Moderator: What are the times these things occur? Are there specific times?

Everyone giggles….. No not really…….

Person 1: For the females…….of course……………… during afternoon's……. when the students come to campus……. Don't come to the hostel….. don't do a lot for the girls

Moderator: the senior girls have not come to the hostel and told you anything?

Person 1: No not to us……..never tell us anything directly…… comes from the "aiyas" (senior brothers)…….. the senior girls tell the senior boys, and they tell the boys from their batch…. And they tell us…..

Everyone giggling

Moderator? From other faculties?

Person 2: no……in our faculty……..

Person 3: not a rag…….

Person 6: the seniority……..

Person 5: make a small interference……..

Moderator: I didn't understand…… explain it to me

Everyone giggling

Person 3: if they want us to do something…….

Person 4: if they want to get something done….. it will go to their batch…… then it will come to us across their batch…

Person 2: the senior girls message will go to the boys of our batch…….

Person 3: Now……..

Person 1: Even if the senior girls are face to face with us……. The won't tell us directly…. Even if it's some mistake we did to them….. for example, like closing the door in the study hall on them……..or was in the study hall…… or something….. it will definitely first go to the boys and then come to us

Everyone giggles

Moderator: Does this happen in other faculties also?

Person 1: I don't think so……..don't think it happens in Jaffna……..

Person 2: The girls………..

Person 1: The science faculty…….. we don't know…… But in our faculty don't say anything directly.

Moderator: Is it only the seniors who rag, or do the others like demonstrators also rag?

Person 3: No no……..nobody else…..

Moderator: Is it only the immediate senior batch or do seniors from other batches also rag you?

Everyone together………all of them

Person 4: I think the largest involvement is from the most senior or 4th year batch……final year

Everyone giggling

Moderator: I have heard that no matter how senior you become the batch above them will harass them…… or there are designated places for students from each year….

Person 1:……… not seating………..

Person 3: No nothing like that

Several speaking together ………. But the study hall seats a prioritized for the seniors

Person 2: senior priority must always be given…….
Person 3: Even in the cafeteria

Person 1: we are not allowed into the study hall……..giggles……

Moderator: I have heard that in some faculties, they are not even allowed to go to the bathroom…… Is that true?

Everyone together……. No…..no……nothing like that

Person 4: No there's nothing like that in Jaffna I think……..

Everyone together: Nothing like that

Moderator: Not even in other faculties? Don't allow to go to the bathroom and don't allow to go to change their pads.

Person 1: Nothing like that

Person 5: No

Person 1: We were in the kokuvil hostel with Arts students……….there are a little different ………but there was nothing like that

Person 6: there were no first year's there

Person 1: there were only 3rd years and 4th years

Person 2: They were seniors

Person 3: We didn't have a problem with them

Person 4: I don't think they caused problems for their juniors also

Moderator: Is there a group of students who is more vulnerable to ragging?

Person 3: No

Moderator: Due to their ethnicity, religion, economic state or caste?

Lot of people speaking together: No

Person 4: Not because of race or religion but can be due to their behavior

Person 1: agrees

Moderator: So never because of ethnicity or caste?

Person 2: No, no

Everyone agrees

Moderator: is this only in the medical faculty or in all faculties?

Person 5: No in it's the same in all faculties

Person 6: agrees

Person 1: Even if they rag it's done separately…..

Person 4: Sinhalese are ragged by Sinhalese seniors

Moderator: I have heard that Tamil seniors sometimes rag Sinhalese juniors…….is that true?

Few people talking at the same time; this happens at the orientation…….they just talk

Person 1: they don't call again and do anything….. just the first chance

Moderator: What do they ask?

Person 5: just about ourselves

Person 6: Like our seniors they also ask us information about ourselves

Person 1: They don't give us work or anything like that…… just ask questions

Moderator: when someone is getting ragged in an unusual way does anyone stop it?

Person 3: What do you mean?

Moderator if some is getting ragged unpleasantly, does anyone stop it? Or they mind their own business?

Person 1: No……..isn't it……

Person 3: Even during our orientation our pre-clinical coordinator is there to make sure nothing happens.

Moderator: in other cases? If someone is abusing a junior physically or something like that?

Person 2: A senior above the person who is ragging the junior will stop it. But the other juniors can't stop it.

Moderator: Who people complain to anyone? For example, like counselors, wardens, marshals, deans, lecturers?

Person 1: yes, they complain

Person 4: there was a problem in our junior batch and they complained to the dean.

Several talking together…….. they brought the wardens……. (not clear) and asked them……

Moderator: So, there are time that students complain to someone.

Moderator: What did people say when they heard your coming to the Jaffna university? What did your parents say? Were they afraid to send you? Do they say it's a safe place?

All together: (smiling) …… It’s a safe place

Moderator: What do they say about ragging? That it’s less or more? For example, less or more than Peradeniya?

All of them giggling ……… (not clear)…….. it’s less here…… Peradeniya is more …..

Moderator: Do they say ragging is less in Jaffna University?

All giggling

Person 2: Less

Person 6: Say there isn’t

Person 5: very less

Everyone agreeing

Person 4: Medical faculty has no ragging

Person 3: medical faculty doesn’t

Person 1: Less in the whole of Jaffna university

Person 5: They call us “aala” (person who doesn’t like ragging)

Everyone agreeing and giggling

Person: Jaffna university has less ragging

Moderator: So, the whole Jaffna university is “aala”?!

Everyone giggling and agreeing

Moderator: What do they say about the education at Jaffna University?

Several people talking together: they say the education is good

Giggling and talking together….. (can’t understand)

Person 2: say it’s too good…….. giggling

Moderator: Why is that? Why do they say that? Because you can’t understand?

Everyone giggling

Person 2: No it’s not like that

Person 3: They give the main priority for studies

Moderator: Oh, so they focus a lot on studies and not on other aspects…

Everyone agrees

Person 4: There are other things not there isn't, but prioritize education

Person 6: But priority is always for education

Moderator: So you don't have sports and other activities?

Giggling

Person 2: There is…….

Person 5: Only after lectures, can't cut lectures and in the evenings………

Person 1: Even the "Medicos week" continued for a month because the events were only after lectures in the evening……. Giggling

Moderator: So the students and the lectures focus only on studies?

Everyone giggles

Person 1: No the lecturers are the ones, students don't mind doing sports

Moderator: Since you come from far places, do you go home for weekends? Or is it difficult to go home for weekends?

Several talking together………it's difficult to go home
Person 2: Can only go during vacations

Person 3: Can't cut the lectures

Person 4: Can only finish the lectures and go home……

Several talking together and inaudible

Person 5: Or must finish lectures and go on a Friday, and stay Friday and Saturday and come back on Sunday evening……

Person 6: Otherwise must cut lectures and go …….and then you get punished….

Everyone giggling

Moderator: Are all lectures compulsory?

Person 2: They say all lectures are not compulsory

Everyone speaking and giggling together

Person 3: Lecturers get angry……

Person 1: They get angry……. And then don't give the tutorials…. only give to the students who came for the lectures…..

Person 4: You need 80% attendance for the tutorials

Moderator: Ultimately everyone attends irrespective of it being compulsory. Does this only happen in the medical faculty?

Person 1: only medical faculty

Person 3: No right?........

Everyone speaking among themselves (inaudible)

Several talking: not all lectures are compulsory

Moderator: So, the students from other faculties have more free time?

Person 2: Yes

Person 4: They have enough free time

Everyone agreeing

Giggling

Moderator: What do they do in their free time? If they don't have much studies and don't go home also, what do they do?

Several people talking together……. (inaudible)

Person 3: They stay in the hostel and sleep……giggling

Moderator: Do you think ragging is more because the students from other faculties have more free time?

Everyone agrees

Several people talking together……inaudible…….our faculty…..

Person 1: We don't have time to meet the other batches….

Person 4: Most of the time we are in lecture halls

Person 1: the seniors are also at their lectures……. They do their clinicals in the morning and when they come for lectures…….. we're finishing our lectures and going for practical's

Person 3: Then the we don't meet each other

Moderator: Have you heard of any severe forms of ragging occurring here?

Person 2: I have heard of severe forms but not in the Jaffna university

Moderator: What have you heard?

Everyone giggling

Moderator: What have you heard? I'm asking you because I don't know and want to know the things you have heard.

Person 2: I have heard things in the news……. That in some universities……. They even take off clothes….. I've heard like that……

Moderator: Have you ever heard of things like that here?

Person 2:…….No…… some students………get stressed and go to the extent of getting depressed……..I've heard things like that…..not here

Moderator: Ive heard that some students want to give up their studies and leave, haven't you heard of incidents like that?

Person 3: Nursing……

Person 4: I think there are things like that in nursing…….

Moderator: What happens at Nursing (faculty)?

Person 4: Because the ragging is severe, 2 students from my area gave up studies here and went to Nursing school…….they came here to do nursing but because the ragging was too much they left….

Moderator: What sort of ragging was it? Were they abused physically or were they asked to take their clothes off?

Person 4: I think one of the boys were beaten……… not a lot but very frequently….. But I don't know much about the girl

Moderator: Do you think the boys are ragged more than the girls?

Several ……. More for the boys

Moderator: Do they get beaten?

Several together…….. not clear

Person 1: they just touch us but not physical with us (girls)

Person 2: I think they get beaten

Person 4: yes they get beaten…. Why we saw them getting beaten while going for (not clear)…….

Everyone giggling and agreeing

Moderator: Which faculty was this?

Everyone together……. The Science faculty

Person 3: Slap them, ask them to kneel and things like that…..

Person 5: things like that

Moderator: I have heard of very severe forms of ragging, are there thing like that?

Person 1: There are instances where students have died……. Died while they were being ragged…..

Moderator: Here (Jaffna University)?

Person 1: No…..no…..

Others giggling

Moderator: Where did that happen?

Several at once…….Peradeniya

Person 2: committing suicide

Several talking together….. (inaudible)

Person 4: Not too long ago……wasn't it?

Person 1: Inaudible

Moderator: What happened for them to die?

Person 1: they were physically abused…… hit or abused….like putting water and other things……

Moderator: So the Tamils don't rag the Sinhalese….right

Person 2: Only asks questions during the orientation

Person 1: They don't give us tasks or anything like that……..

Moderator: Is the ragging worse among the Tamil students?

Person 1: The Tamil students mostly come from their home (live at home)……so, ragging is less I think

Person 3: Yes…… ragging is less…….. they don't stay in the hostels and they only meet the seniors when they come to the faculty

Moderator: So you think that ragging is more among the Sinhalese?

Person 1: Yes……so…..

Person 3: Not ours……..

Person 1: Not that it is more……

Moderator: Not in the medical faculty but among the other's as a whole

Several people agreeing……. There is more among the Sinhalese
Person 2: Science faculty……has a lot

Person 1: They know you're in the hostels…… they don't come to your houses

Person 2: Agrees

Moderator: Does anything happen after the welcome party?

Person 4: No……nothing

Others agree

Moderator: In other faculties?

Person 4: No…… in other faculties the welcome party is only held after a year…….

Moderator: Why is it held after a year?

Person 3: They wait until the new batch arrives……

All the others agree

Person 3: they rag until the new batch comes

Several people say the above at the same time

Moderator: The ragging must be more severe in then because you won't be asking them to only sing for a whole year

Everyone gigges

Person 5: Nothing serious…… things like go and talk to a tree…

giggling

Person 1: They have the dress code for a year….. "Cheetha dresses" …….. there are thing like that…..

Moderator: So do these girls like to wear these "Cheetha dresses"?

Several people at once……probably not……giggling

Person 3: They have to plait their hair…….

Person 1: We saw wearing Kandyan saree……. Isn't it……

Person 4: Plaiting their hair…… wearing bathroom slippers…..

Several talking at once…….

Moderator: Cant they complain? Can't they say I don't want to wear these dresses?

Several at once……. No they can't say that

Giggling

Person 2: They will be called "aala" in the batch then…

Giggling

Several talking together

Person 3: Will be made an "aala" in the batch

Person 5: they will lose all connections with the batch

Person 6: They will not get any help from anyone or have connections with the seniors…..

Moderator: Are the "kuppi" (classes conducted by the seniors) compulsory?

Everyone together………..No……No

Person 3: Only if you want to attend

Moderator: Is that only in the medical faculty or in the other faculties also?

Person 1: No in all the faculties…..I think

Everyone together……all faculties

Person 2: Only if you want to go……..''Kuppi" are not compulsory

Moderator: Do you have to pay?

Everyone together……. No… No…..

Moderator: I have heard that you have to pay for these classes, and they are compulsory

Person 5: No, no you don't have to pay for these classes

Person 4: No you don't have to pay, they do it willingly……as a help to the students….

Few people agree and talk at the same time

Person 1: It's like something that has been continuing……..

Moderator: So it's a way of helping the juniors

Few people talking together

Person 3: It's like a continuing tradition

Moderator: I have heard that girls have to make food and go for boys…….

Giggling…….

Person 1: No……..

Several people giggling and talking ……inaudible

Person2: Not here, but it's there in other faculties……

Person 3: Tell to bring something…..

Moderator: What do you mean?

Person 3: Tell to bring something from home

Person 2: Tell us to bring something when we come back from home…

Several people together……..but there's no set rule that we must

Moderator: Have you heard that in the university, that students have been asked to bring things and they have problems because they can't afford to do it

Person3: I have heard that it happens in the "Rajarata" campus……

Moderator: Doesn't it happen here?

Several whispering……. Nooo………

Several whispering and giggling……..

Moderator: What did you say?

Giggling and muttering……..inaudible

Moderator: I heard that those thing happened here also

Person 1 & Person 2: Maybe in other faculties but not in ours…….

Person 2: Nobody asks us for food

Giggling & talking……..in audible

Moderator: Is there anything that you experienced that you didn't like?

Coughing………

Person 2: Didn't like………..

Whispering and giggling …..

Person 2: If a senior asks who they are and we don't know…….. they scold us a lot

Giggling

Moderator: Is that all?

Person 2: that's the thing we don't like…..

Moderator: Have they asked you to feed them or anything like that?

Person 3: No, No nothing like that……

Person 2: There had been before but not now……

Person 3: Not anymore

Person 2: Was there about 3-4……(inaudible)……….

Moderator: Do you think that ragging has decreased now?

Everyone agrees

Person 2: Almost disappeared now…..

Person 3: Yes…..

Person 1: It's getting less and less……much less than there was before

Moderator: You must be having older siblings…….when compared to them is it less?

Several talking together in a low voice…….in audible…….

Person 4: That’s here…..isn’t it…….

Person 5: Less here

Person 1: Very much less in our faculty

Person 3: In compression to before……almost nothing

Person 2: Just ask details about you……. that’s all

Several muttering at once…….

Person 2: Those day……. that is…….. Not Harmful……

Moderator: So do you think this sort of ragging……asking questions and things is good?

Person 1: Yes……

Person 2: Otherwise we won’t get a chance to get to know the seniors

Person 4: What we have is good….

Several agreeing

Person 5: What happens to us is good when you compare

Moderator: You have an introductory program during the orientation week; What’s good in that? What did you like in it?

Several people talking together……..it’s too much

Person 3: It’s too long……

Person 2: Takes up too much time…….

Person 4: We did it but it’s not very useful

Person 5: Wasn’t useful but prolonged out time…..If someone came with hope of improving their English, it wasn’t very helpful…….

Moderator: Does that mean that there were too many unnecessary things?

Several together……..No…… no

Person 2: Length was more than the substance…….

Several talking together……inaudible

Person 1: Have to pass this and the MBBS exam also…….

Person 4: Have to pass the English exam…….it feels like a waste of time because it's long

Moderator: What about the other lectures? Things like GBV, personal professional development (PPDS) …….Was there some lecture that was good?

Several…….. there were some

Person 1: Some were good……. inaudible…….

Person 2: They expect us to write everything in the exam……. that's the problem…….

Moderator: What do you mean?

Person 5: PPDS also has an exam……

Person 2: They expect us to write all the definitions and things in the same words they……so that's not good

Moderator: Does GBV also have an exam?

Several together…..Yes…..That comes under PPDS…..

Person 2: There is a big section called PPDS……. Then GBV is taught in that and these come in the exam…..

Several talking together……inaudible……

Person 2: There was a separate lecture on GBV also……. they taught about laws……… Someone from the law faculty came and did the lecture……

Moderator: Was it good?

Giggling

Person 2: the law lecture was too much……..we didn't understand anything

Giggling

Moderator: Is there something you think that should be added to the program? Do you think there is something that you will benefit from?

Person 2: It's better if it's done in a way that they don't expect an answer but in a more practical way….. better if it's done to give knowledge……

Moderator: Does the introductory program help to reduce ragging? Does it help you make better bonds with seniors?

Person 3: No not really……

Person 1: No in the orientation…….

Moderator: I mean in the introductory program

Person 2: The introductory program is a part of the orientation……..they make time for us to meet the senior batches……

Moderator: What do you mean?

Person 3: There is a hall like an examination hall……and they have like 3 hours to meet one batch that is senior to us…… this happens once a week to sort of get to know the seniors….

Others agreeing

Moderator: If you feel like it you go and talk to them?

Person 4: No they come and talk to us…..

Person 1: They prepare different programs

Person 2: No…. you cant ignore them…..

Person 1: they have different programs…..sports…..different things……

Moderator: Does ragging occur here?

Person 1: No it doesn't

Person 4: They ask us to sing, dance……do sports……

Person 2: Muslims……inaudible

Moderator: What happens to the Muslims?

Person 2: Nothing….. they can't participate in anything…..

Person 5: They never participate in anything

Person 4: Not in anything……parties, events nothing……

Moderator: Do they get isolated because of this?

Person 2: No they do it by choice……their seniors tell them not to…..and then they don't have any influence from the other races…..

Person 1: They remove themselves saying that the seniors told them not to……

Person 2: They don't come for sports meets……..not of anything…. Not for functions…….

Moderator: Their seniors tell them not to? What if there is a very social person who wants to participate?

Person 4: Don't know what will happen to them….

Person 2: They don't even have an item in the social….. girls cant but the boys have no problem……

Moderator: Don't the Sinhalese tell the Muslims to do things?

Several together…..No no

Moderator: Do the Muslims come and say anything?

Several together……..No……

Person 2: It's like they almost don't come to the orientation

Moderator: so they are only told not to participate?

Person 2: Maybe they do something separately…..

Person 1: They stand on side and watch…….

Moderator: So it's attendance compulsory for the Muslims?

Person 3: They just come and stand on a side and watch

Person 2: …….there is a person they was brought up like a Sinhalese person….here you have to go by the dress code…..you can only wear Black…….

Giggling

Person 2: She didn't like it initially……. Now she has adjusted…… But the foreigner junior girl won't listen to these things……

Moderator: You have a foreigner?

Person 2: A girl from Kuwait in our junior batch…….

Moderator: Does she become a target then?

Person 2: It's difficult to rag her……

Giggling

Person 3: The seniors get ragged by her……..giggling

Giggling

Moderator: Give me an example?

Person 4: You cant argue with her……

Person 5: Even if the seniors say something she doesn't take notice of what they say……

Person 2: She's friendly with all the ethnicities…….. The rest of the Muslim girls follow the dress code, she's not like that. She modifies it the way she wants…… The others have a problem of getting her to follow……

Person 1: Once they had told her but she ignored it…….She had said…. "don't come to tell me anything, I have paid and come here"…….

Moderator: Can you pay and come to this university?

Person 2: Only because she's a foreigner

Moderator: Then is she considered as an anti-ragger?

Several together……. yes……

Moderator: Do they restrict her from activities?

Person 2: She's friendly with students from other ethnicities….

Moderator: So she wasn't told not to participate?

Several together……sure she was told…

Person 2: I'm Sure she was told not to several times……but she didn't listen

Moderator: I have heard you cant attend anything if your anti-ragger but how come she's not told not to attend?

Person 3: No they don't attend

Moderator: So she doesn't attend these functions

Person 2: No others from the batch

Person 4: She comes…..

Person 5: She didn't have an item at the orientation……

Person 2: Did she come for it?

Person 1: Yes she was there…..

Others agree

Person 2: During the orientation they ask boys and girls to stand together like couples…… the other Muslim girls don't come but she came……

Giggling…..

Moderator: Are the seniors angry with her for not listening to them?

Person 2: Must be angry

Moderator: So you are also her seniors, aren't you?

Person 1: We like it when she's there….

Giggling

Person 2: It's more of a problem for the other Muslims because she's active and doesn't do as she's told

Moderator: So the Sinhalese and Tamils don't do anything it's only the Muslims?

Everyone together…… yes

Moderator: How are the anti-raggers among you treated?

Person 1: Anti raggers don't stay in the hostel……..

Everyone giggling

Person 4: it's not that they can't they don't want to stay…..

Person 2: They don't stay…… they stay in boarding's from the start

Moderator: they won't stay or mix all the years?

Person 3: They stay in boarding houses

Person 5: they come for sports meets…….and participate

Person 2: They don't come for "Kuppi", parties……they won't come to places where they will have to mix with the seniors

Moderator: Is it difficult to survive as an anti-ragger?

Several talking together…….in audible…..

Giggling

Person 2: They don't have to do seniors work……. enter their data, thesis work

Everyone giggles

Moderator: Can’t you refuse?

Person 2: we don’t refuse

Giggling……several speaking together…..inaudible

Moderator: what can you do to improve bonds and respect among students and become productive people?

Silence………

Moderator: Since you say there is less ragging among you all, what advice can you give the others?

Coughing

Person 5: In our faculty the lecturers are always present…… but in other faculties the lecturers have given up……. they stay aside and let ragging occur……the lecturers won’t get involved then the seniors behave the way they want and rag the juniors…..

Moderator: do the lecturers just stand aside?

Person 5: Yes they just do their duty and leave…….at other times lecturers don’t get involved when the seniors are ragging

Person 4: Just do the lecture and leave……. don’t get involved in anything else

Moderator: Any other ideas? What else can be done?

Person 1: Sometimes the campuses are closed and the academic period gets prolonged……

Moderator: What else can you do? Maybe something involving the parents……or……what else can you do as future leaders? If you have a child and they go to Arts faculty….. what can you do to reduce this?

Person 3: Punish…….. If you let everyone know that this person did something and was punishes…….

Moderator: Any other ideas?

Silence…………

Moderator: Is there anything else you would like to tell me about this, something that I didn’t ask you?

Silence…………

Moderator: Do you think it will help if you have any activities or clubs, like dancing, volley ball? What do you think? Will it help?

Person 1: It's good, but we don't have time with the lectures…….. there are students with abilities and who would like to do these but there's no time with the lectures and academics…. There is no time to do anything extra

Moderator: I have heard that sometimes the university doesn't give permission…. Is that true? Several people…. muttering …….. not sure

Person 3: If you have time you can do anything……..

Person 4: you can do anything…….. with disturbing your academics……

Moderator: Is there anything the Sinhalese get together and do?

Several people together…….. Vesak (Buddhist religious festival)

Moderator: anymore ideas to increase the bonds among students? Getting the parents involved?

Since…….

Then everyone speaking softly together……inaudible……disagreeing….

Person 4: the people is that when they get ragged in their first year they decide that they will do the same thing for their juniors…. That idea should be eliminated……

Person 2: shouldn't do they same thing to the others also……..
Moderator: if someone experienced very bad ragging why would you do it to another?

Silence…

Person 3: its less now….

Person 1: Less in our faculty……. not in others…..

Person 2: One entire batch shouldn't do what their told……then it will stop

Giggling

FGD 15

Moderator: Are all of you 3rd year students

All agree

Moderator: Where do you all live?

Everyone together….. in boarding houses

Moderator: Who many Sinhalese students in your batch?

Several talking very softly……..(inaudible)

Moderator: So, about 50% are Sinhalese

Person 1: Management doesn’t have 50% isn’t it?

Person 2: half…. Half

Person 3: About 50%

Person 1: B-com has about 60%.... Management has a little less then 50%..... maybe around 40%....

Several agreeing

Moderator: Introduction…… What is ragging?

Person 1: Ragging is a good thing from one side but on the other hand it could also be a bad thing…… From ragging the juniors….. because its done in juniors…. Most of the time ragging is done to increase the connection with the juniors… but unlike other campuses there isn’t ragging here……. But by ragging no one is mentally or physically….. if it’s done there is no harm caused to anyone..

Moderator: So, what is done in ragging?

Person 2: Ask them to write letters…. Sing songs….while that person is singing ask another person to clap…. (unclear) collected and done….

Person 3:…. Done to bring equality….. because there are students from different levels…..

Several agree…… whispering …..

Person 3: If someone is more neglected…… take that person more and ask her to sing and bring equality to everyone…. That’s how its used…. It’s the seniors responsibility to maintain it at a proper level….

Moderator: Do you all agree

Giggling……

Moderator: So, the good side is that you get to know people, so what is the bad side?

Several talking together….

Person 1: …… there's really no bad side

Whispering…..

Person 2: in other campuses of course……

Person 3: We don't know…..

Person 4: We have less Sinhalese here so… we always try to stay cooperative with them….

Moderator: So, the Tamil students don't rag you?

Several talking together…. No…no they don't

Person 3: Tamil students don't rag….

Moderator: they don't rag Sinhalese students. When you came to this university were you not scared that you will be ragged?

All of them giggling….. we were!

Moderator: Was that fear justifiable?

Person 1: giggling…… No….. giggling

Several talking together….. inaudible….

Person 2: That is the only thing that is left in the end (fond memories of ragging)……..

Several people agreeing and giggling

Person 2:…. We remember it and…..

Person 3:….. the rag is not a big thing….. what we say is…. To tell sing a song….. to give them a 'card' (to put a nickname)….. usually things that is in a campus….. in our campus, its much less… we don't do big things as ragging in our campus…. We don't have anything that put you down mentally…. We always find out who has financial problems and health problems, we ask everyone and find out….. we find these things out, after we give them things to do like sing a song……

Giggling……

Moderator: Alright, when you came to the university did you come frightened?

Several together…… yes….

Person 1: …..yes we were frightened…..

Moderator: After you came what were you asked to do?

Several talking at once….(unclear)…. Told to sing…

Person 1: ….dance…

Person 2: ….write letters….

Person 3: ……draw……

Person 1: Told to wear Cheetha dresses…. Wear our hair in 2 plaits….

Another agreeing…..

Person 2: Some Sinhalese, even when talking Sinhalese…… try to show a fake poshness….. of us initially in the first year first semester we had to talk only using Sinhala words….

Others echoing what she says….

Person 1:….. sometimes we felt scared… sometimes they scolded us….. when they saw our short comings they scolded us…. Those things were there but…

Person 2:….. we were happy…..

Moderator: So, everyone was happy?

Several talking and giggling…… yes……

Person 4: When we remember these thing we laugh…..

Giggling…..

Moderator: Still happy about it, that's good if you take the good side of ragging. Have you seen anywhere in the university where the rag was not good and students suffered?
Several together….. not here…

Person 1: ……not here…..

Person 2: here it isn't like that….

Several talking softly and agreeing……

Person 3: …. After we came…… sometimes due to other reasons students leave this and go….. but in the 4 years that we were here nobody left because of the ragging….

Person 2 agreeing and echoing what she said…

Moderator: So there haven't been times when a Sinhalese student fell into trouble or anything like that?

Person 1: Because of ragging?

Moderator: Yes!

Person 2: No….. nothing like that…

Several agreeing…..

Moderator: Even in the science faculty?

Person 3: …..science…..faculty……

Person 4: …..They……

Person 5: ….In the science …… they…… we don't know the problems they have….

Moderator: You know only about the management faculty? There is no problem and you are all very happy!

Person 1: …..Yes….

Moderator: Is there one group that gets ragged more? From better schools or lower schools or from Colombo schools?

Several people together…… depends on the person….

Person 1: It changes according to the person……

Several agreeing and repeating the same….

Person 2: …….Depends on the behavior…….
Person 3: It depends on how that person behaves…..

Several echo this….

Person 4: If they respect….

Several agree….

Person 1: If they don't respect…… they get scolded….

Giggling…..

Person 5: If they talk and are laugh…… then there's no problem…

Peron 6: ….. If they respect…… and have cooperation…… then there's no problem…. If they don't take notice of us or look the other way and walk ……. We help them so if they don't 'care' (ignore) about us…. Then they get highlighted….. things like that…..

Person 3: …..Depends on the person…..

Several agreeing….

Person 2: External things don't really matter…..

Moderator: When you say "depends on the person" do you mean from Colombo schools?

Several talking together….. no

Person 1: Colombo schools are less…..

Person 2: A lot less from Colombo schools… Colombo, Kalutara…..

Giggling

Moderator: So you do this to bring everyone together to an equal level and nobody has had any problem. Then don't you get ragged in your hostels?

Person 1: Hmmmm…..

Giggling ……

Person 2: When we were in hostels in the first year, our senior 'akkas' (older sisters)…… even today the biggest connection we have is with those 'akkas'…..

Person 3: …..is with the 'akkas' in the hostel….

Several giggling and agreeing…..
Person 1: After we went they came to the hostel after about 6 months…..

Person 4:…… they came to the hostel…. We had to wear Cheetha dresses alike…. They asked us to make tea for them….

Person 3:……(Laughingly)…. They told this also to the students who didn't know to make tea…..

Everyone giggling and agreeing……

Person5: they understand what sort of people we are……

Several talking….. they identify everything…..

Person 2: …..Even if we get sick……. We become alone here……. They are the ones that looked after us……. Now we have the biggest connection with the ones that ragged us……

Several agreeing…..

Person 1: We even have a group chat…..

Person 2: A group chat called the 'sisterhood', with them……

Moderator: So, with the people who ragged and didn't rag?

Several talking and giggling together…… (unclear)…..

Person 3: with the seniors and juniors…. giggling

Moderator: There are no students who didn't get ragged in the sisterhood?

Everyone together……. No

Person 4: ….Almost everyone got ragged…..

Person 5: ….There is a photo…….

Everyone agreeing and giggling…..

Person 5: It has all the Sinhalese females…… seniors and juniors…

Moderator: What happens or what does the sisterhood do?

Person 1: We just talk….. if there's some information or news, if you need to give a message….. giggling…..

Person 2: ….They have now gone on internships and are now doing jobs….. so we don't meet them….. we wish them on their birthdays…… wish someone if they are having their wedding……. So we have the connection…..

Moderator: Do they find jobs for you?

Several together……. Not for now…… we don't need it yet…….for research things….

Person 3:….. us about research and things…..and that helps us a lot…..

Person 4: Tell us about what's happening in their homes…..

Moderator: Where is ragging done the most? In the hostels, inside the university, in classes?

Several talking together…. Inside the university is almost not there….

Person 1: ….Not like in other campuses…. They are very alert…. Even….

Person 2:…. Even if there is a small….(unclear)….

Person 1: …… they come and scold us… even if we are talking about something good…. They don't even let us talk thinking that something will happen….

Moderator: So, the most happens in the hostel?

Several together…..even if there is a little bit occurring…… that occurs in the hostel…….

Person 1: …… because most of the time after evening lectures we are there…..so, it happens like that….

Moderator: So, you all ragged your juniors? (in a joking manner)

Everyone giggling and talking together…. We didn't get a chance…..we couldn't ……

Moderator: Why?

Person 1: only our batch couldn't rag the juniors…..

Person 2: There was a batch above us….. as seniors they intervened and we couldn't do anything….

Giggling…..

Person 3: ……They wanted to do all the batches….

Giggling….

Person 4: …..They managed everything and didn't give us……

Person 5: ……Even the batch below them…. we were not allowed to rag…..

Person 4: …..honestly we haven't ragged anyone….. giggling

Giggling…..

Person 6: ….. but if we meet them on the road….. we will ask them details about themselves… ask them to sing… or…..

Person 4: ……because of that…

Person 6: ….. we don't have a big connection….

Several talking together….. the connection between us is less….

Person 3: The connection we had with our seniors we still don't have with our juniors…..

Giggling….

Person 4:……but can develop….

Person 5: …. If they have some big problem…..they are not close enough to tell us about it….

Person 2: …..the relationship is not that developed yet….

Giggling…

Moderator: Ok…. So you think…..

Giggling……

Person 1: what they wanted was for all the junior batches to …….

Person 2: …..they wanted to…

Person 3:…… our seniors… they are a very powerful…..

Giggling….

Person 4:….. we were ragged….

Moderator: Alright, were you ragged by students from other faculties?

Several together…… no ….no…

Person 1: ……In the hostel….

Person 2:….. in the hostel there are students from all faculties……

Person 3: ….They wish us….. but don't take us separately and rag us…..

Several together…… they wish everyone….but ragging is only from your faculty….

Person 4: In the hostel….. seniors are seniors from which ever faculty they come and juniors are juniors which ever faculty they come from….

Moderator: They won't come and rag you in a bad way? Won't ask you to take your clothes off……

Everyone together…… no …..that's not done here…… there isn't any bad ragging here….

Person 1: Here there are only a few Sinhalese students here…. And we want to protect them…. so in this university we want to protect them….. we have that idea…… so because of that is not here…..

Person 2: Girls are only with girls….. if they are ragging….males only rag the males……most of the time…….

Moderator: So the males don't rag the females?

Person 2: no… they don't

Several talking together…..(unclear)

Person 2:……. Otherwise they even don't do that…..

Moderator: You haven't spoken at all…… what do you say?

Giggling……

Moderator: Have you got ragged?

Person 1: Yes…..

Moderator: but you didn't get scared?

Person 1: Was scared in the beginning but was alright afterwards…..

Person 2: ….. Have cried of fright also…..

Person 3: When we cry the senior say don't cry…. There's nothing to cry about…. Thry tell us not to get scared….
Moderator: Because of the rag, are there times you couldn't sleep or couldn't study?

Giggling…

Several together…… no nothing like that….. (unclear)

Person 1: when we remember these things we laugh now……

Another person agreeing…..

Person 2: When 'akkas' scold us and we come inside our rooms…… they listen at the door to see if we are scolding them….. we don't know this but later they said we did this…..giggling….

giggling……

Person 3: …..we became very united….

Person 4: We became very close….

Person 3: …..after they gave us the party….. they said that we don't study enough so when we try to sleep at midnight they make us send them a message….we also have to get up at about 5am and send them a message…

Several agreeing and echoing the same…

Giggling…

Person 5: We have to wake them also……

Everyone agrees…..

Person 2: we have to sing a song and wake them up……

Person 1: …..go to their room…..

Person 6: …..they also study and we also study…..

Moderator: So you all eat together?

Person 1: 'Akkas' eat separately….. seniors eat separately and we eat separately….

Moderator: You have an introductory lecture in the beginning. What do they do there?

Person 1: they say a lot about the university….. tell us the course manual, they show us what's in the campus, lecturers and professors talk….

Moderator: They do this in English?
Several together… yes

Moderator: Do you think some of them understand it then?

Several together….. no….

Person 1: ……there are some problems there….

Moderator: There are language problems? Only later that you start understanding English? How long does that take?

Person 1: ….that is at the start of the semester…. and towards…. The end of the first semester….

Several agreeing….

Person 2: it’s difficult during the orientation…. We have orientation for about 5 days….. its difficult then… we don’t understand then….. it won’t happen again…

Person 3: …… if we don’t know things…. it’s during our studies that we learn with difficulties….

Moderator: So, you don’t understand anything that’s said in the orientation?

Person 1: No, not everyone….. some don’t understand…..

Person 2: …..actually… some may not understand anything….

Person 3:….. (talking softly, unclear)…..

Moderator: Did you have a lecture on Gender based violence?

Person 1: …yes…. I think there was…..

Moderator: Do you have to go to lectures in the introductory period?

Several together…. No… not for lectures….

Moderator: Does everyone have to go to the orientation program?

Several ……yes

Moderator: How many days is the orientation program?

Several…. 5 days….

Moderator: Do you have to go for these 5 days?

Several speaking together…… no…. not really….. some didn’t go….

Person 1: you have to go but even if you don’t there isn’t a problem….

Agreeing…

Person 2: the late comers who get university admission late… they miss the orientation program….

Moderator: So, you think that the ragging should continue….

Several…. It should be there in a good way…..

Moderator:….is good, from that students get to know each other…. You learn things…. interact….. that's what you think, but what do you think about the bad side of it?

Person 1: We haven't seen the bad side of ragging in this campus…..

Person 2: ….. yes… we haven't seen……

Moderator: So, you have no experience of it?

Person 1: No…. not at all…..

Moderator: Nobody has told you even?

Person 1: In social media…..

Person 2:….. in other campuses…. We have heard….

Person 3: For some people with their family problems……

Person 4: …..have even committed suicide…

Person 3: …those things we have heard…. Even in the Peradeniya campus… sexual abuse and things we have heard about…. But to be honest we have not experienced these things here… we have been here 3.5 years but haven't heard of any incidents…

Person 2: (Agreeing and repeating the same)

Moderator: Do you think it's a good thing to sexually abuse students?

Several together….. no that's not good…

Person 1: ….. We are against that…..

Moderator: If your against it and hear that someone is doing something like that, what ewill you do?

Person 1: We will take action….

Several agreeing…..

Person 2: We will…..

Person 3: Stop it…..

Person 2:….. report it to someone and definitely take action against it

Moderator: You have come from other places, so what is your opinion of Jaffna university? Do they think it's a good place or a bad place?

Person 1: to tell you the truth….

Several together…… they get scared…..

Person 1:…. We are not afraid….. we know about this university…. But because of the war….. people have had bad experiences….. so as soon as we tell them…. they say "oh! Is it Jaffna?"….. this is actually one of the oldest universities.. my grandmother also studied here…. Those days Jaffna university was very reputable….. but with the war this changed…. Now when we say that….. they think we are going to a not a very good campus….

Others agree…..

Person 1:….. but really, ours is better…. The professors are very educated… so we are in better place than them….. more than other campuses…

Moderator: So, you think this is a better campus that Jayawardenapura campus where the first choice is? You think it's better here than there?

Several together…… no….

Person 1: Not that we are comparing…..

Person 2:… not comparing….. but….. in some ways they have a lot of chances….

Person 3: Professional …..(unclear)

Person 2: …..they have to cover their lectures….. they have compulsory attendance…. So due to this they maybe a few steps ahead of us….. but I feel that our lecturers are more committed than theirs to teach us….. they look at our books…. Those students laugh when we tell them these things…

Person 3: ….Some lecturers check to see if we have taken down notes….. if we are one minute late….. we are put outside….

Everyone agreeing…..

Person 4: ….. in terms of discipline…. It's very good…..

Everyone agrees…

Person 5: People looking from outside…… they always think…. are very scared….. more scared than us….. when they hear different news…..

Several agreeing and laughing…

Person 2: ….they even call us and ask….

Person 3: …..but there is a need for both sides (Tamils and Sinhalese) to come together…. We even go on trips separately……

Moderator: Sinhalese students separately? That's not good.

Person 3: ….. those things need to change further….. because….

Several agreeing…..

Moderator: Are there no love affairs between Sinhalese and Tamil students?

Several together…. Giggling….. there are….

Person 1: …. there was one….

Person 2: …. in the science faculty….

Person 3: ……there was a wedding recently from the Science faculty between a Sinhalese and Tamil student…..

Person 4: there have been students that have had affairs….. no there aren't any in our faculty….

Person 5:…. Before there were….

Moderator: Ok… that's something that happens outside, other than that don't your'll have any interactions with them (Tamils)?

Person 1:….. there is…

Several talking together…(unclear)…
Person 2: …..but only among a certain group….

Person 1:…. Everyone doesn't move (mix)….

Person2: …..this should develop…..

Moderator: Are you friendly with Tamil girls?

Everyone together…. yes, we are friendly…..

Moderator: But some people don't talk?

Several together…. No they talk if they see…. (unclear)……

Person 1: ……there are some boys also…..

Person 2: even in the lecture or……after we have a 'fit' (closeness)…

Person 3: …. There are some boys that don't like to move (mingle)…..

How do you find accommodation if you don't have hostels?

Several together….. we have to walk around and find them…..

Giggling…..

Several together….. also with the help of seniors……

Person 1: When we leave the boarding we hand it over to the juniors and go…..

Person 2: ….That happens depending on the connection between the two…..

Moderator: …..If not you have to find it…….

Several together….. yes….

Person 1: …Sometimes there are houses close by that are given out as boarding's….. sometimes when we walk around we find these… if not we take a boarding that was previously a seniors boarding….

Moderator: Do you take the whole house?

Person 1: yes… depends on the number of people….. it changes according to that….

Several agreeing and repeating…..

Person 2: … Any way the we are staying like that….. by taking 2 whole houses….. Several talking together….. not very clear…. One house has 10?….. the other house has 14?.......

Person 3; Our house has only 2 rooms……

Person 4: ….there aren't many facilities……

Moderator: So, you cook and eat?

Several together……. Yes…..

Moderator: Is the bursary you receive enough? Or do you eat from the hostel?

Everyone laughing…….

Person 1: …..Some students don't even receive the bursary….. giggling…..

Moderator: Is it the "mahapola"?

Person 1: The "mahapola" and bursary are both there…..

Person 2: …….some people receive both…..

Moderator: Does one student get both?

Several together……. No ……different people get one or the other……

Moderator: You must be receiving about Rs. 5000?

Person 1: Mahapola 5000 and the bursary is 4000….

Several agree….

Moderator: So, that's not enough?

Everyone agreeing…… yes…… its not enough…

Person 1: ….lots of people don't get it also……

Person 2; Some people only get around 300…..

Person 3: ….those days we used to buy water also….. now we have a filter so its ok now….

Person 4: ….. light bills…….

Several people repeating this…..

Person 3: …… its hot in the afternoon… so we put the fan…..

Person 5: …..the fans are broken…..

Several together….. no beds…. Just sleep on mattresses……

Person3: We have body washes in place covered by thin metal sheets…. Sometimes people come and watch us…..

Several together….. we have those problems….. giggling….. after 6pm we don't go out…..

Person 2: …..That problem is there more than other problems….

Several together…… a lot of perverts….. come to female hostels and watch the girls…. That's there a lot…. It's a big problem…. Even small children come and watch us….

Moderator: Even small children?

Person 1: Yes….. small boys who go to school also come and watch….

Person 2: Because they know where are strangers here and can't talk the language…. There are a lot of these problems….

Person 3: …Even if we go on the road……

Person 4: …..there is a difference…..

Person 3: …. Even if we go on the road… they know we are Sinhalese….. so, it's difficult to go on the road…

Moderator: So, you can't go alone on the road? And they come when you shower outside in the place covered with thin metal sheets…. But they don't worry you?

Person 1:….. so far there has been no problem…..

Person 2: They touch us and do stuff if they get a chance…….

Everyone together…… they are not afraid…. When we walk on the road…. They touch our bodies….

Person 1: …..last week……

Person 2: …touched me…..

Person 1: ….. touched her and went….

Several talk together….(unclear)….
Person 3: …..touched the head…..

Person 4: has happened to all of us……

Person 5: Happened during daytime….

Several together……. During the day…… when people were around….. they are not afraid……

Person 2: ……more than inside the campus…. Things happen outside…..

Person 4: ……there are no problems inside…..

Person 3: ….We are frightened when we go out…… we take poles and go out…

Person 1: …stones…. Giggling…

Person 3: .. we are very cautious….. one near the door and another outside…..

Person 2: …..safety is not enough… we are always scared at night……

Person 3: ……We keep stones and things with us….. we are very scared after 6pm…..

Moderator: Have you not told this to the University?

Several together…… they cant take any action regarding this…..

Person 1: if the UGC gives us hostels for all 4 years…..

Several agreeing……

Person 2: Our juniors are really suffering…… we had our hostels close by…. They have their hostels in Induvil, kondavil……

Person 3: they have been kept in several places….. when we came we were all in one hostel….

Person 2: ….Those girls showed us that there were some insects in their mattresses and got biten….

Person 4: they were like burn marks…. A big rash…

Person 2: ….they really suffer….. they take water from here sometimes…

Person 5: ….they have to take small buses….and they get harassed in those also….

Person 2: …they press on them…..and a lot of these things happen…..

Person 1: ….we had a meeting with them recently…. And they cried….

Moderator: Don’t you have a welfare officer?

Person 1: After complaining to that person they have come and sprayed the mattresses…. But it hasn’t worked….. that insect comes out for the light…. A very small insect… they say the insect is still there…..

Person 2: If they had hostels close to the university….

Person 3: …..they have taken a house and put 15 students separately in a house…. Girls…..

Person 4: We stayed in hostels and knew a bit before we went to boarding's in the 2nd year….. when they put like that at once they get scared….because of the men….

Moderator: What about their food?

Person 1: ….they get from the science….. sometimes cook and eat…..

Person 2: But its still far and 15 of them are alone…

Person 3: If a lot of them stay together as soon as they come…. Then there's nothing to be afraid of…. The whole batch is together….. but when you put a few like this they have safety issues….

Person 4: they face a lot of problems due to this…. language problems .... when they go to a shop…..

Several talking together and agreeing…..

Moderator: That must be a problem. All your student counselors and everybody is Tamil isn't it?

Everyone agreeing…..

Moderator: Aren't there Sinhalese teachers?

Person 1: ……..in the library….

Person 2:….. even if they are Tamil there are some that can manage Sinhalese…. Not everyone….

Person 3: Some can't understand….

Person 2: ….there are one or Two….

Person 4: We don't even have a Demo….. that is Sinhalese…
Person 1: There is no one Sinhalese…..

Moderator: So, you can improve your English, because you have to speak to them in English.

All agreeing…..

Moderator: If you have any problem…. Even while going on the road, who do you complain to?

Person 1: …..there's nobody to tell….

Everyone talking together and giggling…

Person 2: We tell the boys to come soon….

Person 3: …..(inaudible, talking we softly)… the boys in the batch came and helped us…

Person 4: we cant tell our families these things….

Everyone agreeing….

Person 4: even if we get sick…. Due to the distance we hide it from our families…

Person 5: ….If we hear any noise or get frightened…. We always call the boys….

Person 4:…..the boys or the senior girls….

Person 5: …..the boys wont' think of their safety and come and help us….

Everyone agreeing…..

Person 4:….this is a problem for them also…

Everyone agreeing….

Person 3: ……now there is Police from about 6pm….

Person 5: but even if they are there…..

Several talking together…. (unclear)

Moderator: …you don't have problems form the Police officers?

Everyone together….. no….no…

Giggling….

Several together….. they are good… they are Sinhalese….

Person 2: They also say …..try to stay safe….

Person 4: even when I went out…. There was a man on the wall watching me….. that was around 8.30pm…… when we ask someone to come…

Person 2:….. by then he has gone….. the person that comes knows the roads well to escape…. Then he hides soon…..

Person 5: …they stole a phone…. They came inside the room….

Several talking together…. (unclear)…

Person 4: Safety wise…. Its awful…. There is a better boarding…. Someone came there and came inside the room also….

Moderator: So, if it is so unsafe, haven't they tried to steal your money when your at lectures?

Person 1: ..that hasn't happened yet…..

Giggling…. But it can happen…

Moderator: So all they want to do is look and you and frighten you?

Everyone agreeing…..

Person 1: Some don't like Sinhalese staying here…. They came to the boarding and caused a problem in the beginning….

Person 2: because of the war….. they are now scared when Sinhalese come, that the priority they have will reduce….

Moderator: So that's what they Are worried about.

Person 1: but that's not everybody….

Person 2:……Speaking softly…. (inaudible)….

Moderator: So, why don't you come here when you pass out!

Several together…….. they wont give us….

Moderator: Why Demos?

Several talking together……. They wont take like that….. science has a few….. a majority of the demos in science are Sinhalese….

Moderator: So, what more do you to say? They like ragging so they don't have recommendations. They just don't like the harassment, they have no other recommendations.

AW: Do you have any recommendations for a university that has ragging? Like Peradeniya for example?

Person 1: They should rag to a limit…..

Person 2: They should know their limitations….. if they are doing more than that….. in our university…. If they do something with bad intentions….

Person 3: ……we are also against it…. If someone scolds with bad language or anything… the senior most batch…. Should call out that person in public and ask them to apologize in Public…..

Person 2 agreeing….

Person 3:…. Should check if they are doing something wrong…. But if it can't be stopped… then inform a more bigger person….

Moderator: Have you done something like that?

Several …..no nothing has happened…. But have apologized…..

Person 1: Have made phone calls and used bad language….

Several talking together…..

Person 2: ….have got caught like that…. we inquire about things like that…. but as soon as we find out

Person 3: We hear things….

Person 2: ……if we find out….. we bring that person in public and ask to apologize…. For that we take steps as seniors……

Moderator: So, you take action to prevent others from doing bad things.

Several together…. Yes… yes….

Person 1: …..even if one person does something….. all the rest stand against it….

Person 2: Even if it was a senior who did something to a junior ……that person ahs to get forgiveness from the junior…..

Person 3: …….have a meeting and get everyone together and ask them to tell what they said…..

Person 2: …….and accept their fault…..

Person 3: ….if they accept the fault….. and what they will do in the future….. then scold the person and do everything to be done and from there and shake hands and give the person cooperation…. Otherwise that person will have a problem….

Person 4: in this campus we try to keep everybody together……

Several talking together…… to keep the Sinhalese together…..

Moderator: Due to that you don't have sexual, physical things like hitting or have any other bad thing? So, you try to bring all the batches together like that, and give forgiveness. So, you all think it is a good thing. Then they get insulted, isn't it?

Person 1: then everybody gets to know about that person…. Then that person can't do anything further…..

Person 2:….. even a person who does that gets scared….. that they will have to come in front of everyone and get forgiveness…

Person 3: Then they wont do anything to their juniors also…. They know it's a bad thing…..

Moderator: Thank you very much!

FGD 16

Moderator: Introduction. What does ragging mean to you?

Silence…..

Moderator: Say something (smiling) how can you became Managers someday?

Person 1: We come from different areas of the country…. We don't know where each other come from…. In the beginning what happens from it is we get used to the environment….. we get to know each other…. It teaches us how to adapt to the university environment basically…. But not……

Person 2: ……It changes according to the limits….

Person 3: …….it builds comradery…….

Moderator: So, that's how it starts, this subculture?

Person 1: There are different levels in society….. everyone who comes here sits for one exam called the A/L's…. when we look at from an educational point of view, everyone has the same qualification…..but their family background is very different……. One will be from Royal collage (famous school in Colombo)…. But another could be from an unheard-of school in a very remote village……. Can be a school that doesn't even have classrooms….so that student is used to a different life style….. the other student has a different life style….. maybe the student from Royal Collage associated minister's and parliamentarian's children and people from a higher level in society…. When these students meet the other students most of the time…. There ideas and opinions are different…. Where different ideas and opinions…..

Person 2: ….. the gap…..

Person 1: …… are brought together… they reduce the disparity in the different levels of society and bring everyone to the same level…..

Moderator: ….. you said there are certain limits, what are those?

Person 1: …… we see and hear different things in the media….. ragging is most often seen as ….. as shown by the media as a bad thing….. What he said is what the main aim should be……

Moderator: …. alright, that is the main aim but does everyone act accordingly?

Person 1: …..usually in our university….

Person 2: …..Doctor, it's like this…. in our university of course… track(?)(unclear) like the media says is not there… there are a lot of opinions….. if you take Peradeniya for example….. about 3 years ago… there is an incident that everyone knows about….. in the Peradeniya agriculture faculty…. That is not ragging in my opinion…. Its something to done to hurt….. or due to the mental problems of the raggers….

Person 3: ……or a mental disorder….

Moderator: So, the mental disorder is in the students ragging?

Person 2: …..Yes, students who rag…..

Moderator: What you're saying is that the aim of ragging is to bring all the students to the same level?

Person 1: …..Isn't that a valuable thing, miss?

Moderator: …. Yes, that's a good thing. But while trying to do this different people do different things? Is that it?

Person 1: …..Yes, that's it…. If it exceeds the limit, its wrong…..

Person 2: …..Apart from that doctor…. I want to add a small thing….. In Ragging, the things that are assumed doesn't always occur…. It happens less often….. a lot of these things are a dramatized… why do I say it's a dramatization (over representation)….. we were also in the first year, so we should have also felt that pressure… We felt nothing like that but we saw……. That some students…when they come to places like this…. are extremely shy and don't talk at all…. That means that they have not even spoken at school….

Person 3: ……it could have even been one of us that is talking now…..

Person 2: ……even we haven't spoken that much in school….these things are done to reduce their shyness…. If any thing is done, that's why….. or if there is a reason or meaning to it,

this should be it… not to harass them and get fed up of this….. I have to specially mention another thing…. in this campus, there is nothing called ragging…..

Person 4: …….not even previously……

Person 2: ……what I mean when I say nothing happens is….. we don't have to specifically rag students to develop unity in this campus…. Why… because this campus has no water…. Its is the real situation…. The water that is here has a high mineral content and is difficult to even drink….. even the food is different from the food we are used to eating…. Then unity automatically develops…

Moderator: Because of the problems….

Several together…… to solve problems….

Person 1: …..we talk and try to solve everything…..

Person 2: …we don't have to create problems….. there are problems when we arrive….

Moderator: But when we talk about this…. sometimes, they beat students and things, why didn't you wear a certain outfit or they can say something. When you were juniors….your from Chilaw, Negambo, Ja-ela and schools from there, where you not frightened when you were coming to the University of Jaffna?

Person 1: We were scared because we were coming to an unfamiliar area….. but apart from that….

Moderator: I'm not saying now.

Person 1: …… it was not there after we came here…..

Moderator: So, when you entered the university, you had a fear.

Person 1: ……a fear when you say doctor…… this is a different area…. This is what we were scared about…..we have language problems…. Even now our English is not very fluent… when we came our English was also very weak…..

Person 2: ……even more from the Tamil side…..

Person 1: …..language….. because of the language problem we were a little afraid…..

Moderator: When you entered university, weren't you worried that there would be ragging?

Person 1: ……. Hmmm…. that fear…..

Person 2: …..we didn't have a big understanding about that…… we didn't know much about ragging…..

Person 1: ……. what I mean….

Person 2: ……. After we came here our seniors looked after us better than we expected……

Person 1:…. That's what happened….

Person 2: …..there are only a few Sinhalese…. These few… more than ragging it has become their duty to look after us…..

Person 1: ……from the day we came…..

Person 2: …..they have checked up from what we eat even….. they brought us the water…
Person 1: ……because we didn't know these things….

Person 2: …. what they did for us, we are doing for our juniors….

Moderator: Alright, I am looking at this very independently, what I'm saying is when I went to university, I was scared. That's because I didn't know what sort of ragging I would get, that's what I mean. Didn't you have any ideas like that? Weren't you scared about what will happen? Haven't you seen and heard things in the media and television and felt scared?

Person 1: …..we have heard that there is no ragging in Jaffna university….

Person 2: ……yes… we were of that opinion….. there were people we knew from before…. Seniors from our school….. because they were also here, we were not so scared….

Person 3: …..the general atmosphere which is seen in some of the main universities is seen less here…..

Person 1:…… knew it was less….

Person 3: ……there is only a small number of Sinhalese here…..

Moderator: Do you get ragged only by the Sinhalese students?

Person 1: …… when you say ragging… it means…..

Person 2: ……some one who explains things are…..

Person 1: …..there isn't much ragging……. The maximum….. they tell us to sing a song…. That is really not a rag…… we can sing if we want….. if we don't want to we can refuse…

Person 3: ……the other thing, miss…. Is that it is an opportunity for us….

Person 1: ….if we do it doctor….. we can move forward….. apart from that ……nobody forces, scolds or beats us…..

Moderator: Are you forced to wear a particular outfit?

Several together…. No…. they don't….

Moderator: why the juniors, now they are wearing sarees?

Several together…… there is a culture here… (unclear)

Person 1: ……they give a week for 'freshers'….. to wear a particular outfit….. it is actually….

Moderator: But we saw students wearing 'cheetha' dresses, are those things alright?

Person 1: Most of the time that is for identification, doctor…

Moderator: But for identification even…. I'm not blaming or saying it's wrong. This information will not leave these 4 walls….. this is only for her research. We have to speak the truth, who likes to walk in a single file? Do you like that?

Person 1: Doctor, it's like this…… this is a culture here….

Moderator: No, I'm not talking about a culture. Where is there a culture that your whole family will walk in a single line?

Person 1: It's like this….. we are still not used to this environment….

Moderator: So because of that, you're afraid you will fall and walk one behind the other?

One person laughing…

Person 1: ….Miss, it's like this…

Person 2: …. No doctor…..

Person 3: … previously we spoke that different people are from different levels and should bring everyone to the same level…. This could be one way of doing that…. when one person is going one way another doesn't have to walk in line…

Moderator: That means you are trying to organize them.

Several together…… yes to organize them…….

Person 1: Apart from that…..

Moderator: Like in the army….

Person 1: …..if your saying, Miss…. That are forced to wear those clothes…. We have already got our welcome from our seniors but we still wear the same clothes?

Moderator: You can't throw away the trousers you bought? (laughing)

Several laughing…..

Person 1: That's what I'm saying, Miss…… it's easy to identify students in the university …..

Moderator: Why do you have to wear those clothes?

Person 1: Miss, when we first wore it we though it was easy….. now we have understood that its easy….. sometimes you wear what you have…..

Person 2: Doctor….. sometimes we have t-shirts…

Moderator: it's alright, no matter how you try to justify it, the way I think can be different. Isn't it?

Everyone agrees.. yes, yes

Moderator: Now say, you're starting a new job…. Then I tell you to come in shorts and white short sleeved t-shirt and cut your hair short and come. Does that not put you down, mentally?

Person 1: Aghhhh……. But…..

Person 2: …..It's like this miss…. We are not at a job…. If we are pressurized….we will be….

Moderator: No, it's like this, if everyone else there dresses nicely and the people who come there dress nicely and the staff also dress nicely. Even though you justify it, what I'm saying is that we destroy the students self-respect.

Person 1: Doctor…..it's like this….. it's not for one or 2 people….. it's for the whole batch….

Moderator: You destroy it for the whole batch. Do you accept this?

Person 1: I have a small thing to say….. if you go to the Police, during the Police training period they have to wear shorts…. We also do a training…..

Moderator: University is a place to speak open, you can express your ideas, there's freedom to do so. But in the middle of this freedom, you can't treat students like prisoners. That's all I'm saying. Ok, let's not talk about this. How long do you do these things and how long do they have to wear these clothes? Whether they wear it or not, and they do it properly and you don't harass them.

Person 1: 6 months.

Several agree…

Moderator: Do they sexually harass and take off the shirts and trousers in the hostel?

Person 1: Nothing like that…

Several agreeing….

Person 2: ……. There's nothing……

Person 3: …..I say this responsibly…. that there is nothing…..

Person 4…… (unclear)……. So there's no problem like that….
Person 1: ……there are other students from other faculties……

Person 2: …..students from different faculties are mixed…..

Moderator: Don't students from other faculties harass you?

Several together…… no, no….. that never happens…

Moderator: So, management faculty juniors are ragged by management faculty seniors?

………Silence……..

Person 1: to call it ragging……

Moderator: …ok, not ragging, but taking extremely good care of you and hold your hand and taking you around…..(laughing)

Everyone laughing…..

Person 1: …..(unclear)…..

Moderator: I should write letters to your parents and ask them why they didn't look after you all because it's only now that they are being shown the correct way (laughing)

Some laughing ….some quite…..

Moderator: These students that go in line, how do they end up at the student riots at 'Lipton circus'?

Silence…..

Person 1: We have not been involved in those……

Person 2: nothing like that….

Moderator: (laughing)….. alright let's let that one go as well….

Person 1: We are not involved in those things…..

Moderator: Although you are not involved in that, all students in the entire university system wear Black pants and white shirts and walk in line, so, that means that some student somewhere from these 16 universities have broken the line and gone for these strikes, isn't it? (laughing)

Several laughing….. not commenting….

Moderator: Alright, now let's stop lying and say that is the culture. Do you think apart from what you say, beating students, harassing them, removing their clothes is that good?

Person 1: It’s wrong!

Person 2: ….. Those are absolutely wrong……

Person 3: …. That’s puts them under mental duress…..

Moderator: What will you do if you find out that someone is being put under mental duress?

Person 1: ….. if something like that happens….. we have a batch….. we can act as a batch…..

Person 2: …. Most often things like that don’t happen…..

Person 3: ….if something like that happens….. the unity…..

Person 4: …….if something like that happens….. then that person…….

Person 1: ….If that happens …. Doctor…. The unity we build during the rag can be used here also…..

Person 3: ….. even the university has actions against it…..

Person 2: ….. if something like that happens….. in a place like this if something happens like that the student won’t stay here….. because it’s more dangerous….. we don’t condone that behavior… it’s not condoned in any campus…..

Moderator: If by chance something like that happens, can you all get together as a batch……

Person 1: ….that doctor…. The unity is defiantly used that is created by the rag….

Person 2: ……we automatically go into action…..

Moderator: Have you all done anything like, say if someone does something wrong, ask that person to ask for forgiveness in front of everyone?

Person 1: ……something like that…… we haven’t seen….

Person 2: ……We didn’t understand…..

Moderator: Say someone did bad ragging and then after identifying that person, bring him forward and ask him to get forgiveness from…..

Person 1: ….. you mean tell a senior to ask for forgiveness?

Person 2: ….there have been no incidents to ask for forgiveness….

Person 1:…… incidents like that don’t occur…

Person 3: …..they are prevented from happening…..

Person 1: talking about ragging…… in this campus… there is hardly anything called ragging….. I say this responsibly…… if there is something happening….. it happens in an organized manner…… what we mean by ragging is….. gathering everyone and…….

Person 2: …with everyone informed….

Person 1: introduce one another…… sometimes from that the unity we build…. When we newly arrive…. We don't know who the other person is…… they don't know who I am….. they don't know about my parents…. We don't know about the socioeconomic status…. To get to know these is the reason that we are grouped together….. there put 4-5 students together and ask them to do a little drama…. That is what I see as a rag…. When this is done according to a plan…. There is no reason to ask for forgiveness because there isn't any bad ragging….. there is no necessity….

Moderator: This was told to us by someone in your university. You don't have any 'brotherhood', don't you have anything that the juniors and seniors get together and do?

Person 1: There is….

Person 2: …..We have a lot of things like that….

Person 3: ….. we have Vesak (Buddhist religious festival)….. we have cricket….

Person 2: …… we have sports tournaments…..

Person 4: …When we start from the Sinhalese new year program…… during the whole year…. We have something every month…..

Moderator: So, the Sinhalese student's activities are different from the Tamil students?

Person 1: ……yes we…..

Person 2: ….. Somethings……

Person 1: …..everyone gets together and has a meet…… the Tamil and Sinhalese students get together and have a meet…..

Person 3: ….festival called "Marcos"…..

Person 2: ……a sports meet…… and cultural show…. Everything is there…..

Person 4: ….apart from that….. faculty meets are there….. apart from that also….. we have an "Avurudu" festival (Sinhalese new year)…..

Person 1: ….. we have a "Poson" festival (Buddhist festival)….. for "Vesak"…..

Person 3: ……..even last year….. the Tamil students…. (unclear)…..

Moderator: Do you all have Tamil friends?

Several together….. yes……

Person 1: ….Yes everyone talks to each other…..

Moderator: Do you still stay in the hostel or outside the hostel?

Person 1: ……. We have boarding's also….

Person 2: …..in the hostel…..

Person 3: …..yes…..we still have hostels….

Moderator: Are some students targeted more for ragging? Say for example, if they are from Royal collage and don't mix too much, do they get ragged more than student's who listen to instructions?

Person 1: No…… there isn't any ragging like that….. because they are sent in a line from the start to build their mentality… that's why they ask to go in line and things…..

Moderator: What if they refuse to go in line?

Person 1: that doesn't happen, doctor……. When you fix them from these small things….

Person 2:…… they themselves will feel that they will be cornered…. When a lot of students are doing the same thing….. when one person doesn't do that thing….. the other will not like him and then they will get cornered….

Person 3: …. They will get scared and automatically adjust…..

Moderator: We learn about the Gaussian curve where 95 will be in the middle but the will be 5 outliers. But even that doesn't happen and 100% are inside?

Person 1: There isn't anyone like that…..

Person 2: ….. we have to make sure that there are no outliers ourselves…..

Moderator: So, you don't have any anti-raggers? Not a single anti-ragger in the management faculty?
Person 1: It's like this doctor…. There is no necessity to become an anti-ragger in this university…… then there should be a severe rag….. Now in Peradeniya….. both my elder brother and sister are at Peradeniya….. My sister is an anti-ragger because the Peradeniya campus has ragging….. this is well known… My sister is in the Agriculture faculty….. the faculty that had a problem….. She is 2 years senior and she is an anti-ragger…… even if my sister hears the word rag….. she gets angry….. that can happen in a university like that…… there is no environment for something like that to happen……

Moderator: Let's leave out the University and let's talk about the faculty. There is nothing like that in your faculty?

Several together….. no

Moderator: Have you seen or heard of Tamil students ragging Tamil students?

Several together…….no…… not really….. don't know…..

Person 1:….they are more kinder than we expected….. even their seniors treat us with a lot of affection….. they talk nicely to us….. there is a thing like that….

Person 2: ….we haven't seen their rags..

Moderator: No, no that's not what I meant. Tamil student rag Tamil students and Muslims rag Muslim students, is that how it happens?

Whispering among themselves…..

Person 1: ….. haven't even seen anything….

Person 2: …. hmmmm…. Haven't seen anything even….

Person 3: …to be honest …we haven't seen anything…..

Talking between themselves…… Muslim's talk to the Muslims….. (inaudible)…..

Person 1: It's like this doctor, we only meet them at the lecture halls…..

Moderator: Your lectures are compulsory. You are very friendly with the Lecturers also, am I right? Do they look at your books and things also?

Person 1: More than in other universities……. because we have friends in other universities….. they are more keen in our university….. the actual thing is that attendance at our lectures….. in other campuses they sign for their friends….. that is difficult to do in this campus…..

Moderator: How many Sinhalese students do you have in your batch?

Person 1: We have 170.

Moderator: The whole batch?

Person 1: 107 girls…… and boys…..

Person 2: ….. the whole batch has 350 students……

Moderator: From that half are Sinhalese?

Several ……yes…..

Moderator: So, more girls than boys? Do the boys have other problems, like they are not allowed to do sports?

Several together…….. no …..nothing like that….

Person 1: …..they always tell us to do sports…….

Moderator: During the first year is there a rule that the juniors can't go to the canteen?

Silence…..

Whispering…..

Person 1: there isn't is there?

Moderator: For new students? Was it there for you?

Person 1: We didn't have it….

Person 2: ….. we bought food from the start…..

Person 3: …. When we first came….. there was an 'aiya' (older brother)…….

Person 4: …..we didn't have the time to eat from the canteen……

Person 3: ……there is a place…that we can get food that we are used to eating……

Moderator: Where is that?

Several together…. Near the campus…

Person 1: …. He brings the food to the hostel and gives it to us……

Person 2: … that's easier for us….

Moderator: So, the army doesn't give you food now?

Person 1: They gave us food when we first came……

Several people talking together and saying the same thing….

Person 2: …..more than that…..this is easier for us……

Person 3: …When you compare it with the time the food arrives……

Person 4: ……we can tell them how we want our food sent….. we can tell them, if we say send us food with chicken….. then he sends us food with chicken….

Moderator: Food is Rs. 60?

Person 1: 60, 80….. there is food for different prices…..

Moderator: How many food packets does he make?

Person 1: ….any amount…..

Everyone agrees……

Person 1: …..he makes them and sends it to the hostel……

Person 2: …then we have no cost…..

Moderator: Then you don't need to eat from the canteen. Where do you eat from in the morning?

Several together….. from the same person….

Moderator: what does he give for breakfast?

Several people together…… rice…

Person 1: ….. rice….. sambol…..

Person 2: …you can ask for things like 'Kottu' (Sri Lankan street food) too…..

Moderator: In the night?

Person 1: Rice for all three meals….

Moderator: So, all three meals are rice, but you prefer rice. You have an introductory program, what do you do in that, what lectures are there?

Person 1: It's an introduction to the university……

Person 2: ….. it's an introduction….

Moderator: So, that's done in English isn't it? Did you understand it?

Person 1: there was a small problem there….. it was actually a problem…..

Person 2: …. you can't say it was 100% successful….. maybe 50%

Person 3: ….it's not that we didn't completely understand….. we understood but to a certain degree…

Person 4: it's like this doctor….. what happened there was that they kept on introducing lecturers to us and nobody can practically remember like that… but apart from the language the thing we received the most was….. we learnt about the places in the university… the library…. IT hall…. The lab.. where are lecture halls are…. Where the ground is…. Things like that… we learnt the basic things….. but apart from that we learnt a little about the culture without language being a problem…. But the other things we can't say were 100% successful…

Moderator: Now you are males, don’t you get alcohol and things here?

Person 1: No…… not inside…..

Others mumbling……

Moderator: To the hostel?

Person 1: There is no permission to bring to the hostel….

Several echoing this statement….

Person 2: ….. there is no permission…and……

Moderator: For birthday parties?

Several together…… we cut cake…. Bring cake….

Person 1: … we cut cake….. that’s what we do…..

Person 2:….. we do that…. we get permission for those things…..

Person 3: ….. we can do those things but without too much noise…. Without obstructing the others….

Person 4: ….we do things like that…. cut cake…..
Moderator: Alright, you guys are such pure Buddhist children, but if someone wants to buy alcohol, where do you buy it from? Are there places?

Person 1: We don’t know of places like that…..

Person 2: ….. we don’t know….

Moderator: (laughing) Don’t lie.

Person 1: Doctor, I will tell you something….. recently we went on our batch trip… went to Hantane…. It was a 3 day trip….. even in that… alcohol was not consumed…..

Person 2: …..alcohol was not consumed…..

Moderator: So, Sinhalese students try not to drink, is that how it is?

Person 1: …… that is….. in our batch.. they didn’t take….. there wasn’t a large necessity…..

Person 2: …..laughing…. the students….. (laughing)…they don’t have…. They are still learning…. (laughing)…..

Several laughing….

Person 3: ….. if you want check and see….(laughing)….. in the batch trip…. Nothing like that happened….

Person 4: …..in their private lives….. when they go home…. When they go for a party…..

Moderator: I am not asking about you all. I always tell you that I'm not asking about you. I'm asking if even the other students don't bring alcohol?

Several together…. We don't know…

Moderator: These students are not like that. They go like they are going to temple, don't see what happens around, know nothing…. Such loveable students. (laughing)

Several laughing…..

Person 1: …. Person to person …… the ones that need to must be doing so when they go home and things….. but other than that……

Moderator: In the hostel aren't there parties and…..

Several people talking at once….

Person 1: ….. if that happens….

Person 2: …..you are not allowed to have parties in the hostel…

Person 3: ….. you can't do that…..

Person 4:…. Its forbidden….

Several echo this statement….

Moderator: In the boarding?

Person 1: Although we have paid for the boarding…… we don't know much…..

Person 2: ….we are still in the hostel miss……

Several talking together…. (unclear)…

Person 3: …… we have another 2 months……

Moderator: But you haven't even heard if they drink in boarding's?

Person 1: We don't know….

Person 2: ….they must be drinking….. we don't know….

Moderator: I lecture young people. There are 2 main things about them. the first is that they like to try new things, they like to try alcohol, try smoking, drugs. The problem we have is

trying to get them to stop. If you don't try these things it's a problem with your youth. Then you aren't youth, but infants.

Several talking together……

Person 1: ……it's like this…..

Person 2:….. we like to stay in the hostel the whole year also…..

Person 3: ..its cheaper to stay in the hostel also….

Person 4: It's like this miss…. If there is a problem in the beginning we will not get hostels in our 4th year….

Several talking together…. (unclear)….

Person 2: …..We're not saying that everyone doesn't use alcohol…. Maybe because of these reason people are afraid to use alcohol in the hostels…

Several talking together….. not clear…..

Moderator: Now you are students of the management faculty, and we invite you to talk at, say the Peradeniya university. Because this is a very good place with absolutely no ragging, what advice would you give to stop ragging in that place?

Person 1: It's like this doctor…. As I said ragging…..

Moderator: No, I'm talking about severe forms of ragging.

Person 1: If it's extreme form we can talk against it anywhere…..

Person 2: ….for the incidents that occurred recently…. Police had taken action

Person 3: ….the UGC……

Person 2: …..you can suspend them….. can give them out of bounds……

Moderator: So, the anti-ragging action can be implemented, if they exceed the limits? Is that what you mean when you say stop? We are asking you because you are young people. That is the extreme of the punishment. Say if we caught a thief or murderer…. That is the final outcome…

Phone ringing….

Moderator: without coming to that point, is there anything that can be done to change it before? To change their ideas?

Person 1: It's difficult…… isn't it… there is a limit to how much you can fix someone's mind….. different people have different ideas….. so it's difficult…..

Phone ringing again…..

Person 2: …..if they are doing things like that….. anyway we have to go to a legal side….

Person 3: …..if it's done like that….. it's unfair to that person….. harming are person or their rights is a harmful thing…. so action should be taken against it….. to direct them to take action….. the first years or the new comers when they first come and have their orientation…. Should inform them that if something happens…. because ragging is supposed to build unity but if it's harming them….. they should take legal action….. then if there is something like that….. it gets eliminated……

Silence

Person 2: Miss, is asking……

Person 4: ….. if we can do some program…..

Person 2: ……without going to maximum…..legal side if there's anything that can be done…..
Person 3: ……that's what can be done….. there's no point putting it in the head of the seniors….. the juniors should be told……

Moderator: No child, before you came to the university, even in the A/L classes some children harass others, isn't there a way to stop it in schools?

Person 1: If you tell the teachers…..

Person 2: ..Some people don't like to change their extreme ideas…… then they won't listen to…..

Person 3: ….then you have to have religious programs or……

Person 4: …. but I don't see…. We are also males…. So I can't say it will work 100%....it will work for a while…. But it will happen again…..

Person 1: After a while that thing comes back to your head…..

Person 4: …While your associating your friends and hanging out….. it will reappear…. You can suppress it for a while…… we can have religious programs, meditation and things like that but it's only short lasting……

Moderator: So, you can't change that person. You enter university after a lot of hard work, and don't they feel bad to harm somebody then? Or do they act as mentally unstable individuals? What do you think? We need to stop this. Say one of your friends went to Peradeniya and was harassed a lot, then what can you do? Isn't it wrong that they torment him? Everyone should have the same rights, isn't it? How do we correct the mentally unbalanced students?

Person 1: it's like this miss….you said that during their schooling days….. there are people to harass a lot…. Say one of them came to campus….. then what is their goal?….. to bring

people from different levels down to the same level….. did you understand, miss? ……so, if this (ragging) occurs according to a plan….. then again during the rag…. The junior knows what to do when he becomes a senior…… if he doesn't do that… then the others will scold him and fix him….. then I don't think there will be a problem…..

Moderator: Thank you very!

FGD 17

Moderator: In your university there was a boy that was ragged and committed suicide isn't it?

Several together….. inaudible

Person 1: ……..there was a boy who committed suicide……

Moderator: He was from the engineering faculty here wasn't he?

Person 1: He was from the engineering faculty….. but you can't say he was from here….

Person 2: ……. He was from Moratuwa……

Person 1:…… he got transferred from here…..

Person 3: …..you can get transferred from here….. he went on a mutual transfer from here to Moratuwa….. it happened after he went to Moratuwa…… that he committed suicide….

Moderator: But in his suicide note….

Pia: Speak louder or this will not get recorded

Moderator: This is not for that, this is just a normal chat

Person 1: he went from the engineering faculty…

Moderator: He got transferred from here because he was getting harassed by you all. The he says that the was harassed there too. That is what he said.

Person 1: to be honest madam, we from the engineering faculty don't know anything….

Moderator: Ok then, what can you say about the technology faculty?

Person 1: Our faculty madam, when you look at it with the other faculties….. in out faulty in our batch out of the 220 students, 132 students are Sinhalese… the majority of students are Sinhalese…… in our faculty….. not like in any other faculty in Jaffna…. I don't think there are so many Sinhalese….

Person 2: ….. most are Sinhalese…..

Person 1: according to our experience in Jaffna… after coming to Jaffna something we noticed was… Ragging among the Tamil students…. Among the university level students…. We don't have to lie, every university has ragging isn't it…. The Jaffna university… the Tamils use ragging in a different way…. What I mean madam is they have a caste system…. Because of their caste differences…. Most of the time… the Sinhalese students in the university…. We don't have to lie…. When compared with our experiences (of ragging) …. most of the time the difference is like the moon and the stars….
Person 2: ….. more than the Sinhalese students there is a big difference among the Tamil students…..

Moderator: I'm talking about the majority….. these things are not of importance…. I just want to know if ragging is carried out, where it's done, how it's done, who does it…. Small details like that. It happens, you cant say it doesn't…..

Person 1: ….Yes it does….

Person 2: ..it means madam, what we strive to do is….. if you really see…. After arriving newly in the campus….. I was… I didn't know this one is from Badulla, form Nuwara Eliya, Marawila… I didn't know anything…. From the 4 roommates I got…. One was a Muslim, the very next day he left the room…. after that 2 students came…. Even the 3rd person didn't come…. One was from Nugegoda, the other was from Marawila… I was only with those 2 students the first 2 days…. There were 112 Sinhalese students in the batch, but I only stayed with those 2 students… Madam, I didn't even talk with rest of the 110 students…later the Aiyas (older brothers) …. The senior students told us to come to the canteen one day… they came and told us…. "you have come to the Jaffna University, there are a lot of Sinhalese students here, this is the faculty, it started…" they gave us some history about the university…our Aiyas are the first batch in this faculty…. They gave us some history like that… they said "the only thing you have to do is memorize our names"…. All of the Sinhalese, Tamil, Muslim…..

Person 2: ……. Otherwise we don't know anybody….. we don't know who is there…. We are from different areas… this person is from Badulla, Nuwara Eliya, Galle, we don't know any of this….we don't know anything and we don't have any connections…. When we get together… we can get to know that this person is like this… and talk…

Person 1: (agreeing)

Person 3: …..Even if there is a funeral….

Person 2: …(unclear)….. we should know about this…. when we come to this campus those are the friendships that remain with us…. To have a friendship we need to form a connection… because of that (ragging) that a connection formed…. Otherwise we wouldn't even know the names of others….

Moderator: Alright….

Person 1: ….So….

Moderator: But you do things like "packing"?

Person 1: What is that?

Moderator: You remove everyone's clothes and put them under the bed, right?

Person 1: …. No…

Moderator: You don't do that here?
Several together….. No ……no…. things like that…. in universities…. Jaffna university….

Moderator: I'm talking about what they say, that occurs in universities generally

Person 1: Madam, I think your talking about what you said occurred recently….. about what went around in the media….

Person 2: …..in the Ruhuna campus….

Person 1: in the Ruhuna campus… what happened along with the Marxist situation

Moderator: No, no, I know about these things before that incident…..

Person 1:….. in our….

Several people talking together (inaudible)…..

Person 2: Maybe they did it before but……

Person 3: ……we don't know….

Moderator: Ok, ok, then what do you say about the Marxist involvement?

Person 1: …..That madam…..

Several talking together…… (unclear)

Person 2: …..If I was to talk about it….. because it's connected to where I live…. I think that along with time…..

Several talking together…. (unclear)..

Person 2: ….with political….

Several people talking….

Person 2: ……maybe it's actually some political thing……

Moderator: But if they said that then it is a bad thing, do you admit that?

Person 1: yes, its wrong if they said that….

Several talking together…. (unclear)

Moderator: So, you were absolutely not subjected to anything called ragging… what I mean was nobody in your batch was harmed…

Person 1: ….No….

Moderator: I live in Sri Lanka, I have heard lots of things. Wasn't there an incident that the boys shaved their heads and the barber sent bill? Was it in Vauniya or Kilinochi?

Several mumbling

Moderator: Where the barber shaved the heads of students but they left without paying the bill and the barbers protested…

Several together….. we don't know…. It was not us….

Person 1: It wasn't our faculty….

Several laughing…

Moderator: You don't know about that incident?

Person 1: We don't know…

Person 2: we only cut our hair when we go home…..

Person 3: …. Maybe students living here…

Person 4: ….we are growing our hair…

Moderator: So, you had no problems, you have a lovely time! You had no problems!

Person 1: We had no problems….

Person 2: …..was there a problem?

Moderator: I don't know? I won't take any action against you. I wont take your names or anything. This is all I want, lets speak the truth! The government spends a lot of money to ensure the mental wellbeing and educate students. I'm just telling you the concept. From the money we spend we cant count the number of students that become unhealthy…. I can see that the 4 of you are strong. There could be another 4 students that cant tolerate the ragging and suffer mentally. But, they are also students who came to the university to study. If they suffer mentally and cant continue their education, is there any point. How many students are there like that, who quit the university! What I'm saying is, why do you…. I have taught in the university for 40 years. I know very well that you are not babies. I know that ragging occurs. We should try to find ways to stop this happening. So all we want to know is why this occurs, where it occurs and things like that.

Person 1: Miss, you are very much above us. We are still at the bottom. We are considered to be like dirt (dust), and you are 4 levels above us. If you came down 4 levels to the level of students, you will understand what the problem is.

Moderator: That is what I'm saying. Tell us the problem. I don't know what's wrong…..

Person 1: …..really miss, if we take the ragging problem in the faculty, you say that students are quitting, and other things…. to tell you, from our faculty only 2 students have quit…. One joined the Army… the other became a Buddhist monk….

Person 2: …..it's not for the reasons you said…..

Several talking together…..

Person 1: ….. so, miss…. When you talk about our faculty…..

Moderator: Faculty…..

Person 1: …we can only take our faculty as an example….. and our friends….

Several talking together….

Moderator: but as students of this university you mix around and know a lot of things….

Person 1: …yes…

Person 2: ….so…..

Moderator: Tell of this thing about being "dust"

Several talking together….

Person 1: Dust is what you always trample…

Moderator: …No it's not... when you wear slippers, you don't walk on dust…

Several talking together…..

Person 1: ….yes but the slipper also trample that "dust"…

Several saying the same thing…..

Person 2: …..someone tramples us….

Moderator: We are not trying to trample you, we are trying to lift you up by giving you an university education….

Person 1: ….when your trying to educate us…. Why are the people above us trying to put us down by talking about ragging….ragging….ragging?

Person 2: We are saying that there is no ragging…..

Person 3: ……..you want to stop ragging….we also don't want ragging….we want while we at the university…. Our fellow students (brothers)… to bring them together and go on a journey together…. We also want to somehow study well….

Several students talking together in an aggressive manner….

Moderator: Can I ask you something? The students that come from villages, do they stay in different groups? Do the students that come from Matara stay together, other Nugegoda stay together? Is it like that?

Person 1: it was like that…..

Person 2: ……It's there (ragging) to stop these thing….

Several talking together….. to stop that and build togetherness….

Person 3: Now you said Madam, …..(unclear)…. People who come from our area…. Usually these students will enter university…. We know… even our A/L teachers will say that this student is going to campus…. Then I will connect with that person… so, we're coming to the same university…. So the students who come….. the students from Matara group together, from Colombo group, from Kandy group, Badulla group… it's different sets of people that come as a group of friends….. after coming to the hostel…. After you meet your roommates…you still try to hang out with the few friends you came with…. When we are asked to memorize peoples names…. To be honest… I had never even seen him (another member of the FGD)…. Until I came to the campus and stayed in the hostel… that was when I first hear their names….. that is what…..

Moderator: that's not what I'm asking. You didn't answer my question. What I'm asking is do the ones that come from villages eat separately or do you eat and do stuff together.

Several together….. yes….

Moderator: There are somethings that whether we like it or not we have to accept, so in this university, are there some places that are known to be places where ragging occurs? Like hostel rooms….. are there places like that?

Person 1: No….it doesn't happen…

Person 2: it can't happen in hostel rooms……

Several people talking together ….(unclear)…

Moderator: What I'm asking you is….

Several together….. no, there are no special places….

Moderator: So, do you rag each other in the same batch or does your immediate seniors rag you?

Person 1: ….there is nothing called ragging, madam…. There is no physical violence…..

Person 2: …..that physical violence…..

Person 1: …..there is no physical ragging… we have not experienced it…..

Several agreeing…..

Person 1: …..we madam, are talking about ourselves….

Moderator: As 2nd years, have you seen this happening to others in the campus?

Person 1: We don't know about the campus…

Person 2: ….we only know about us….

Person 3: …..We have not witnessed anything physical happening in any of the 3 faculties here……

Person 4: …I don't know if we were lucky but nothing happened to us……

Person 1:….. they didn't even touch our arms….

Moderator: Oh, that's very good. So, you also didn't do anything like that?

Several together….. no….

Moderator: Everyone says it's a social conflict, that you are trying to equalize everyone and bring them to the same level. Is that right?

Person 1: Yes, that's true…

Several agreeing…..

Moderator: So, why does it bother you that different people are in different social classes?

Person 1: Social classes…..

Several talking together….. (unclear)

Moderator: Are you not studying to elevate yourself?

Person 1: Your right…..

Several talking together….. (unclear)…..

Person 1: …..ok madam, I will say it like this…. If I go to the canteen and I have only 60 rupees, he has 100, and the other one has 150 and 200 rupees…. According to the prices in our canteen…. I can eat a vegetable rice, he can eat a fried rice and drink a Coke Cola with

it…..ok… Sometimes, he will eat that and then eat an ice cream also afterwards…. Then I can see him eating a fried rice…. I am with him…. I can see someone eating a fried rice, I can see him drink a coke, I can him eat an ice cream….. we are in the same batch…. we sit for the same exam….. we study in the same campus….. we came to study together…. Then why is there a difference between 3-4 people?

Moderator: Why my child, that is the way of the world!

Person 1: That is the problem!....

Person 2: ….that is true….

Several talking together….(unclear)….

Moderator: Some people may drive a Mercedes Benz, but I drive an old broken car….even the junior lectures drive newer cars, but I don't feel bad about that.

Person 1: That's alright…..but… thinks let's think like this…. we are also trying to study hard and come up to a certain level…. But to come up to that level we should all be at the same level in the start….

Moderator: … You can't force a people to do things….

Several talking together….. (unclear)….

Person 1: ……even we like…. We like…..There are things that we also like…. He's eating an ice cream… to drink tea…. Even I like to eat ice cream…. I would like to eat a little ice cream… wouldn't you like that….

Moderator: No you can't like other peoples stuff…

Everyone talking together in a very aggressive manner…

Moderator: (talking loudly to be heard over the loud arguments)…. Let me ask you this…. there is a beautiful girl, what if you like her and he also likes her….

Person 1: ….no that cant happen….

Everyone arguing loudly….

Person 2: ….you can't do that…..

Overserve intervening to keep the peace….

Person 1: … not desires like that….. desires towards food….

Person 3: ….food and drinks…..

Person 1: ….. those are sort of sexual desires….they are not relevant here….

Moderator: but those are all desires?

Everyone talking together…..

Person 1: …..we are not talking bout girls…..

Moderator: I am telling you these things and young people…. What your trying to do, by saying your trying to equalize everybody can never happen in this world. Am I right! By saying that you are going to do that, and causing a social issue by ragging students is not going to achieve that. What I'm saying is that, it won't happen.

Students talking while moderator is talking……

Person 1: What your saying is right…. We can fully understand what your saying. What your saying is to let people be in their social strata….

Moderator: that's not what I'm saying….. what I'm saying is that you should all be together.

Person 1: yes , yes… that's true…

Moderator: Just because you harass a person they won't come together. They should have learnt in school that everyone is equal and should be treated equally. You can't force a person to do that…..

Several together….. we don't do that…..

Moderator: you try to do things by force by ragging them….

Several people together….(unclear)

Person 1:…… we just try to make them aware….

Moderator: That's exactly what I'm saying! You all trying to make them aware is what leads to you ragging them….

Person 1: ...No madam….

Several talking together….

Person 2:….what we mean by making them aware is…. When we become seniors…. The admin always sees us as….. the 2nd years, 3rd years and the 4th years are seen as….. even if they talk to the juniors they think that we are ragging them….. even if a "malli" (younger brother) comes, I can't talk to him or take a photograph… they think its ragging…. The situation is such…. So, your saying that we are trying to equalize the society… it's you! Not me!..... you said that they should come down a bit…

Moderator: the ones on the top should come down a bit….

Person 1: then miss, what do we have to do? …..we have to talk to them, be friendly with them…. but what if they don't want to be friends with the ones in a lower class….

Moderator: So, what?

Person 1: ….listen to me!....

Several arguing together…..
Person 2: the problem we have is not… saying so, what!.... hmmm…say, there is a famers son…. They are in a lower class, isn't it….

Moderator: …You cant say that. Why the president we have now is like that!

Everyone arguing again…..

Person 1: ….these days a farmer is at the level of drinking poison…..

Moderator: It's not like that… the president….

Person 1: …... I don't like to talk politics inside the campus…. If we leave politics aside… and talk about the students… and discuss how we can stay united and in the same level…. Then if the other person is not coming to our level…. What is the problem they have…. Why cant they come to our level?... why cant they talk to us?... then, our seniors will come and make that person aware, and tell him to go and talk to that person… If he doesn't like it…. Then we give them a (I don't understand the Sinhalese word "Muhuvimak". Maybe it means a chance or a warning) ------- and tell them to learn names and places the students come from…. We share our knowledge…. Understood?

Moderator: Ok, I'm trying to learn from you….if they won't come to that level… if they don't want to come to your level, then you do a little ragging…. Ok, let's not use the word ragging but you bully them, harass them a little?

Person 1: For example the boy that stayed in his room, the boy from Nugegoda….. even now he stays in his level… we haven't bullied, or ragged or forced him….

Several talking together….

Person 2: …even now he walks alone with his Coke Cola…

Several talking together…. (unclear)

Person 3: ……he drinks his Coke alone without sharing…..

Person 4:….. he doesn't share….. but….

Person 1: …we cant do anything about it….

Moderator: So, why didn't you fix him?

Person 1: ……that's the thing madam…..

Person 2:….. we cant….

Person 3: ….after making aware….

Person 4:…. Wont listen…..

Person 1: …..he was made aware…. But he refused to accept it…. And because he refused to accept it, he stay at his level…. The people who agreed to it got together and joined him and they make their own decisions….

Person 2:….. he's in our faculty…..

Person 3: ……you may think we treat him like "alla" (potato – the students that are isolated because they are against ragging)…. This is what you say we do….

Moderator: I never said that. I don't know, what "alla" means. I only know about "alla" curry.

Person 1: ….madam…. it's like this…..

Moderator: First of all, tell me what "alla" is?

Several talking together……

Person 2: there are a lot of universities… so you should know what "alla" means…

Moderator: I'm from the medical faculty. We don't use the word "alla" there. We teach and do everything in English there.

Several together……

Person 1: but you knew what "Packing" was…

Moderator: Yes.

Person 1: I'm sure when you go and speak in different places, you have heard this.

Moderator: but….

Person 1: …..we sometimes say, he was isolated and excluded from the batch…. That's it….

Moderator: oh, ok.

Person 1: We don't do that to that boy…. We talk to him well.

Person 2: If he wants to borrow one of our bikes, we lend it to him.

Several talking together….

Moderator: Let's talk about intentions. There are different students in society. There are some students from higher social levels and then there are others. They have problems and then you

said you do this, and then that. Then you said Farmers are the poorest and when their children come you try to be fair to them…..

Person 1: ….. there are poorer people than the farmers…..

Moderator: ….yes, I know….. I'm talking about the poor students, then when they come here you try to treat them fairly. Because of that, the students at a higher level and the students at a lower level, you try to bring together and try to make them share…..

Several talking together….. not trying to make them share….

Moderator: …ok, then what….

Person 1: ….. not to share…. I eat a lunch packet for 60 rupees with my roommate…..understood!

Several talking together…..

Person 1: ….. but can he do that?....

Several people talking together…..

Person 2: ……Can he afford that?.... he may not have money to eat lunch that day….

Moderator: Then?

Several talking together….(unclear)

Moderator: Then you want to bring society to the same level because, you may not have food to eat. Ok, what are the other reasons?

Person 1: Now, different people have different levels of awareness….. now look, there are people from different schools…. Now, in Sri Lanka there are a lot of schools…..

Person 2: …….there are poor students also….

Person 1: ……even in those schools….. now even in "Ananda", "Nalanda" (famous schools in Colombo) there are students….. even in our batch…. Now, even their level of awareness could be different…. You can imagine with technology and things, the knowledge is different….

Moderator: …..but even students like you attend those schools…..

Person 1: …..yes….that's true… say they went there with a lot of hard work…. But like that students from every school come to the university… they have come here isn't it… then they all in one society…. Then some people are aware in that society…. Let me tell you an example…. Some students got new phone only after coming to university… until then they didn't have a mobile…. They used a normal phone… then sometimes they don't know the functions of the phone…. There are some that have been using mobiles and have a good knowledge about it… so, there are students like that… when we come here newly… take

those students….. they can be proud and arrogant…. Then students like that wont help and teach the students who don't know…. How are we supposed to exit the university?..... weren't we supposed to learn something?..... But sometimes we don't know things like that…. then in the society…. If we join everyone…. Then we can share knowledge and then learn about technology…. Then everyone learns about something….
Moderator: …..(unclear because students are talking over her)

Several agreeing together…..

Person 1: ……now….. you spoke about forcing students… miss, we haven't forced that person….

Person 2: ….. then he would have come to the canteen then….

Moderator: Ok, when the junior students come do you get a chance to get to know them? This student is acting high and mighty, this student is…..

Several students talking together……(unclear)…..

Person 1:…… then we tell them "malli, talk to your people and fix them"…..because it's not us….

Moderator: …. Ok, so, then you tell your juniors to fix the students of their batch by telling them and doing….

Person 1:…… by telling and doing meaning… we won't go and tell them if you don't fix yourselves we are going to hit you and do this…

Moderator:….. no, I don't mean to say that…..not hitting….

Person 1: ….we come here after leaving school, isn't it… we see our parents at home, the few friends we associate, and people from our village… when we come here it's a completely different society….. we see lecturers, people from the surrounding area, our own brothers and sisters, people older than us….. there are several different groups of people we meet…. Then sometimes there are students who can't handle living without their parents……

Moderator: …..that's exactly what I'm saying! I told you that there are some students that can't endure things!

Person 1: …then imagine miss…. If we think I don't care, it's not my problem, let anybody be the way they want to…. And we will mind our own business… you said miss, let people stay in the social level…. No, problem…. Then when one person has a problem, why are we like this…. when another has a problem….

Person 2:…. We are humans…..

Person 3: …..just think…..

Person 1: ….. if everybody is separate in the faculties….. then miss, for that…. we go and…. To he honest…. We show them the way…..we were showed the way… or…. Even if our

mother and father were there, they would say “son why didn’t you fix yourselve” they would ask this….. my parents have said, it doesn’t matter if they are rich or poor, to go and talk to them…..

Moderator: Are you catholic?

Person 1: No, I’m Buddhist….

Moderator: you said you were from Matale, isn’t it?

Person 1: So miss, when we see every society…. There are students that haven’t seen different societies…. Because when they went to school they were only with their parents, and after coming here they couldn’t tolerate it….. and couldn’t bear the grief of separation…. And couldn’t move or socialize with the others…..

Moderator: …so, you also work in way to help these students also…..

Person 1: ….yes to help them…..

Person 2: ….we help them…..

Person 3:….. if they don’t have money, money or…… we give them…. sometimes clothes…. If they want to go somewhere and don’t have a new t-shirt….. the student who became a monk… I have bought clothes for him….. we help them….

Person 4: ….we support them…..

Person 1: …we come to a level where we share everything….. in that way (thru ragging) we at least learn a name….

Person 2:….. now even….. we can’t help them without knowing these friends that much… what’s the harm in talking a little bit….. we should at least have a small connection to come to that point….

Moderator: So, at no point do you harass or tease or….. have you not seen anything like that in this entire university?

Person 1: No madam!

Person 2: …….we don’t have the chance to see what’s going on in the entire university….

Person 3: …. We have heard some things….

Several talking together……

Person 4: …we have heard there was ragging and there have been inquiries…..

Person 1: ……we can’t even meet the juniors….. we can’t even talk to them like brothers….

Moderator: Oh! So you can’t even talk to them…..

Several talking together……

Person 1:….. we can't even talk…..

Person 2: ….. we ……we …..

Moderator: …. Oh! So, you can't even get to know the girls….

Person 1: ……if we talk there are complains….

Moderator: oh, so, then you have a big problem. But you know that if we talk about your university and other universities ragging, like you said has become a topic of discussion, that says students are harassed, this many students have dropped out of university, so, what are your thoughts on this?

Person 1: Yes! Definitely students have dropped out!

Moderator: Why is that?

Person 1: They drop out because…. Do you think miss…. Hmmmm…. Like that…. hmmm… students who stay with their mother and father, will come and stay in places like this…. in places like Kilinochi?

Person 2: Do you live alone madam?

Moderator: No child, I'm married.

Person 1: You live in Sweden?

Moderator: No, I'm from Sri Lanka. I'm from the university of Sri Jayawardanapura.

Person 1: ….Oh …..that's why you know so much….

Several talking together…..

Moderator: …. that's why I know what you up to when you even think about it…..

Several talking together……

Person 1:….. when compared with the Jayawardanapura campus……

Person 2:…… this campus……

Several talking together….. (unclear)……

Person 3:…… ragging…..

Person 4:…… there's no need to rag…..

Person 1: …..to be honest……

Person 2: …..there's nothing else…..

Moderator: Can I give you some advice, I'm not like your mother but grandmother. You, as university students should maintain the respect and behave well, you can get good jobs in the private sector. Have you seen the advertisements in the private sector?

Person 1: ….. that's the thing….

Moderator: ….it says "State university candidates are not entertained". Have you seen those ads? This is in your hands. It's completely up to you. You have to be smart, you should speak English, you came here to rise above, that's why your in university. You should prevent students dropping out. Do you receive help from your teachers?

Person 1: …. It's like this madam… really speaking, no.

Person 2: …..we are talking straight…..

Person 1: madam….. remember you spoke about humiliating students…. I was humiliated by the person called the acting Dean…. I was humiliates by this person….. to tell you the truth, we don't have money…..

Moderator: To give a tea party (a welcome party for the new students)?

Person 1: Yes…. To give a tea party…. We…. A few weeks ago we went on our batch trip…. That also, we only collected 1800 rupees per person for 2 days and to eat and drink and for everything….. that was last month…. And it was only last month that we went to the boarding houses…. Then we have to pay 2000 rupees for the boarding also….. it's not like Jayawardanapura (SJP) the prices are like the moon and the stars……

Moderator: How much is a lunch parcel in your canteen?

Several talking together ……(unclear)

Person 1: ……vegetable on is 60 rupees, if it's meat or fish, it's 80 rupees. Mixed rice is 100 rupees….. it's expensive here….

Person 2: Madam, can you understand how expensive things are here?

Several people talking together…..

Person 3:….. as I was saying…. They humiliated me…. The senior student counselor, when he came said that they will give us money to have a the batch party, the juniors welcome….. because we couldn't collect a lot of money and because he said so, I went and asked them for money….. then they started laughing at me…. They spoke to each other in Tamil and laughed at me….. they were saying "Kassi", "Kassi"….. I understood that they were laughing at me because I asked for money…. They laughed at me for about 3 minutes…. To be honest madam, I got angry….. I got angry… I got fed up… I have travel so far from the other corner to this corner… my mother is also a teacher…. My mother has taught me…. Really my

mother didn't bring me up to be humiliated by anyone… I'm even talking to you now with that confidance…. But at that time I honestly got very angry….. I thought at that time, I don't even want this degree…. I'll just leave and go home…. I was so angry… then I spoke to another teacher and that teacher said that they will give the money….. otherwise I would have said something… because I couldn't control myself…. To tell you madam, there are times that I can't control my temper….. with sports… and things in school and sports and things my temper has reduced…. But there is a limit….. the that limit is exceeded, I lose control.

Moderator: That is very good you said this. Imagine there is a new student and he also can't control his temper. But he can't even express this. He can't hit anybody. That is what we say. I know you are very good boys that don't do these sort of things but if you know it's happening somewhere you should be able to stop it. You fall into a miserable situation if you can't take any action in a situation like that. What you said is a very good example. When a senior person makes fun of you, you mentally fall, isn't that what you said? I said before that the 4 of you are strong boys but if this happened top another person they would think "oh, I was ridiculed". Isn't it? So, when you came here….. are you the first batch or the second batch?

Several together……2nd batch

Moderator: Weren't you worried that you will get ragged? Usually they say these things to students who are going to an university know about this.

Several together…..

Person 1: We thought there will be ragging when we came…..

Person 2: ….even friends said that there will be…..

Person 3: ….but although we thought we will be ragged…. We weren't…..

Moderator: So, you didn't have any problems? So, the student union protects you? You are in the union aren't you? You said you were.

Person 1: Yes…. Yes… I'm a representive….

Moderator: so, you take students and go on strikes to the "Lipton circus"(place in Colombo where the student unions strike)?

Person 1: …..to tell you the truth……

Several laughing…..

Several talking together….. (unclear)…..

Person 2: ……madam, you are from SJP…..so you know the true situation of those conflicts…. Then madam….. it's a different university….. there are students from SJP university…. I have friends…. When I speak to them, I have to explain everything to them about Jaffna, about the Jaffna university… Jaffna university madam, is completely like a university in South India…. Why is that…. because everyone in the main student union is

Tamil….a majority…. In that way the Sinhalese…. You must be knowing madam…. In our main university….

Moderator: Yes, I know about the student union. I have heard about it.

Person 1: …. If any faculty is doing a function…. The Art faculty has to be involved primarily…. On those conditions, we haven't even walked around Lipton circus….

Person 2:……we have never….. really, we haven't gone on a Picketing (stike)…

Several talking together……

Moderator: What is the support does the student union give you?

Several talking together….

Person 1:…. We get a good support…. Recently… Madam, if you remember we were on a holiday during the month of February…. During that holiday….

Moderator: ….oh, did you know that we came at that time?

Person 1: Yes…..

Several people talking together…..

Person 2: ….we were informed about you…..

Person 3:….. senior students informed us…..

Person 1: …..that incident also occurred….. because one of the warden's hit our females.....

Several talking together…..

Person 1:….. then they went to the Police and did a lot of things….. then in the end they put the fault on us and we had to go to courts…

Several talking together…..(unclear)

Person 4:…….. still that female is suffering….

Several talking together….. that female…..

Person 1: ….still there is no justice…..

Person 2: …..still it's not resolved…..

Person 3: …… things like that happen to us…..

Person 4:….. for a mistake done by the….. we….

Person 1: ……it's always like…..

Person 2:…. Miss, you are on ……the side of…. You have to see every side….. you are not on the side of the students?
Moderator: I am on the side of the students.

Person 1: If you think of it from that side…. Then why, during that situation was nobody on the side of that female student?

Several talking together……

Moderator: it depends on how you presented the situation.

Person 1: We gave evidence…. There were police reports….. we had all the evidence…. Even after doing everything… we were treated unfairly….. we are the ones that had to go to courts…. We are the ones that had to spend money… we are the ones still going to courts also….. still the females student is……

Person 2:… to tell you the best part….

Person 1: ……we presented the evidence also……

Person 2:….. if we are talking about the incident…. Both the parents were brought to Jaffna and what they had asked in the inquiry was…. They have said…"you are the one that ragged, you are the one that ragged"…..and showed photos….

Several talking together……

Moderator: that's the thing you…..

Person 1: …..then the main student union gave us a huge support…..

Person 2:….. to tell you the truth, we unlike all the other campuses in Sri Lanka, in education we are…… we are still in the 2nd year 1st semester…. But our counter parts in SJP have don their 2nd year 1st exam already….so basically they have done their exams and are on vacation before they start their 2nd semester…. Then you can see, how behind we are…

Person 3:……with conditions like that….

Several talking together…. (unclear)……

Person 4: ……so when you are in the same faculty for so many years…..

Person 1:…..so to study even….. to do the work we want to do….. if there are lectures at that time due to these barriers….. we won't get the chance… sometimes they are rescheduled but there are times that we can't even do that…

Moderator: Do you have staff? They must be young and closer to your age?

Person 1:…. It's like this miss… they are young…. But some of them are…even what they teach…. Sometimes for us…

Person 2:…. There are problems….we also have problems…… most of them are Tamils….. so, we don't understand…
Moderator: Those are the things you need to talk about. The Kilinochi campus not having a single Sinhalese staff member is the main problem you should talk about. You can't understand what they are saying and they can't understand what you are saying….

Person 1:…. Sometimes we don't even get an opportunity to talk …. And we don't even try to talk….

Person 2:…..(unclear)….

Several talking together……

Moderator: Miss, if you can talk to the admin about this… please do it in a way that the 4 of us don't get suspended….

Several talking together……(unclear)…

Moderator:… for what?....

Person 1:….. they suspended…..

Moderator:… for doing what?

Several talking together …… (unclear)…..

Moderator: because you said that there are only Tamil and no Sinhalese staff members, or what?

Person 1: Madam, we are only a few Sinhalese students…. We don't have so much power…. If this recording or the transcripts go to them….

Moderator: No! No! I told you these won't go to them.

Observer: what is the problem?

Moderator: They are scared that we will give this to the administration.

Person 1: ….No…no, there's nothing like that…

Moderator: Does this faculty have alcohol, drugs and Marijuana? Don't say there isn't.

Several together….. yes, those things are there…..

Person 1: …we don't want to lie and say that those things are not there…..

Several talking together…. (unclear)…..

Moderator: Is it true that they say, that it is more among the Sinhalese students?

Person 1: So, madam, this is a common problem in Sri Lanka….. the people who bring these things into the country are Muslims are a well know fact… It is a very well accepted fact…. Several talking together…..

Person 2:…. So, madam, it's not just not in our university….

Moderator: So, As good students, can't you stop the ones who do these things?

Person 1:… to be honest, it's the Tamil students…. We have some connections with the Tamil students but… we are the outsiders, isn't it…. These students could know thugs and others….

Moderator: No child, what I'm saying is that you as a rep (of the union), can't you stop the Sinhalese students from doing these things and correct them?

Person 1: I can madam…. That can be done….

Person 2: …..if a program like that was done…. We even don't like it if our friends, stray and go on the wrong path…

Person 3:…… we should study …. Not let someone die…. What we try to do is, if there is someone like that among us like that….. even we have friends like that, I'm not saying that we don't…. we also somehow try to get them into a good condition… but the problem is the supply….

Person 4:….. it's the supply…..

Person 3:……even what the government is doing is trying to stop the supply….. we give them our fullest cooperation…. We also try to fix them….. if it's someone we know we try to help them…

Moderator: So, these students after using drug, don't they do wrong things? Threatening other students, fighting…..

Several talking together…. (unclear)…..

Person 1: ….to tell you the truth, I'm happy to tell you that in our batch, along with drugs…. There is this thing called "(unclear)"…. Those things are not there… there are not many things below that…. only things like Marijuana, alcohol and cigarettes are used….. then madam…..

Moderator: …then where do they have the money to buy these thing? Students who can't afford the lunch parcel, how do they buy these things?

Person 1: Something that I have noticed is that a lot of these people…. When we go to our home towns and talk…. It's a lot more cheaper here (alcohol and drugs)….

Moderator: what I'm asking is, the students who say they don't have money to buy a lunch packet, how do they afford it?

Person 1: Miss, imagine this… even a daily laborer will drink alcohol at the end of the day…..

Moderator: The daily laborer, does work and earns a wage….

Several talking together…..(unclear)…

Person 1: ….. the person who wants to do something will do it somehow (aggressively)…

Person 2: …they do different things to find money….

Moderator: What sort of things?

Person 1: …I don't know (aggressively)…

Several talking together…. (unclear)…..

Person 2:… do different things…

Person 3: …what can we do?……

Laughing….

Moderator: I'm just asking out of curiosity. What can you small boys do to earn money?

Person 1: …we of course don't do anything….

Person 2:….Some give tuition classes…

Person 3: ……give classes….

Person 4:….. some do laborer jobs….

Person 1:…. Go in the evening and work in companies….

Person 2: ….work in companies and sell insurance in the evenings….

Person 3:….. do whatever job they can do….

Person 4:…. Some go and work in the paddy fields….

Person 1: …..If we were in a university like SJP…. We could have worked in shops…. We could have washed dishes…

Person 2:….. but in places like this…. there aren't many things to do…

Moderator: So, boys work like that?

Person 1: Yes, you can do something like that….

Person 2:…. We don't have many opportunities…..

Person 1:….. Yes, some do part time jobs….

Several talking together….

Person 3:…. So, go home on weekends and work….

Person 4:… they do something and…..

Several talking together….

Person 1:…. But when we go home for weekends we do house work….

Moderator: ok, ok. We are very happy to be able to talk to you. We have heard that the students in Kilinochi are taken to a temple.

Person 1: yes….

Several mumbling together… (unclear)…

Moderator: We have heard that you go there to rag students?

Person 1: No, no…..

Several talking together in a softer tone…. (unclear)….

Person 2:….. no….. we don't…..

Person 3:…..no….. no ragging….

Moderator: In the weekend.

Person 1: …..No, miss….

Several talking together…..

Person 2:….No…

Several talking together….

Person 3: ……a lecturer comes with us….

Person 4: …… there is a lecturer in the ______ (unclear)… it's with his leadership that the temple is getting developed…. He's always there… he won't let us even lay a finger…

Person 1:…… we also can't……

Person 2:…. The temple is…. We don't want to do anything there…. There is…..

Moderator: Do the female students go there?

Person 1:….. to think even…..
Person 2:…. No, girls don't go there….

Person 3:….. a miss (teacher) or someone who supervises goes…..

Person 4: …….they won't send female students by themselves…..

Person 1:…… there are a lot of male students there….

Moderator: Oh, so, there are only boys?

Person 1: ……More boys in the sense….. the work is still going on so there are males working on the construction…

Person2 : ….there are a lot of works there….

Person 3:… then you can't send female students alone…..

Person 4: …….Madam, I think what your saying is….. hmmmm…. I can remember our juniors…. Some girls went to do a Buddhist ritual (Bhodi pooja) and couldn't go in….. that must be the occasion you are talking about….

Moderator: ….I don't know about this Bhodi pooja incident…. But I have heard about ragging in this temple. I have heard that when students want to go home for the weekend, that seniors won't let them go home.

Person 1: To the temple?

Moderator: No child, to their homes.

Person 1: We have not said anything like that, madam…..

Several talking together……(unclear)…..

Person 2: …..when they ask the warden……

Person 3: ……have to make a request……

Person 4:…… the senior lecturers had made a request…

Person 1: …….made a request to keep the students….

Several talking together… (unclear)…

Observer: we can't hear what you are saying, if you are all talking at the same time….

Several students mumbling….

Person 1: One of senior lecturers had made a request asking us to participate if possible….. that's why we told the juniors also to come and participate……that we are all going…. We and our "aiyas" are all going…. So, that they should participate in the same way….
Moderator: That day then all the junior students would have attended. The junior female students also would have come, right?

Several together…… yes…..

Moderator: But you said that you haven't even seen the junior students…..

Person 1: …..yeahhhh…..

Several talking together……

Moderator: but, you said that you haven't even seen them.

Several together….

Person 1: ……can't say we haven't seennnn….

Person 2: …….on one day…….

Person 3:…. Haven't spoken to them……

Person 4:….. we have seen them but……

Person 1: …..we haven't had a chance to talk to them…..

Moderator: Ahhhh….

Person 1: …..just like in school….

Person 2:….. we have seen …..

Person 3:…….we don't get a chance to talk to them…..

Person 4:…… there is nothing….. can't even talk to them….

Person 1: ….we have seen them but haven't gotten a chance to talk to them…..

Moderator: So, you said that this campus is like a lonely desert. So, what do you do?

Person 1:….. the boys of course…… play cricket in the playground in the evenings….

Person 2:……. The girls come when they finish the lectures…….. to tell you, there is work to do…. Unlike other universities, the Jaffna university has a big curriculum…. And the

definitely cover that curriculum…. To tell you madam….. even today evening when we go back to the boarding house… we have a lab report to write….

Several talking in the background…..

Person 2:…. With that amount of work…… the boys to go and play a cricket match and go back and…..

Person 3:….. we also have to do something fun madam….. otherwise how can we……

Person 2: …. We need to cover the subjects…..

Person 4:….. when we have a function…. With the greatest of difficulty…..

Several talking together…. (unclear)…..

Person 1:…… to get permission…..

Person 2:…….we wrote more than 5 letters to get permission…..

Person 3: …then they said to give the juniors tea party…. In order to do our cricket function….

Person 4:…… then we wrote the letters yesterday to do the tea party today….

Person 1:….. we only got the letters of approval the day before to do the Tea party….

Person 2:…. To do a function like that… we don't get permission…..

Moderator: Why is that?

Person 1:…… that's the way…..

Moderator: ….the student union….

Several talking together…. (unclear)…..

Moderator: to do student events, there is no racial…..

Person 1: No madam, it's like this….. our union….. we haven't even been able to register our union yet… our seniors, us and our juniors…. Hmmm… we get together this evening….. the others (Tamils) ….. they are one group….

Person 2: …….we can't get their cooperation for anything……

Person 3:….. you can get the help from the other faculties but…… here in Jaffna….

Person 4: …..with the distance and things…… it's difficult…..

Moderator: .. It takes you about 1.5 hours, isn't it? (to the main Jaffna campus)

Person 1: ….then there is a majority of Tamil students there…. These ones have asked to register a union… with a Tamil secretary and a Tamil president… If we have an election and try to appoint a president and a secretary then we can straightway appoint an Sinhalese president and secretary…. But we won't do that…. So without doing that we change every year…we have a Sinhalese president one year and the next year a Tamil president…

Person 2: …..we also alternate the secretary…..

Person 1: …..even after doing that…. what can we say….. we still have no recognition….

Person 3: ……they won't accept it as our union…… they think of it as a brotherhood…..

Person 4:….. that's how they accept us……

Person 1:…… they won't accept the union…..

Moderator: So, don't you go the main Jaffna university?

Person 1: We go there for sports….. for things like that but for other things they won't send us….

Several talking together….

Person 2:…… with our academic work also we don't have time….

Moderator: Thank you very much for talking with us. What my advice to you is to stop thinking about the world and think how you can progress and go forward in life. Help the others also.
