# Supplementary material for: Ragging as an expression of power in a deeply divided society; a qualitative study on students perceptions on the phenomenon of ragging at a Sri Lankan university
Source: PLoS One. 2022 Jul 11;17(7):e0271087. doi: 10.1371/journal.pone.0271087 (PMC9273066; doi:10.1371/journal.pone.0271087)
Supplement: Attachment — Submitted filename: Response to Reviewers.docx [file pone.0271087.s005.docx]

**Response to Reviewers**

Dear Marcel Pikhart,

Thank you for giving us the opportunity to submit a revised draft of our manuscript titled “We are considered to be dust”; a qualitative study on students perceptions on the phenomenon of ragging at a Sri Lankan university to PLOS ONE. We have addressed the additional requirements stipulated by the journal as well as the reviewers comments. The journals style requirements have been followed and the ethics statement is only included in the methods section. We have also attached the interview guide as supporting information. We could also like to clarify that the corresponding author is a doctoral student, affiliated to Uppsala University, Uppsala, Sweden as mentioned in the manuscript.

We appreciate the time and effort that you and the reviewers have dedicated to providing your valuable feedback on our manuscript. We are grateful to the reviewers for their insightful comments on our manuscript. We have been able to incorporate changes to reflect most of the suggestions provided by the reviewers. We have highlighted the changes within the manuscript. Please see below, in blue, for a point-by-point response to the reviewers’ comments and concerns. All page numbers refer to the revised manuscript file with tracked changes.

**Reviewer 1#**

Thank you very much for taking the time to assess our manuscript and provide your valuable comments. We have addressed all the concerns you have raised to the best of our abilities.

- The manuscript does not meet with the sections of the journal.

We thank the reviewer for pointing this out. We have revised the manuscript accordingly to comply with the journal format throughout the manuscript.

We have also changed the heading Background to Introduction (page 3, line 46).

- Introduction/background seem very long and confused. This journal only has an introductory section. This structure looks like for another journal. The claims are not properly placed in the context of the previous literature, since there are references that show contexts or social situations from more than 20 years ago.

We have changed the word background to introduction along with a few other formatting changes as mentioned above.

We have changed the introduction (pages 3-5) to provide a better contextual background as we also received a similar comment from another reviewer.

We agree with the reviewer that some references date back 20 years but there is a strong cultural and historical context to the occurrence of ragging that we wished to highlight. At the same time this is an under researched area in Sri Lanka and there is a lack of current research that can portray the social situation and context.

- Materials and methods section: “Qualitative research is an approach for exploring and understanding the meaning individuals or groups ascribe to a social or human problem” (Creswell, 2013b, p. 3). In this case, the theoretical approach has not been indicated, is it ethnography, phenomenology, grounded theory? A correct qualitative study needs a strong theoretical foundation.

Thank you for pointing out our oversight in not mentioning the theoretical approach. We have now included it in (page 7, line 198). Our reference for the phenomenological approach aligns with Creswell that states that, “Qualitative research is an approach for exploring and understanding the meaning individuals or groups ascribe to a social or human problem”.

We agree that qualitative studies need a strong theoretical foundation and you will find our theoretical framework in figure 1.

- Research design needs to be further explained, as well as other subsections of the methods. Following the journal recommendations, I kindly suggest reviewing the CASP and COREQ checklists to ensure the quality of the data reporting. For example, the researcher's relationship with the participants, what grade and semester were the students in?

That was a very good suggestion to align with the COREQ checklist. We have revised our methods and materials section using the COREQ checklist. The changes are visible on (pages 8-10, lines 216-278).

The researcher had no relationship with the participants and further details have been added to the relevant section (page 8, line 220-228). To maintain the anonymity of the participants, we did not inquire which grade or semester they were currently studying-in but all participants were chosen from the 2^nd^ and 3^rd^ year of study (page 8, line 217-220).

- I suggest that only current references are used in the discussion, due to changes in this phenomenon in recent years (for example, the introduction of new technologies has changed the type of harassment in different contexts).

We appreciate the reviewer’s suggestion and agree that it would be better to use more current references, unfortunately since there is little research available, we are unable to do so. We share your belief that technology has enhanced the ability to be bullied and there were instances stated by our participants where mobile phones were used. It is our hope that this seminal paper will lead to deeper and broader inquiries into the myriad of ways that ragging is carried out in Sri Lanka. The public health of youth and particularly the increase in mental health issues, demand this.

- You should mention limitations

Thank you for pointing out this omission. We have rectified this mistake and added the limitations of our study (page 32, lines 929-940).

**Reviewer 2#**

We appreciate your time and effort in providing feedback on our manuscript and are thankful for the insightful comments. We have tried our best to address all your comments and concerns.

- There is a total absence of contextualization of the study, the introduction does not justify or theoretically support the research, they should include the hypotheses of the work.

Thank you for this feedback concerning the lack of context in the introduction. We have rectified that shortcoming by reorganizing the introduction section and adding more context as per the reviewer’s suggestion. These changes are reflected in the revised introduction section (pages 3-5). We appreciate the suggestion to include a hypothesis, however as this manuscript is an explorative qualitative study, as in most studies of this nature, there is rarely a hypotheses as it is more about discovery.

- The method is not explicit enough. It does not give details of the data analysis and the software used for the analysis of the transcripts. Likewise, you should expand the information on the characteristics of the participants.

We agree with the reviewer and have updated the methods section to provide more details and information. These changes can be seen on (page 8, lines 214-220 and page 8, lines 253-278). No software was used for the analysis of the transcripts. The diverse research team read and re-read the transcripts after which we spent several days altogether mapping, defining, and re-defining the themes with the help of a whiteboard as per Braun and Clark’s description of carrying out thematic analysis.

- In the discussion section there is no comparison of its results with the initial contextualization. Personally, I am not in favor of using literature in the discussion that has not been previously mentioned in the introduction, since it transmits an incomplete review of the state of the topic investigated, there are several of the citations included in this section that have not been previously cited in the introduction.

Thank you for this observation. We have changed the introduction (pages 3-5) to improve the context as well as changed the discussion (pages 26-31), thereby allowing a better comparison of the results in the discussion section. In this explorative study design, new findings emerged that required searching for new literature that was then included in the discussion. Prior to analysis of the findings, this new material was not relevant to the introduction.

- It should include the limitations and future lines of research before the conclusions.
  The conclusions can be expanded to include and / or give examples of how this information can be useful to the population and the field of education. Authors must make explicit reference to the practical application of the results obtained (this is one of the strengths of the manuscript), paying special attention to the possibilities offered by the data for the design of interventions. These limitations should be addressed with a view to the possible publication of the manuscript.
- We agree that practical application is our eventual goal. We have strengthened the manuscript to include the reviewers suggestions, which will be reflected on (pages 32-33, lines 942-949). As this was an explorative study, these results cannot be generalized although in qualitative there is a possibility that some of the findings/ideas are transferable to similar settings/contexts but that would be up to the different individual readers to draw any conclusions there. Therefore, the practical application of the results and the design of intervention require further research. This will certainly be addressed in our upcoming research currently underway.

The limitations of our study have been included to the manuscript

(page 32, lines 929-940).

**Reviewer 3#**

We thank you for taking the time to identify certain issues in our manuscript and providing us with the opportunity to strengthen the paper.

- Introduction/Background/Theoretical Lenses:
  This section needs more of the explanatory material provided in the Discussion. Please use the introduction to expand more on issues of how ragging is perhaps culturally specific, even though it does have analogues in other cultures. Indeed, please move up much of the information on Sri Lankan society, especially the social classes, that is given in the Discussion. Those facts help the reader to make sense of the data you report and you should give more of that information in your Introduction to help the reader contextualize the findings. You don't need to talk about all of the details in the introduction, but they do add information that would be helpful to a reader of the introduction.

Thank you for bringing this to our attention what lacked in our introduction to make this paper a comprehensive read for those unfamiliar with this context. We have reorganized and revised our introduction (pages 3-5) and discussion (pages 26-31) as suggested to include more extensive information concerning Sri Lanka’s history, society, and culture.

- Results:
  The thematic analysis is interesting, but the reader has no sense of how representative it is of the sample of focus groups or individuals. Please go back to the data and make tables to report the number of times each of these themes were mentioned or discussed, variation by gender, and ethnicity. You can also use such data to conduct a grander analysis, like a cluster analysis for the themes. The reader is curious, does the expression of some of these themes within the focus groups overlap with the expression of other themes? That is, are the themes correlated? If so, you could report and discuss such potential patterns in the data. At present, the reader has a rich picture of the general findings and concept of ragging and its social-economic expression at the University, but more concrete numbers would greatly enhance the paper.

We appreciate the reviewer’s suggestion and agree that it would be interesting to demonstrate number of times each of these themes were mentioned or discussed, variation by gender, and ethnicity however, such an analysis is beyond the scope of this introductory and exploratory paper. We hope that this explorative/scoping study will serve as base line information to feed into our planned quantitative study where your idea a grander analysis would indeed provide more concrete numbers.

The themes of our study were inductive and data driven. In the method of Thematic analysis as per Braun and Clark, themes can overlap and are not mutually exclusive as in content analysis. This is demonstrated in figure 2.

- Conclusion:
  Please reduce the space given to the Discussion (currently 6 pages) and insert much of that important information in the Background/Theoretical Lenses (which are only 3 pages combined).

Thank you for your comment, we have enriched the introduction and reorganized the discussion section as per the reviewer’s suggestion. Unfortunately, we were not able to reduce the number of pages of the discussion since we had to address the comments from the other reviewers on the discussion, and we also added the limitations of our study.
