# Supplementary material for: Ragging as an expression of power in a deeply divided society; a qualitative study on students perceptions on the phenomenon of ragging at a Sri Lankan university
Source: PLoS One. 2022 Jul 11;17(7):e0271087. doi: 10.1371/journal.pone.0271087 (PMC9273066; doi:10.1371/journal.pone.0271087)
Supplement: Attachment — Submitted filename: Response to reviewers.docx [file pone.0271087.s007.docx]

**Response to Reviewers**

Dear Wolfgang Himmel,

Thank you for your review of our manuscript. We appreciate the time and effort you have taken to provide insightful feedback as well as your useful comments. Your comments and concerns have been addressed below. We accept the opportunity you have offered to submit a revised draft of our manuscript with the revised title: “When a person can’t obey, he is subjected to violence”; a qualitative study on students perceptions on the phenomenon of ragging at a Sri Lankan university to PLOS ONE. Attached, please also find the completed COREQ guide as requested and additionally, more examples of ragging, as supporting information in an Appendix.

The interview guide was developed by the research group as a part of the study and is not copy righted. The interview guide was only developed in English as all the moderators were fluent in English and did not require the interview guide to be translated.

We attached the transcripts of the 17 FGDs as requested as supporting information S4_4.File. We would once again like to point out the sensitive nature of the transcripts. The FDGs conducted among the students were divided by ethnicity and gender, and most often the participants belonged to the same faculty. During the FGDs the participants also discussed certain issues which would increase the chances of them being identified. Some of the issues discussed where regarding the lecturers and the university administration which could cause problems for the students if they were to be identified. These indirect identifiers may risk the identification of study participants, which would be a breach of our confidentiality agreement, as well as our ethical approval.

Changes made within the manuscript are highlighted below in blue along with a point-by-point response to your comments and concerns. All page numbers refer to the revised manuscript file with tracked changes.

1. My first major concern refers to the study question. Although the study question seems clear on first view – the perception of ragging, seen from the student perspective –, the sample of students that you choose for your study makes the issue ambiguous. One could say, it is a group ‘in - between’. They are no longer freshman who experience ragging at the very moment; they are not 'old' students who are 'entitled' to rag other students. Therefore, some of them may have experienced ragging – we don’t know exactly; some may have observed ragging being in the position of a 'neutral observer' or a 'former affected person' – we don’t know exactly; some seem to prepare themselves to become ‘ragger’ themselves in the near future – we don’t know exactly. But throughout the paper you write more or less as an advocate of the 'victims' (see, for example, lines 114 -118).

Thank you for pointing this out. We have revised the manuscript accordingly to make it clearer. As health care advocates we often tend to divert attentions towards the victim’s plight but we agree that this is a public health problem where all students suffer, as you correctly indicate.

These changes are visible on page 8 line 223-224 and in the introduction in page 6, line 136-138.

I can understand your argument that you did not want to discuss this matter with young students, who may still suffer from ragging or are reminded of this terrible experience in the group discussion, you cannot claim your study addresses the victim perspective (line 116). While this seems to be a limitation of your study, you could also make it to a strength of your study. The experiences of your 'in – between' group may be an optimal opportunity to study how a procedure, which is so burdensome for many young persons, becomes an established and accepted institution in students' life. So, you should clarify what you exactly expect from your study – it is definitely not the experience of ragging; in this case young students should have been enrolled. So, you should precisely define your scientific interest (e.g., to understand how ragging invades student life and culture). Of course, you can criticize the practice of ragging in the Discussion but you should not use the study participants for this purpose; they are much more ambivalent.

As ragging is a very sensitive issue in Sri Lanka, we had to be very cautious in our selection of participants. We also realized that we had not mentioned that perpetration of ragging occurs from the second year of university attendance, onwards and have now included this information in the introduction (Page 5, line 109-111). In order to better define/clarify our scientific interest, we have amended the aim (Page 6, line 141-143) of our study.

This paper is a part of a larger study on ragging where we have found the prevalence rates of ragging among students at Jaffna University to be over 50%. We have another qualitative manuscript exploring the perceptions of lecturers and other key persons attached to the University highlighting that angle.

2. My second major concern refers to what you define as the 'overarching theme' of your analysis: "ragging as a means of communication". It was you, not the students, who stimulated a communication about ragging. Even more important, the students first reaction in the group discussion was to emphasize that ragging did not occur (line 391). It seems to be a secret or hidden practice, but definitely not a ‘means of communication’. Perhaps you mean something else but your expression is in any case misleading. By the way, I’m not sure whether you need an overarching theme at all. Isn’t it sufficient to define your subthemes as main themes and to present them as your results?

Thank you for your comment. Following a discussion between the authors, we have decided to change the overall theme to “Ragging as an expression of power” which maybe better suited for the study. (Fig. 2. Main theme and subthemes and page 11, line 277)

In this case, it may be a good idea to start the Results section with the issue of 'secrecy' and your overall observation that all groups denied ragging in the beginning of the discussion. This is really striking, given the formative experience of ragging. Obviously, older students try to forget or deny it. This could be the first important result and could later lead to an interesting discussion how difficult it is to make ragging 'public'.

We have made changes in the order of the results section to start with the “Veil of secrecy and silence” as it is an important and interesting aspect of ragging, as you underscored in your comments (Fig. 2. Main theme and subthemes and page 11, lines 288-309).

3. Please check the logic of your themes and how you present them in the Results section. I see a couple of inconsistencies so that a re-arrangement may be required.

Thank you for your comment. We have revised the order of the results in a manner that might be easier to follow. (Fig. 2. Main theme and subthemes)

(a) ‘Trivializing violence’ (lines 415 ff.) is not a subtheme of "Veil of secrecy and silence".

We have changed “Trivializing violence”, to belong to the subtheme “A society with deep divisions” as it is a culturally learned response. (Fig. 2. Main theme and subthemes and page 22, line 620-635).

(b) Although the examples under the subtheme "Rigid norms" are interesting, I’m not sure whether all of them belong to the main theme "A society with deep divisions". They deal, for example, with things like a general lack of protection of women (why is this a matter of ‘rigid norms’?). I think, other interesting examples, too, have nothing to do with ‘rigid norms’, such as equalizing (lines 503 ff.) or denying ethnic clashes (lines 536 ff.). Please check the main theme and the subthemes so that they are consistent.

We have thought through your suggestion and revised the subthemes as well as removed some sections which we agreed could lead to confusion. Societal/Cultural norms are the foundations of how people comport themselves in groups. When there is an inflexibility between groups, there tends to be inequalities and higher rates of conflict. That said, the lack of protection for women occurred in small pockets and as it was not mentioned widely in the study so we decided to omit it. (Fig. 2. Main theme and subthemes)

(c) It is also not clear how the students’ feeling not to be heard by the society and the university (lines 542 ff.) or the feeling of insecurity (lines 571 ff.) is related to ragging. It is surely an important aspect, especially in the life of minorities but the authors should help readers to understand why these feelings and experiences may lead to ragging or may cause some students to accept ragging or to deny it. This should happen at least in the discussion.

Thank you for this observation. We realize that insecurity, might not be directly related to ragging and we have removed these sections. (Fig. 2. Main theme and subthemes)

(d) I would recommend to display “The students’ recommendations” (lines 585 ff.) as a main theme in Figure 2 and to present it as a main theme in the text so that readers can better follow your complete analysis and identification of themes.

We appreciate the reviewer’s suggestion and agree that it would be better to have “student recommendations” as a main theme. (Fig. 2. Main theme and subthemes and pages 24-25, lines 668-840)

4. The Discussion needs much more structure. You should start with a short and precise “summary of main findings”, no longer than 8 to 10 lines. I would suggest to stress the aspect of ambiguity or ambivalence towards ragging as the most important aspect (at least for me). The first sentence (lines 611 f.) Is superfluous; the last sentence (lines 617 ff. should be moved to the "Strength and Limitations" section in the Discussion. After the short summary you could go on with three major sections perhaps clearly marked with a subheading:

We have made changes to streamline the discussion as per your suggestion, and added subheadings to demarcate the three major sections for better flow. We combined the positive and negative ragging under the subheading of “Spectrum of ragging”. (page 25-31)

- Why ragging is positively perceived? (page 30, line 1125-1158)

- Why ragging is negatively perceived? (page 31, line 1160-1168)

- The wider context of ragging: Sri Lanka and its university culture (page 26, line 1002-1122)

5. I like your theoretical model of violence displayed in Figure 1, but I see two problems. I miss an “integration” as you claim with the Figure. Some arrows in the Figure and the pure statement “structural violence, intersectionality and social dominance theories are intricately linked” in the text (line 144) are not an integration. Moreover, you should use this model in a more creative way to explain ragging in societies like Sri Lanka. For example, as far as I understand, you say Sri Lanka is a violent country and unsatisfactory conditions contribute to student violence (line 657 f.). I agree, but one could ask, how? You say, ragging is violence and at the same time, ragging seems to be protest against inequalities and violence of the society. It is also remarkable that it is not the upper-class students (or only a few) who practice ragging but those at the other social end. Therefore, I would very much appreciate if you make a second attempt to use the model for an explanation.

We agree that there was much room for improvement in our model to demonstrate the integration of the three theoretical frameworks. These changes will be visible in Fig.1. Integrated theories on ragging in society.

6. In addition to reference 22, you may also refer to this just published study that shows the strong association of ragging and suicide: https://pubmed.ncbi.nlm.nih.gov/35025905/

Thank you for the reference. We have included it in our manuscript (Reference 24, page 5-6, line 131-133).

7. Did you attach the COREQ statement for qualitative studies and did you fill in the page numbers, as requested?

We did not attach the COREQ guide statement for qualitative studies although we followed the guidelines. We have now attached it under supporting information. (S_2. File)

8. Line 399: One or two examples how seniors named juniors could be helpful to better understand this humiliating procedure.

We appreciate the suggestion to include an example of how the seniors humiliate the juniors and have included it (page 11-12, line 295-309 and page 13, line 333-343).

9. It would be generally helpful to give more examples (in form of an Appendix) how ragging is executed. It reminded me of practices and rituals of student leagues (“Burschenschaften) in Germany. Here, too, the younger ones have to be servants of the older students, are treated very badly, much alcohol is on the way and for some student leagues it is a special pleasure to hurt one another, using swords and, thus, to demonstrate masculinity. These practices are well known and also documented via media so that we have a very good idea of these rituals. Perhaps you have also some material to present examples in an Appendix that makes ragging easier imaginable for Non-Sri Lanka people.

Thank you for this recommendation to include examples of ragging to better understand this practice especially for non-Sri Lankans. We have added several newspaper articles and YouTube links as a supporting document. (S_3 File)

10. Line 729 ff. Of course, the practices you describe here are humiliating women. But please, consider that it can also be a hard expectation towards male students to behave in such an inhuman way. I do not want to exonerate males from accusations but some of them may also feel in a dilemma.

We share your belief that gendered expectations harm both women and men and we have rephrased this sentence (page 28, line 1077).

11. Line 785: I agree with your last plea but, following the students’ experiences, should it not read that it needs deep reforms in the university and the society to successfully combating ragging?

Thank you for your suggestion, which is also our belief and does strengthen our closing statement (page 33, line 1379-1381).
