# Supplementary material for: Ragging as an expression of power in a deeply divided society; a qualitative study on students perceptions on the phenomenon of ragging at a Sri Lankan university
Source: PLoS One. 2022 Jul 11;17(7):e0271087. doi: 10.1371/journal.pone.0271087 (PMC9273066; doi:10.1371/journal.pone.0271087)
Supplement: Attachment — Submitted filename: Response to reviewers.docx [file pone.0271087.s008.docx]

**Response to Editor**

Dear Wolfgang Himmel,

Thank you for your very thorough reading of our manuscript. We appreciate you taking the time as the editor, and for all the insightful comments and recommendations you have made. We you are grateful for the opportunity to refine our manuscript to PLOS ONE standards.

Changes made within the manuscript are highlighted below in blue along with a point-by-point response to your comments and concerns. All page numbers refer to the revised manuscript file with tracked changes.

1. It was a good idea to change the title, especially since the previous title was difficult to understand for readers who are unfamiliar with the background of Sri Lanka. However, I’m afraid, readers will also have problems to understand the meaning of the new title and its association with ragging, apart from the fact that “he” in the title is suboptimal and “(s)he” is not accurate … What do you think about including the topic of ‘power’ in the title, for example this way:

“Ragging as an Expression of Power in a Deeply Divided Society; — a qualitative study on students’ perceptions of ragging at a Sri Lankan university”

Thank you for your suggestion. We agree that the title you suggested, better captures the understanding of our manuscript’s content and have revised the title accordingly. (Page 1 line 3-5).

2. Although you tried to adapt the Abstract to the new version, it reflects the manuscript only to a limited degree. For example, main themes and sub themes are not well presented in the Abstract; also, the Discussion in the paper is not represented in the Abstract.

First of all, I would suggest to structure the Abstract with the help of the usual subheadings, i.e., Introduction, Methods and so on.  Please be aware, PlosOne allows 300 words (you have currently about 250 words).

The last two sentences of the Abstract are very similar, so one of them could be deleted. Instead, there would be some more room for 1 or 2 sentences of a "Discussion ". Moreover, to be more precise, I would suggest to change one of the reported results in the Abstract this way:

INSTEAD:

The findings revealed how students used ragging as an expression of power to initiate order and as a way to express dissatisfaction towards social inequalities occurring within the larger society as well as facilitate bonds between university students.

BETTER:

The findings revealed how students used—or perceived—ragging very differently: as an expression of power to initiate order or as a way to express dissatisfaction towards social inequalities occurring within the larger society or to facilitate bonds between university students.

We appreciate your suggestions on refining the abstract for more clarity.

Your observation of the repetition in the final lines of the abstract prompted us to delete one of the last sentences. (Pages 2, line 42)

We agree that it would be better to rephrase the findings in the abstract as recommended by the reviewer. (Page 2, lines 36-39)

While we appreciate the suggestion to include a structured abstract with subheadings, most qualitative work published in PLOS ONE had unstructured abstracts and we thought it was best to adhere to the general rule.

3. line 117: the word “ragging” is missing, isn’t it? (my “lines” always refer to the ‘clean’ version.)

We have rectified the omission and now included the word “ragging” (Page 5, line 127).

4. For me, the lines 120 to 127 are crucial because they present and justify the Aim of the Study. However, I do not find the passage completely convincing and could imagine the following passage instead, based mainly on regrouping the sentences:

INSTEAD:

It is increasingly imperative to address this serious public health problem that profoundly affects all students, not only victims but also perpetrators and by-standers. Ragging has a potential deleterious impact upon society’s younger generations and their university years intended for building intellectual capacity. Educating youth in a safe space is essential, particularly for its subsequent contributions towards the country’s future. The aim of this study was to explore the perceptions of students concerning the phenomenon of ragging, and to understand how ragging affects student life and culture at the University of Jaffna.

BETTER:

Educating the youth in a safe space is essential, particularly for their subsequent contributions towards the country’s future. It is increasingly imperative to address this serious public health problem from the students’ perspective and to understand how it affects all students, not only victims but also perpetrators and by-standers. Since ragging has a potentially deleterious impact upon society’s younger generations and their university years intended for building intellectual capacity. The aim of this study was to explore how students perceive ragging and how ragging affects student life and culture at the University of Jaffna.

You may even find better alternatives …

Thank you for your suggestion. We have revised the aim, taking your suggestion into consideration and rearranged the sentences to make the aim more succinct. (Page 6, lines 133-139).

5. From the history of the submission, I know that the chapter “Theoretical lenses” was desired by the reviewers for the Introduction. Although interesting, it may surprise the reader at this point, because after the formulation of the Aim of the Study, the Methods chapter is expected. That said, it would be much wiser to present the Theoretical lenses right there, namely in the Methods chapter, preferably starting at line 227 ff. What do you think?

While moving the Theoretical lenses into the Methods chapter, you should make it a bit clearer at this point that you used the Theoretical lenses to sensitize yourself for the data analysis. You have already mentioned this very well at the end of the Discussion; however, this argument should (also) come up already at the beginning of the Theoretical lenses chapter so that readers are informed about the significance of this chapter.

Thank you for your comment. We have thought through your suggestion and made some changes to both the Theoretical lenses section (Page 6, lines 143-144) and the Methods section (Page 10, lines 242-243) which will enable the reader to comprehend this section better.

Since it is common practice in qualitative studies to include the theoretical lenses at the end of the introduction, we have adhered to this format. Furthermore, theories most often evolve from the literature review and even when not made explicit, are ever-present throughout the work. For example, phenomenology is understood as foundational to qualitative approaches. There is an interplay between the theoretical lenses, the introduction to the literature, and the methods section. We believe, including the theoretical lenses at the end of the introduction, helps sensitize the reader early in the text thereby laying the groundwork in which to view the methods section.

6. Due to the many (sub)headings in the Results chapter, it is not always clear which of them are the four main topics. I would therefore simply suggest (for lines 251, 278 etc.) to add: Subtheme 1: …, Subtheme 2: …  etc.

We agree that including subheadings in the Results section would make the main topics/themes more clear (Page 11 line 267, Page 12 line 295, Page 15 line 380, Page 18line 464, Page 23 line 588).

7. Line 283: I would suggest to start the first sentence with: For many students, ragging was part of …

We thank the reviewer for the comment and have made the necessary revision. (Page 12, line 296)

8. Of course, I do not want to go into the details of your analysis. However, the presentation of the themes is still not quite logical in some places — despite the otherwise very successful revision. This is most noticeable in the case of sub-theme 2. For me, there are two very different topics that are shoved into each other here, namely ‘Ragging lies on a wide spectrum’ and ‘ragging is a cyclical event that thereby establishes hierarchies’.

While the lines 283 ff. belong to the ‘spectrum’ topic, lines 288 ff. belong to ‘hierarchy’. Lines 307 ff. deal again with ‘spectrum’ over a rather longer period. Lines 352 ff. then deal with ‘hierarchy’; lines 371 ff. clarify in particular the cyclic character, lines 386 ff. the ‘hierarchy’. Lines 406 ff. again the ‘spectrum’, in this case, the positive side. Lines 417 ff. deal again with the ‘cyclic hierarchies’. This is the way I read/understand your quotes.

I urgently ask you to check here again structure and argumentation thoroughly, as well as the naming of the main topics. Perhaps a fifth sub-theme will be appropriate to avoid the confusion in presenting sub-theme 2. In any case, your terms/themes should be distinctive, convincing and simply to follow for readers.

https://pubmed.ncbi.nlm.nih.gov/35025905/

We appreciate your perspective here. We have revised the sub-theme titles by moving the section “Ragging lies on a spectrum” and making it a new sub-theme. We agree that it does impart more distinctive data than having it as part of the Cycle of Ragging. This will facilitate better understanding for the reader. (Fig. 2. Main theme and subthemes and page 12, lines 295-299).

We have also added a few sentences to clarify certain sections that may have been a little unclear. (Page 12, lines 298-453)

As recommended, a section found under “Ragging lies on a spectrum” has been moved to a more appropriate place in the text under “The powerful perpetrator” for easier comprehension. (Page 17, lines 432-439)

The section “Juniors dependent on seniors” is meant to illustrate dependency indicating subservience in the hierarchy. As the initial part of this section with its accompanying quote may had led to some confusion, we have omitted it from our revised manuscript to make it less ambiguous. (Page 17, lines 442-453)
